# Polarity Transduction Enables the Formal Electronically Mismatched Radical Addition to Alkenes

**DOI:** 10.1021/jacs.2c12699

**Published:** 2023-01-31

**Authors:** Subhasis Paul, Dario Filippini, Mattia Silvi

**Affiliations:** School of Chemistry, University of Nottingham, University Park, Nottingham NG7 2RD, United Kingdom; The GSK Carbon Neutral Laboratories for Sustainable Chemistry, University of Nottingham, Jubilee Campus, Nottingham NG7 2TU, United Kingdom

## Abstract

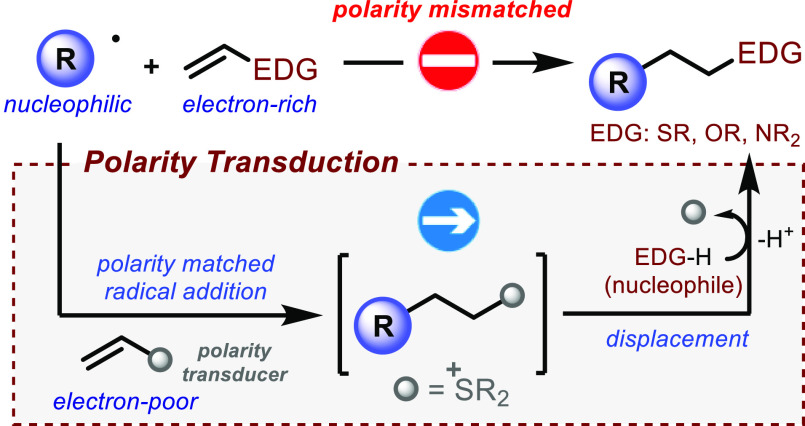

The formation of carbon–carbon bonds via the intermolecular addition of alkyl radicals to alkenes is a cornerstone of organic chemistry and plays a central role in synthesis. However, unless specific electrophilic radicals are involved, polarity matching requirements restrict the alkene component to be electron deficient. This limits the scope of a fundamentally important carbon–carbon bond forming process that could otherwise be more universally applied. Herein, we introduce a *polarity transduction* strategy that formally overcomes this electronic limitation. Vinyl sulfonium ions are demonstrated to react with carbon-centered radicals, giving adducts that undergo *in situ* or sequential nucleophilic displacement to provide products that would be inaccessible via traditional methods. The broad generality of this strategy is demonstrated through the derivatization of unmodified complex bioactive molecules.

Since its discovery and establishment in the last century, the olefin hydroalkylation reaction via intermolecular addition of alkyl radicals to alkenes^1^ has been used extensively in chemical synthesis for the construction of carbon–carbon bonds.^2^ Decades of research have refined the versatility of this process, stimulating the invention of new reactivity frameworks for the stereoselective synthesis of organic molecules,^3^ and inspiring significant advances in total synthesis.^4^ The recent development of efficient approaches to promote radical reactions under mild conditions—e.g., photoredox catalysis,^5,6^ electrochemical methods,^7^ and earth abundant transition metal catalysis^8^—has further enhanced the scope and the utility of this chemistry,^9−11^ providing new catalytic strategies to access enantioenriched chiral building blocks^12^ and to selectively functionalize complex molecules.^4^

Despite the synthetic value of these processes, the strict polarity matching requirements between the radicals and the alkenes involved represent a fundamental limitation to the generality of this chemistry.^1,13^ While favorable frontier molecular orbital interactions ensure a smooth reaction between alkyl radicals (typically nucleophilic) and electron deficient alkenes **1** (Scheme 1a, left), the addition of the same radical species to electron rich olefins **3** (Scheme 1a, right) is kinetically unfavored and does not practically occur, unless specific electron deficient functional groups are introduced within the radical center to induce electrophilic character.^1,13,14^*Thus, access to products**4**is not possible* using traditional radical chemistry. While a strategy has been developed to circumvent adverse polarity requirements in radical chemistry in the context of hydrogen atom transfer processes—i.e., polarity reversal catalysis^15^—no strategies have ever been developed to overcome the scope limitations of the radical addition to alkenes. Therefore, a general methodology to address the scope restrictions above would greatly enhance the field of radical chemistry, thereby stimulating further advances in various areas of synthesis.

**Scheme 1:**
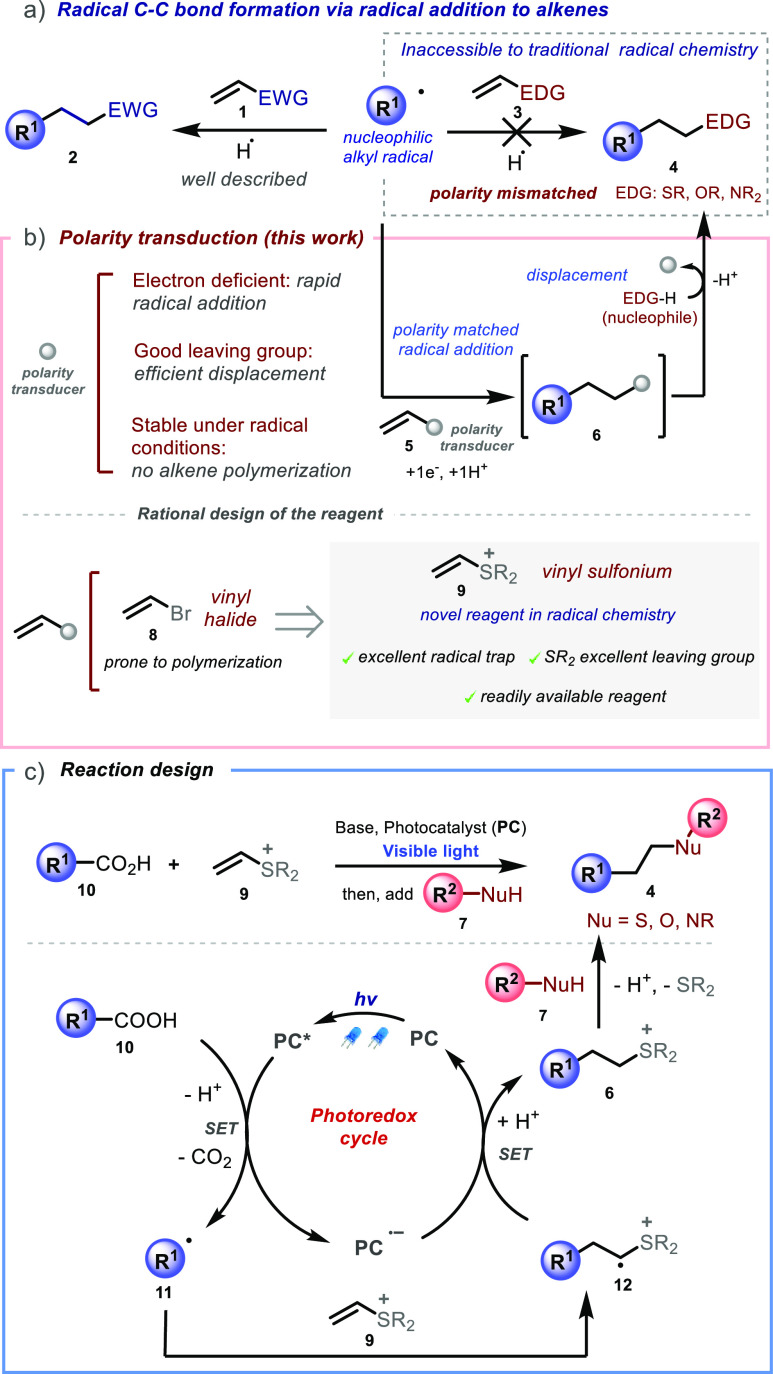
(a) Polarity Matching Requirements in Radical Addition to Alkenes; (b) Polarity Transduction Strategy to Access Polarity-Mismatched Products; (c) Design of the Photocatalytic System

We recently speculated that the strategic design of a composite process constituted by two elementary steps occurring *in situ* would circumvent the limitations mentioned above, allowing access to products **4** that are elusive to traditional methods. The strategy, described in Scheme 1b, uses alkene **5** equipped with a rationally designed functional group serving the role of a “polarity transducer”. Our polarity transducer would play the key role of converting the mismatched electron-rich polarity of the hypothetical π-system required to access products **4** into the matched electron-deficient polarity of the alkene moiety within **5**. Then, after rapid radical addition to give intermediate **6**, the polarity transducer group would be *in situ* displaced by a nucleophile, defining a practical alternative route to products **4**.

In designing a suitable polarity transducer functional group, we took into consideration the following requisites. First, it should be electron deficient in nature to subtract electron density from the neighboring π-system, thus lowering the energy of the corresponding lowest unoccupied molecular orbital (LUMO) to promote the addition of nucleophilic carbon-centered radicals.^1,13^ Second, it should be an excellent leaving group, to ensure rapid nucleophilic displacement to access desired product **4** from intermediate **6**. Third, its structural and electronic features should inhibit radical polymerization, a process typically occurring in vinyl halides **8**.^16^ Inspired by a seminal work from Barton et al.,^17^ we recently discovered that vinyl phosphonium ions readily undergo radical-based photoredox chemistry under visible light irradiation.^18^ Therefore, we surmised that structurally related vinyl sulfonium ions **9**, a species known to undergo polar reaction with nucleophiles,^19^ would participate in a radical conjugate addition reaction. As sulfonium ions are known to act as good leaving groups in intramolecular processes,^20^ and occasionally in intermolecular processes,^21^ we envisioned that they would constitute ideal polarity transducers for our strategy, given that an opportune reagent **9** and a suitable catalytic cycle were designed.

Following the hypothesis above, we conceived the photocatalytic process depicted in Scheme 1c. Photocatalyst-induced single-electron transfer (SET) decarboxylative oxidation of carboxylic acids **10** would generate carbon-centered radicals **11** in solution.^22^ Due to the inductive effect of the electron-deficient cationic sulfonium moiety,^23^ we predicted that nucleophilic radicals **11** would undergo selective addition to the terminal carbon of the pendant vinyl system within **9**. This would contrast with the reactivity observed with styrenyl sulfonium radical traps,^24^ in which addition occurs with opposite site-selectivity. Following the initial radical addition, the resulting electron-poor radical cation intermediate **12** would then undergo SET with the reduced photocatalyst (**PC**^**•–**^) to close the catalytic cycle, affording a transient intermediate sulfonium ylide that would be protonated under the reaction conditions to give adduct **6**. Addition of a suitable nucleophile **7** to this reaction mixture would result in the nucleophilic displacement of the sulfonium group within **6**, to afford desired products **4**.

We commenced our investigation by exposing an acetonitrile mixture of model carboxylic acid *N*-Boc-isonipecotic acid **10a**, vinyl systems **8**–**9**, potassium phosphate, and photocatalyst 1,2,3,5-tetrakis(carbazol-9-yl)-4,6-dicyanobenzene (4CzIPN)^25^ to blue light irradiation, followed by the addition of 2-mercaptoethanol as a model nucleophile under mild heating (60 °C), Table 1.

**Table 1:**
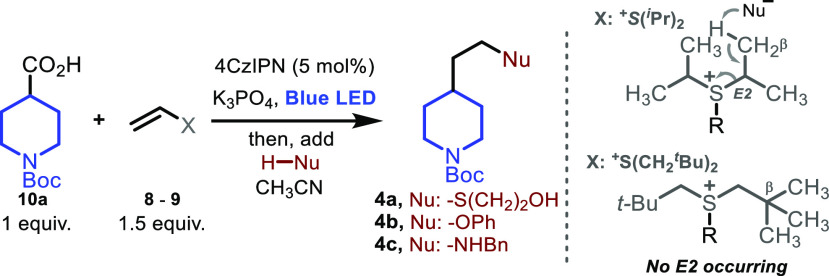
Optimization Studies

entry^a^**^,^**^b^	alkene (-X)	**4a** (%)	**4b** (%)	**4c** (%)
1	Br (**8**)	0	–	–
2	^+^S(Ph)_2_[OTf]^−^ (**9a**)	35	–	–
3^c^	^+^S(Ph)_2_[OTf]^−^ (**9a**)	49	–	–
4	^+^S(^*i*^Pr)_2_[OTf]^−^ (**9b**)	90	traces	traces
5^d^	^+^S(CH_2_^*t*^Bu)_2_[OTf]^−^ (**9c**)	71	62	65
6^e^	^+^S(CH_2_^*t*^Bu)_2_[OTf]^−^ (**9c**)	traces	–	–
7^f^	^+^S(CH_2_^*t*^Bu)_2_[OTf]^−^ (**9c**)	traces	–	–

a Reactions were performed in 0.05 mmol scale, using **10a** (1.0 equiv), **8**–**9** (1.5 equiv), 4CzIPN (5 mol %), and nucleophiles (2.5 equiv); see Supporting Information for full optimization details.

b Unless otherwise stated, ^1^H NMR yield using CH_2_Br_2_ as internal standard.

c 3CzClIPN (5 mol %) used as photocatalyst.

d Yields of isolated material in 0.2 mmol scale reactions.

e Reaction carried out in the presence of 1 equiv of TEMPO.

f No irradiation.

As expected, upon subjecting vinyl bromide **8** to the reaction conditions, polymerization was observed with no formation of desired compound **4a** (Table 1, entry 1). Interestingly, the use of diphenyl vinyl sulfonium triflate **9a**(26) led to the formation of desired product **4a** in a moderate yield of 35% (entry 2). ^1^H NMR analysis of the reaction mixture revealed poor mass balance and a complex reaction mixture, due to the high reactivity of the diaryl sulfonium system and the known tendency of this moiety to undergo reductive cleavage.^27^ The use of a photocatalyst with weaker reductive power (3CzClIPN)^28^ only slightly improved the results (entry 3). We speculated that the decoration of the sulfonium moiety with bulky alkyl lateral chains would improve its stability, thereby enhancing the efficiency of the process. In consonance with this hypothesis, upon subjecting di-isopropyl vinyl sulfonium **9b**(26) to our reaction conditions, the desired product **4a** was obtained in 90% yield (entry 4). However, the generality of this system was limited, as only traces of products **4b** and **4c** were detected when the corresponding nucleophiles were employed, presumably due to a dominant undesired E2 elimination occurring in the sulfonium intermediate (see Table 1, top right).^26^ Thus, in order to expand the applicability of our system and minimize the undesired side-reactivity, we designed novel vinyl sulfonium ion **9c**, equipped with bulky *neo*-pentyl alkyl lateral chains lacking β-protons. This novel reagent is a bench-stable solid, which can be stored in a standard freezer (−20 °C) for over 6 months with no detectable decomposition, and can be synthesized in high yield and multigram scale from commercial and affordable reagents, without any chromatographic purification needed (see Supporting Information for details). To our delight, this sulfonium salt ensured high generality to the process, with compounds **4a**–**c** respectively isolated in 71%, 62%, and 65% yield (entry 5). In consonance with the radical nature of the process, performing the reaction in the presence of 2,2,6,6-tetramethyl-1-piperidinyloxyl free radical (TEMPO), or in the absence of irradiation, led to complete inhibition of the reactivity (entries 6 and 7).

With the optimized conditions in hand, we next looked at exploring the generality of our polarity transduction strategy. Thus, model radical precursor **10a** was subjected to the reaction conditions, while varying the nature of the nucleophiles (Scheme 2). Thiol **4d** was obtained in 61% yield from commercial sodium hydrosulfide. Sulfides **4a**, **4e**, and **4f** were obtained in good to excellent yields respectively from primary, secondary, and aromatic thiols. The hypertension medicament captopril, carrying an additional carboxylic acid functionality, was conveniently converted into novel derivative **4g** in 53% yield upon stoichiometric dianion generation with sodium hydride, followed by addition to the sulfonium mixture in DMF (see Supporting Information for more details). For this entry, as well as for the other complex nucleophiles used to access products **4k**, **4l**, and **4p**, the stoichiometry of the system was adjusted to ensure the use of the valuable nucleophile as the limiting reagent (see Supporting Information). Alcohol **4h** was accessed in 52% yield employing a solution of water/HMPA and potassium bicarbonate to promote solvolysis of the corresponding sulfonium intermediate. Primary and secondary ethers **4i** and **4j** were accessed in moderate yields using aliphatic alcohols as nucleophiles. For these two entries vinyl diphenyl sulfonium triflate was used, potassium *tert*-butoxide was employed as a base, and the less reductive photocatalyst 3CzClIPN^28^ was used (see Supporting Information for details). Aromatic ether **4b** was obtained in 62% yield from phenol following standard reaction conditions. Unmodified alkaloid morphine was selectively converted into novel derivative **4k** in 78% yield, without side reactivity arising from the nucleophilic allylic alcohol and the tertiary amine present in the complex scaffold. Ester derivative **4l** was obtained in 82% yield from unmodified d-biotin, suggesting the possible future application of this chemistry in biotinylation strategies. Primary amine **4m** was obtained in 79% yield using commercially available ammonia in methanol solution as nucleophilic nitrogen source. Secondary, tertiary, and aromatic amines **4c**, **4n**, and **4o** were also obtained in good yields from the corresponding amine nucleophiles. The decongestant pseudoephedrine, bearing an amine and a neighboring alcohol functionality, underwent selective *N*-functionalization to provide novel derivative **4p** in 59% yield.

**Scheme 2:**
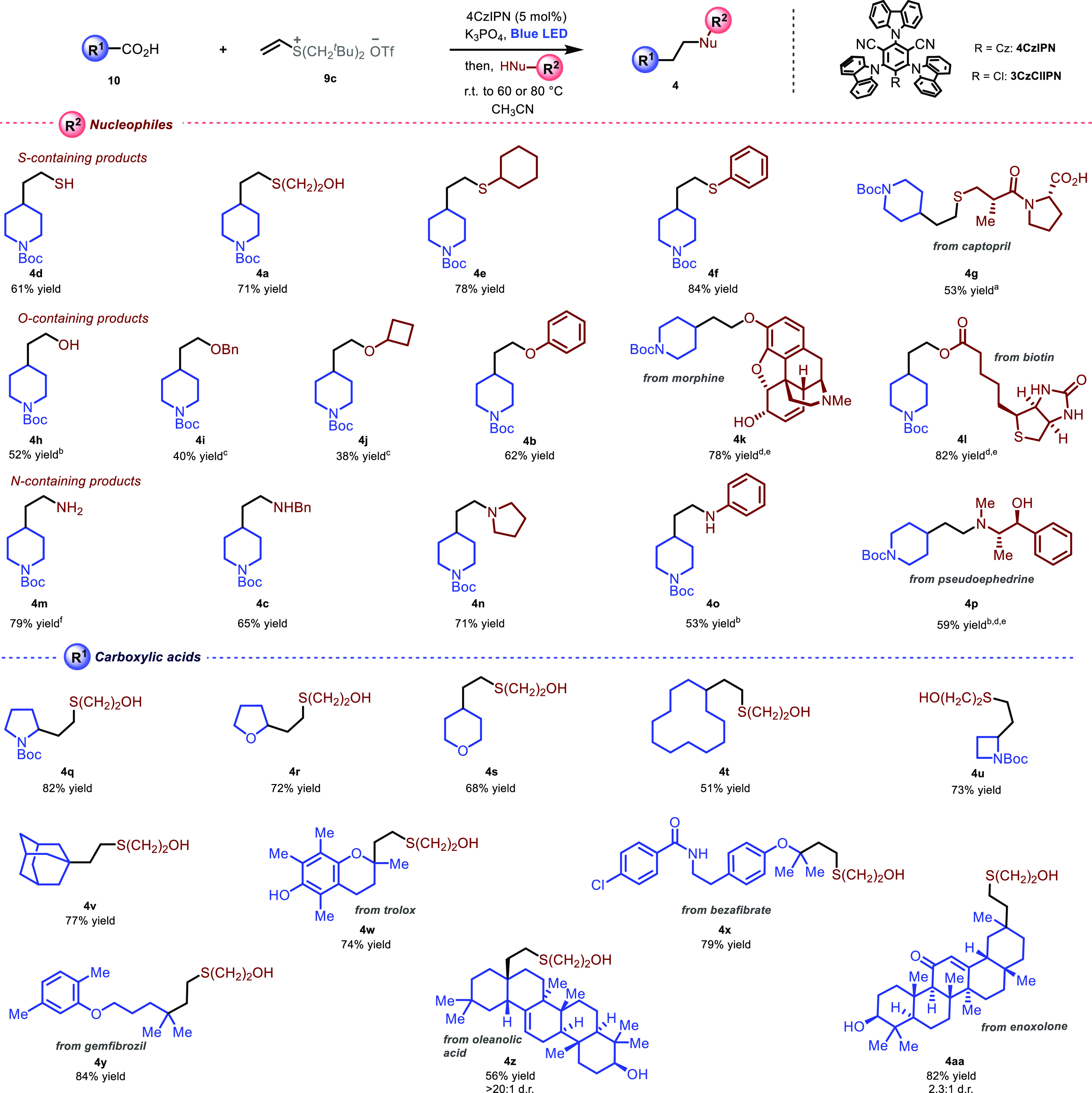
Reaction Scope Preformed captopril dianion was used as limiting reagent (see Supporting Information for full details). After nucleophile addition, the reaction was sealed and heated to 120 °C. Diphenyl vinyl sulfonium triflate **9a**, KO*t*Bu, catalytic 3CzClIPN were used and irradiation was performed at 0 °C. Reaction stoichiometry: **10a** (2.0 equiv), **9c** (2.2 equiv), 4CzIPN (10 mol %), and nucleophile (1.0 equiv). 0.1 mmol scale. After nucleophile addition, the reaction was sealed and heated to 100 °C. Reactions were performed in a 0.2 mmol scale, using **10** (1 equiv), **9c** (1.5 equiv), 4CzIPN (5 mol %), and nucleophile (typically 2.5 equiv), in CH_3_CN unless otherwise stated; see Supporting Information for full experimental details.Cz: carbazolyl.

We next looked at exploring the generality of our strategy by varying the nature of the carboxylic acid radical precursor. A variety of *N*- or *O*-containing heterocyclic carboxylic acids underwent the desired reactivity to afford compounds **4q**–**4s** in good to excellent yields. Cyclic molecules with different ring size were successfully functionalized, with both macrocycle **4t** as well as strained four-membered ring **4u** obtained respectively in 51% and 73% yield from the corresponding carboxylic acids. Compound **4v** was obtained in 77% yield from bulky adamantane carboxylic acid.

We next tested the methodology in the functionalization of structurally complex carboxylic acids. Compound **4w** was obtained in 74% yield from vitamin E analogue Trolox, with no observable side reactivity arising from the free phenolic group. Functionalization of the hyperlipidemia treatment drugs bezafibrate and gemfibrozil afforded novel derivatives **4x** and **4y** in 79% and 84% yield. Finally, derivatization of complex triterpenoid structures bearing multiple functionalities, e.g., free alcohols, a carbonyl, and an activated alkene moiety, led to desired compound **4z** and **4aa** in respectively 56% and 82% yield.

Delighted by the wide scope of application of this methodology, we questioned whether the use of bromo-sulfonium structure **13** (Scheme 3), a synthetic intermediate in-route to vinyl sulfonium **9c**, would undergo the desired reactivity via *in situ* generation of the corresponding vinyl system, further streamlining the chemistry. Pleasingly, upon subjecting **13** to our reaction conditions, final compound **4a** was isolated in 67% yield, confirming the feasibility of this approach.

**Scheme 3:**
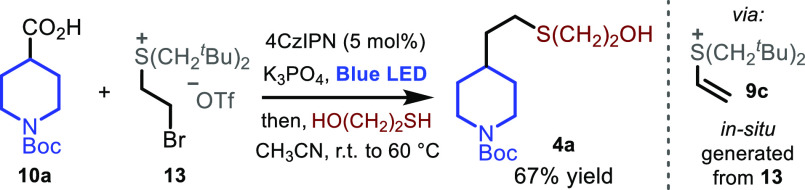
*In Situ* Generation of the Vinyl Sulfonium Reaction performed in a 0.2 mmol scale, using **10a** (1 equiv), **13** (1.5 equiv), 4CzIPN (5 mol %) and mercaptoethanol (2.5 equiv); see Supporting Information for details.

By allowing practical access to products that would be the result of the forbidden reaction between nucleophilic alkyl radicals and electron-rich double bonds, this methodology formally redefines the scope of the classic addition of carbon-centered radicals to double bonds. We anticipate that this novel reactivity platform will stimulate considerable advances in various areas of synthesis.

## Supporting Information Available

Spectral data for all compounds, additional experimental details, materials, methods, including photographs of experimental setup (PDF)
