# Supplementary material for: Polarity Transduction Enables the Formal Electronically Mismatched Radical Addition to Alkenes
Source: J Am Chem Soc. 2023 Jan 31;145(5):2773–8. doi: 10.1021/jacs.2c12699 (PMC9912259; doi:10.1021/jacs.2c12699)

Supporting Information for

Polarity Transduction Enables the Formal Electronically Mismatched Radical Addition to Alkenes

Subhasis Paul, Dario Filippini and Mattia Silvi

School of Chemistry, University of Nottingham,
Nottingham, NG7 2RD, United Kingdom
Correspondence to: mattia.silvi@nottingham.ac.uk

Table of Contents

1. General experimental S2
   1.1. Solvents, reagents, and starting materials S2
   1.2. Chromatography and instrumental analysis S2
   1.3. Naming of compounds S3
   1.4. LEDs S3
2. Synthesis of the photocatalysts and novel starting materials S4
   2.1. Synthesis of 4CzIPN S4
   2.2. Synthesis of 3CzClIPN S5
   2.3. Synthesis of dineopentyl(vinyl)sulfonium triflate (9c) S6
3. Optimization studies S9
   3.1. Effect of the sulfonium structure S9
   3.2. Effect of standard reaction parameters S10
   3.3. Study of reaction conditions for S_N2 with different nucleophiles S10
4. General procedures & product characterization S14
   4.1. General procedure A S14
   4.2. General procedure B S14
   4.3. General procedure C S15
   4.4. General procedure D S16
   4.5. Reaction set-up for irradiation of mixtures with blue LEDs S17
   4.6. Reaction products and characterization S19
   4.7. Reaction with *in-situ* generation of the vinyl sulfonium S37
5. References S38
6. NMR Spectra S39

1. General experimental

1.1. Solvents, reagents, and starting materials

All air and water-sensitive reactions were carried out in oven-dried glassware under argon atmosphere using standard Schlenk manifold technique. The solvents were degassed when needed by bubbling argon for ten minutes. Bulk solutions were evaporated under reduced pressure using a Büchi rotary evaporator. All solvents were commercially supplied or provided by the communal stills of the School of Chemistry, University of Nottingham. Commercially available compounds were purchased from Sigma Aldrich, Alfa Aesar, Acros, Fluorochem, TCI chemicals and used as received. Dry solvents were purchased from Acros Organic, Extra Dry over molecular sieves, AcroSeal®.

Compounds **10a**,[1] **10f**,[2] diisopropyl(vinyl)sulfonium triflate **9b**[3] and diphenyl(vinyl)sulfonium triflate **9a**[4] were synthesized following reported procedures.

1.2. Chromatography and instrumental analysis

Flash column chromatography (FCC) was carried out using Sigma-Aldrich silica gel LC60A-40 (63 µm). All reactions were followed by thin-layer chromatography (TLC) when practical, using Merck Kieselgel 60 F_{254} fluorescent treated silica which was visualised under UV light, by staining with aqueous basic potassium permanganate, phosphomolybdic acid or with ninhydrin solutions.

^{1}H-NMR, ^{13}C-NMR and **^{19}F-NMR** spectra were recorded using Bruker broadband prodigy cryoprobe AV(III)500HD 500 MHz and Bruker AV(III)400HD 400 MHz spectrometers. Chemical shifts (δ) are given in parts per million (ppm) and coupling constants (*J*) are given in hertz (Hz). The ^{1}H-NMR spectra are reported as follows: ppm (multiplicity, coupling constants, number of protons). High resolution mass spectra (**HRMS**) were recorded on a Bruker MicrOTOF II by Electrospray Ionization (ESI). **IR** spectra were recorded on a Bruker Vertex 70 FT-IR ATR as a thin film. Only selected absorption maxima (ν_{max}) are reported in wavenumbers (cm^{-1}). **Melting points** were recorded in degrees Celsius (°C), using a Stuart melting point SMP 20 microscope apparatus and are reported uncorrected. Optical rotation (**$[\alpha]_D^{25}$**) was recorded on an Anton Paar MCP 100 at 25 °C, in chloroform, with a concentration of 1g/100mL.

1.3. Naming of compounds

Compound names are generated by ChemDraw 20.0 software (PerkinElmer), following the IUPAC nomenclature.

1.4. LEDs

Irradiation of reaction mixtures was performed using Kessil lamp A160WE Tuna Blue Saltwater LED Light 40W set to blue at maximum intensity. Lamp emission is observed to decay over long usage due to LED chip deterioration. To ensure reproducibility of the results, we recommend using new lamps. If needed, full conversion of the photochemical reactions detailed in section 4.6 can be ensured *via* ^{1}H-NMR analysis of a minimal reaction aliquot after irradiation, monitoring disappearance of the characteristic reagent vinyl signals at 6.38 – 6.32 ppm.

2. Synthesis of the photocatalysts and novel starting materials

2.1. Synthesis of 4CzIPN

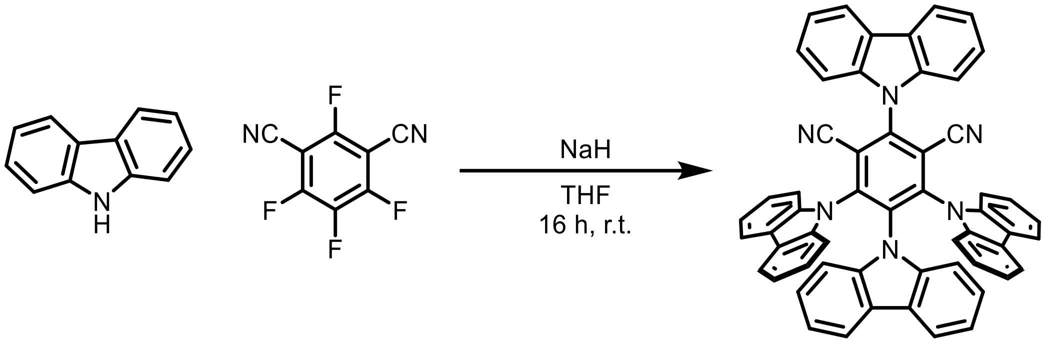

4CzIPN was synthesised through a modification of a known procedure.[5]

NaH (60% in oil; 15.0 mmol; 600 mg) was slowly added to a stirred solution of 9-carbazole (10.0 mmol; 1.67 g) in dry THF (40 mL) under argon atmosphere at room temperature. After 30 min, tetrafluoroisophthalonitrile (2.00 mmol; 400 mg) was added. After stirring at room temperature for 16 h, 200 µL of distilled water were added to the mixture to quench the excess NaH. Then the solvent was evaporated under reduced pressure. The yellow powder was solubilized in DCM and celite was added. The solvent was removed again under reduced pressure and the resulting powder was dry-loaded and purified through column chromatography (SiO_2; gradient 75:25 to 1:1 hexane:DCM) to afford 4CzIPN (1.2 g; 76%) as a bright yellow fine powder. **R_f** (1:1 hexane:DCM) 0.6; **^{1}H NMR** ($CDCl_3$, 500 MHz) δ (ppm): 8.23 (d, *J* = 7.7 Hz, 2H), 7.81 – 7.62 (m, 8H), 7.49 (ddd, *J* = 8.0, 6.8, 1.5 Hz, 2H), 7.33 (d, *J* = 7.7 Hz, 2H), 7.23 (dd, *J* = 6.8, 2.1 Hz, 4H), 7.12 – 7.05 (m, 8H), 6.89 – 6.78 (m, 4H), 6.64 (t, *J* = 7.8 Hz, 2H); **^{13}C NMR** ($CDCl_3$, 126 MHz) δ (ppm): 145.4, 144.8, 140.1, 138.3, 137.1, 134.9, 127.1, 125.9, 125.1, 124.9, 124.7, 124.0, 122.6, 122.1, 121.6, 121.1, 120.6, 119.8, 116.5, 111.8, 110.1, 109.6, 109.6.

The spectra match the ones reported.[6]

2.2. Synthesis of 3CzClIPN

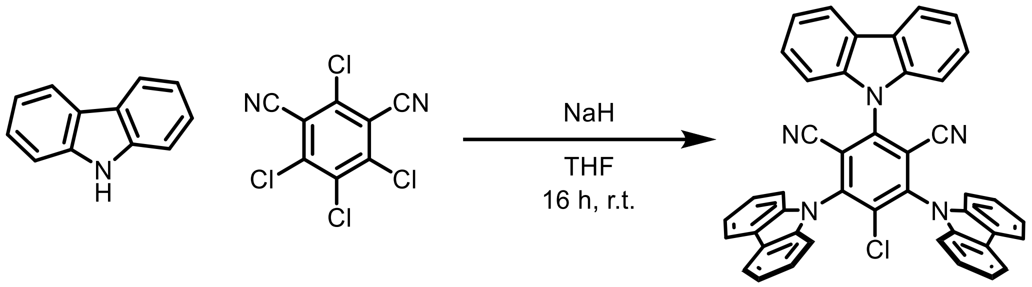

3CzClIPN was synthesised through a modification of a known procedure.[7]

NaH (60% in oil; 4.3 mmol; 103 mg) was slowly added to a stirred solution of 9-carbazole (2.9 mmol; 477 mg) in dry THF (15 mL) under argon atmosphere at room temperature. After 30 min, tetrachloroisophthalonitrile (0.8 mmol; 202 mg) was added. After stirring at room temperature for 16 h, 80 µL of distilled water were added to the mixture to quench the excess NaH. Then the solvent was evaporated under reduced pressure. The yellow powder was solubilized in DCM and celite was added. The solvent was removed again under reduced pressure and the resulting powder was dry-loaded and purified through column chromatography (SiO_2; gradient 6:4 to 4:6 hexane:DCM) to afford 3CzClIPN (314 mg; 63%) as a bright yellow fine powder. **^{1}H NMR** ($CDCl_3$, 500 MHz) δ (ppm): 8.18 (d, *J* = 7.7 Hz, 6H), 7.64 – 7.53 (m, 6H), 7.50 – 7.35 (m, 8H), 7.28 (d, *J* = 8.2 Hz, 4H); **^{13}C NMR** ($CDCl_3$, 126 MHz) δ (ppm): 145.2, 144.4, 139.9, 139.4, 137.2, 127.1, 127.0, 124.9, 124.7, 122.6, 122.3, 121.5, 121.4, 117.7, 110.8, 109.6, 109.4.

The spectra match the ones reported.[7]

2.3. Synthesis of dineopentyl(vinyl)sulfonium triflate (9c)

Dineopentyl(vinyl)sulfonium triflate (**9c**) was synthesized as reported in Scheme S1.

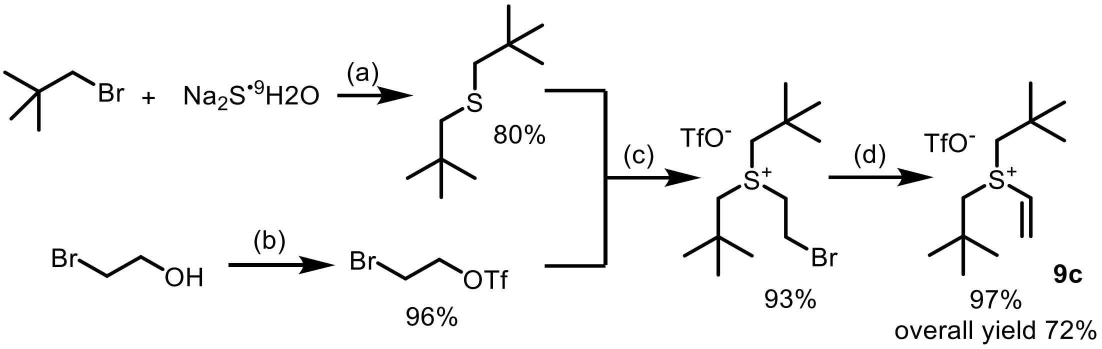

Scheme S1: Synthetic route for dineopentyl(vinyl)sulfonium triflate (a) tributylhexadecylphosphonium bromide (10 mol%), H_2O, 80 °C; (b) Tf_2O, Py, DCM, -20 °C to r.t.; (c) DCM, r.t.; (d) $KHCO_3$, THF:H_2O (2:1), r.t.

Synthesis of dineopentyl sulfide

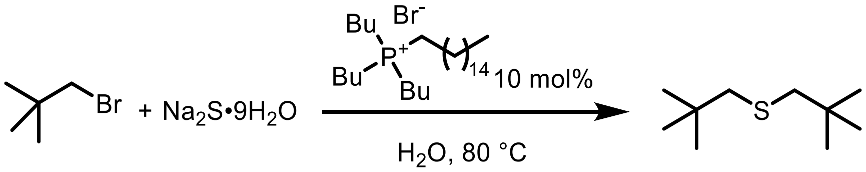

Dineopentyl sulfide was synthesised through a modification of a known procedure.[8]

$Na_2S{\cdot}9H_2O$ (0.7 equiv.; 70 mmol; 16.8 g) and tributylhexadecylphosphonium bromide (0.1 equiv.; 10 mmol; 5.1 g) were loaded in a 100 mL round bottom flask equipped with a reflux condenser and dissolved in 30 mL of degassed water (degassed by argon sparging for 20 min). Neopentyl bromide (1.0 equiv.; 100 mmol; 12.6 mL) was added and the mixture was heated up to 80 °C for 24 h under vigorous stirring. The mixture was cooled down to room temperature and the organic layer was separated and vacuum distilled (108 °C at 50 mbar). To further remove traces of water, after the distillation the compound was passed through a Pasteur filled with anhydrous $MgSO_4$ to obtain the product (7.0 g; 80%) as a transparent oil.

^{1}H NMR ($CDCl_3$, 500 MHz) δ (ppm): 2.45 (s, 4H), 0.98 (s, 18H); **^{13}C NMR** ($CDCl_3$, 126 MHz) δ (ppm): 50.1, 32.8, 29.1.

The spectra match the ones reported.[9]

Synthesis of 2-bromoethyl trifluoromethanesulfonate

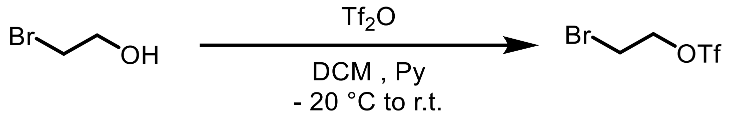

2-bromoethyl trifluoromethanesulfonate was synthesised following a known literature procedure.[4]

Trifluoromethanesulfonic anhydride (1.04 equiv.; 83 mmol; 14 mL) was dropwise added to a stirred solution of pyridine (1.08 equiv.; 86 mmol; 7.0 mL) in dry DCM (76 mL) at -20 °C under argon atmosphere and the stirring was continued for 10 minutes. Then 2-bromoethanol (1.00 equiv.; 80 mmol; 5.7 mL) was slowly added to this cold reaction mixture. The cooling bath was removed, and the mixture was left warming up to room temperature while stirring for 10 minutes (strictly following this time was found to be important). The resulting suspension was filtered, concentrated (using a rotary evaporator, maintaining the water bath below 20 °C) and petroleum ether (50 mL) was added. The mixture was again filtered and concentrated under reduced pressure and finally dried under high vacuum to give the product (19.7 g; 96%) as a light brown oil which was used for the next step without further purification.

^{1}H NMR ($CDCl_3$, 400 MHz) δ (ppm): δ 4.74 (t, *J* = 6.4 Hz, 2H), 3.61 (t, *J* = 6.4 Hz, 2H); **^{19}F NMR** ($CDCl_3$, 376 MHz) δ (ppm): -74.5 (s, 3F); **^{13}C NMR** ($CDCl_3$, 101 MHz) δ (ppm): 74.4, 26.3.

The spectra match the ones reported.[4]

Synthesis of (2-bromoethyl)dineopentylsulfonium triflate (13)

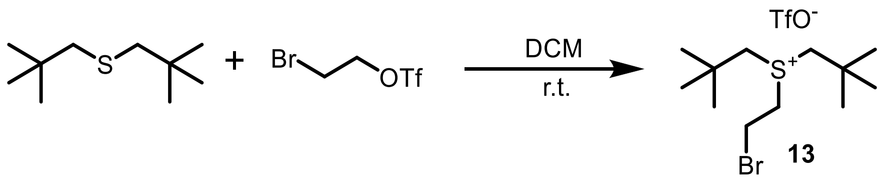

Dineopentyl sulfide (1.0 equiv.; 40 mmol; 6.97 g) was dissolved in dry DCM (40 mL). 2-bromoethyl trifluoromethanesulfonate (1.1 equiv.; 42 mmol; 10.8 g) was added and the reaction was left stirring for 4 days at room temperature monitoring the conversion *via* ^{1}H-NMR analysis of a minimal reaction aliquot. The solvent was removed by rotary evaporation and the white solid was washed three times with 10 mL of hexane to give product **13** (16.1 g; 93%) as a white solid, which was used for the next step without further purifications.

IR (film) ν_{max}/cm^{-1}:2965, 2876, 1476, 1400, 1372, 1255, 1157, 1030, 907, 755, 705, 637, 572, 518; **M.P.** = 97 °C; **^{1}H NMR** ($CDCl_3$, 500 MHz) δ (ppm): δ 4.24 – 4.16 (m, 2H), 3.80 – 3.73 (m, 2H), 3.68 (d, *J* = 14.4 Hz, 2H), 3.47 (d, *J* = 14.4 Hz, 2H), 1.19 (s, 18H); **^{19}F NMR** ($CDCl_3$,

376 MHz) δ (ppm): -78.4 (s, 3F); **^{13}C NMR** ($CDCl_3$, 126 MHz) δ (ppm): 58.1, 49.0, 32.9, 29.0, 24.9.**HRMS** (ESI-TOF) mass calculated for $[M]^+$ ($C_{12}H_{26}BrS^+$) expected *m/z* 281.0933; found *m/z* 281.0929.

<u>Synthesis of dineopentyl(vinyl)sulfonium triflate (9c)</u>

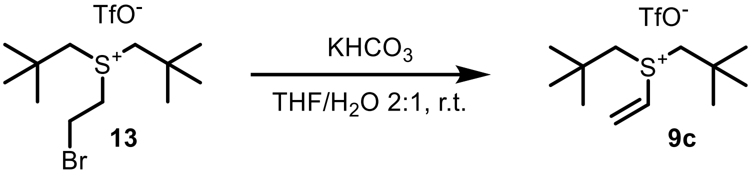

(2-bromoethyl)dineopentylsulfonium triflate **13** (1.0 equiv.; 37 mmol; 16.1 g) was dissolved in 75 mL of a 2:1 mixture of THF and water. $KHCO_3$ (1.5 equiv.; 55 mmol; 5.54 g) was added and the reaction was left stirring for 48 h at room temperature monitoring the conversion *via* ^{1}H-NMR analysis of a minimal reaction aliquot. THF was removed under reduced pressure and the reaction mixture was extracted in DCM (100 mL x3), the organic phases collected, dried over anhydrous $MgSO_4$ and the solvent was removed through rotary evaporation to give compound **9c** (12.6 g; 97%) as a white solid (see Fig. S1), which does not require further purifications. **IR** (film) ν_{max}/cm^{-1}: 3046, 2966, 2876, 1602, 1482, 1373, 1255, 1224, 1154, 1033, 639; **M.P.** = 139 °C; **^{1}H NMR** ($CDCl_3$, 500 MHz) δ (ppm): δ 7.44 – 7.35 (m, 1H), 6.41 – 6.31 (m, 2H), 3.87 (d, *J* = 14.0 Hz, 2H), 3.43 (d, *J* = 14.0 Hz, 2H), 1.16 (s, 18H); **^{1}H NMR** (DMSO-d_6, 500 MHz) δ (ppm): 7.01 (dd, *J* = 16.3, 8.8 Hz, 1H), 6.81 (dd, *J* = 16.3, 1.8 Hz, 1H), 6.54 (dd, *J* = 8.8, 1.8 Hz, 1H), 3.65 (d, *J* = 13.3 Hz, 2H), 3.47 (d, *J* = 13.3 Hz, 2H), 1.08 (s, 18H); **^{19}F NMR** ($CDCl_3$, 376 MHz) δ (ppm): -78.4 (s, 3F); **^{13}C NMR** ($CDCl_3$, 126 MHz) δ (ppm): 135.5, 128.0, 60.5, 33.1, 29.0. **HRMS** (ESI-TOF) mass calculated for $[M]^+$ ($C_{12}H_{25}S^+$) expected *m/z* 201.1672; found *m/z* 201.1670.

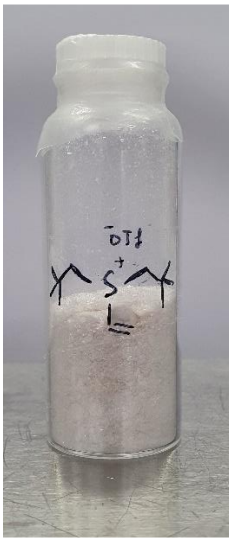

Fig. S1: compound **9c**

<u>Synthesis of the model isopentyldineopentylsulfonium triflate (S2) for optimization studies</u>

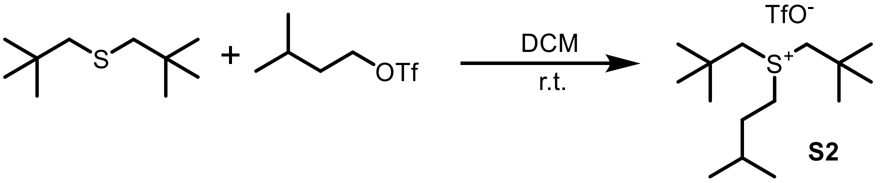

Dineopentyl sulfide (1.0 equiv.; 7.3 mmol; 1.27 g) was dissolved in dry DCM (7.3 mL). Isopentyl trifluoromethanesulfonate (1.01 equiv.; 7.4 mmol; 1.62 g) was added and the reaction

was left stirring for 24 h at room temperature monitoring the conversion *via* ^{1}H-NMR analysis of a minimal reaction aliquot. The solvent was removed by rotary evaporation and the white solid was washed three times with 5 mL of hexane to give the product **S2** (2.76 g; 96%) as a white solid, which was used without further purifications.

IR (film) ν_{max}/cm^{-1}:2960, 2827, 1470, 1370, 1270, 1251, 1247, 1223, 1154, 1027, 636, 573, 516; **M.P.** = 83 °C; **^{1}H NMR** ($CDCl_3$, 500 MHz) δ (ppm): δ 3.60 – 3.52 (m, 4H), 3.48 (d, *J* = 14.4 Hz, 2H), 1.77 (hept, *J* = 6.7 Hz, 1H), 1.68 – 1.61 (m, 2H), 1.16 (s, 18H), 1.00 (d, *J* = 6.7 Hz, 6H); **^{19}F NMR** ($CDCl_3$, 376 MHz) δ (ppm): -78.4 (s, 3F); **^{13}C NMR** ($CDCl_3$, 126 MHz) δ (ppm): 58.0, 43.4, 34.0, 32.6, 29.0, 27.5, 22.1;**HRMS** (ESI-TOF) mass calculated for $[M]^+$ ($C_{15}H_{33}S^+$) expected *m/z* 245.2297; found *m/z* 245.2303.

3. Optimization studies

3.1. Effect of the sulfonium structure

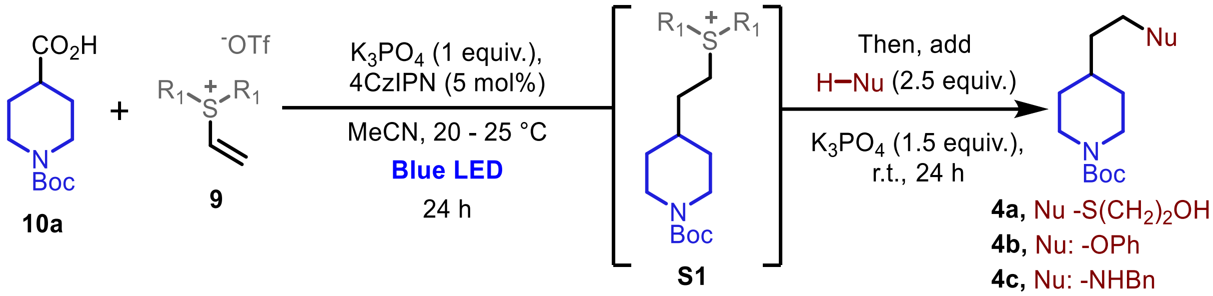

Entry[a]	R_1	S1 formed (%)[b]	Yield 4a (%)[b]	Yield 4b (%)[b]	Yield 4c (%)[b]
1	Ph	42	35	-	-
2[c]	Ph	59	49	-	-
3[d]	^{i}Pr	92	90	Traces	Traces
4[d,e]	$CH_2{}^tBu$	86	77 (71)	64 (62)	70 (65)[f]

[a] Reactions performed in 0.05 mmol scale, using **10a** (1.0 equiv.), **9** (1.5 equiv.), $[\mathbf{10a}]_0$ = 0.15M. Nucleophiles addition was performed following the general procedures A, B and C as reported in section 4; [b] Unless otherwise stated, ^{1}H-NMR yield using CH_2Br_2 as internal standard; [c] 3CzClIPN used in place of 4CzIPN, see section 2.2 for catalyst structure; [d] Unless otherwise stated, second step performed at 60 °C; [e] reaction scale 0.2 mmol, isolated yield in parenthesis. [f] Second step performed at 80 °C

3.2. Effect of standard reaction parameters

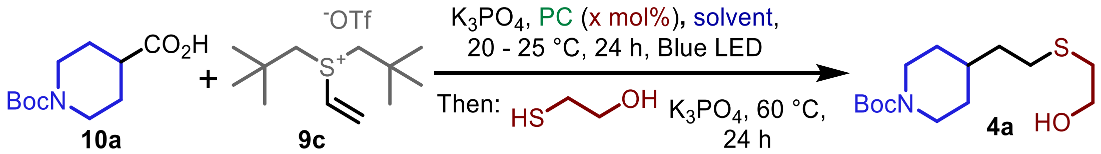

Entry[a]	PC	PC loading (mol%)	Solvent	Yield 4a (%)[b]
1	4CzIPN	5	DMF	59
2	4CzIPN	5	DMSO	63
3	4CzIPN	5	THF	49
4[c]	4CzIPN	5	MeCN	77
5	4CzIPN	2.5	MeCN	70
6	4CzIPN	1	MeCN	66
7	$Ru(bpy)_3(PF_6)_2$	1	MeCN	0
8	$[Ir(dtbbpy)(ppy)_2]PF_6$	1	MeCN	3
9	$(Ir[dF(CF_3)ppy]_2(dtbpy))PF_6$	1	MeCN	19

[a] Reactions performed in 0.05 mmol scale, using **10a** (1.0 equiv.), **9c** (1.5 equiv.) and K_3PO_4 (1 equiv.), $[\mathbf{10a}]_0$ = 0.15M; then 2-mercaptoethanol (2.5 equiv.) and K_3PO_4 (1.5 equiv.) were added; [b] ^{1}H-NMR yield using CH_2Br_2 as internal standard; [c] Reaction scale 0.2 mmol

3.3. Study of reaction conditions for S_N2 with different nucleophiles

As we found that different nucleophiles undergo S_N2 reactivity with the intermediate sulfoniums under different conditions, optimization studies of the standard reaction parameters were performed. For practicality, the optimizations were performed using sulfonium **S2**, used as a model system for intermediate **S1**. When possible, the same solvent system and base used in the photochemical step were maintained, in order to allow to practically perform the full process simply adding reagents to the mixture without any manipulation.

While final yields of model systems do not necessarily reflect the yields obtained in the full photochemical process, the model reactions below have been valuable to identify suitable reaction conditions for the reactions presented in this report.

3.3.1. Aromatic alcohols

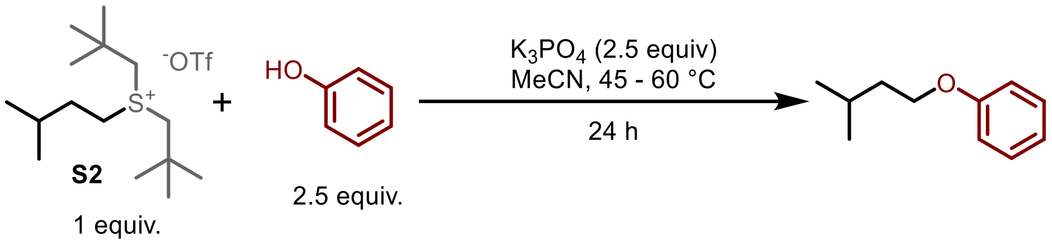

**Entry**[a]	**[S2]₀** (M)	**Temp.** (°C)	**Yield** (%)[b]	**SMR** (%)[c]
1	0.15	60	69	5
2	0.5	60	82	0
3	1.0	60	80	0
4	0.5	45	42	57

[a] Reactions performed in 0.05 mmol scale in a 2 mL vial.; [b] ^{1}H-NMR yield using CH_2Br_2 as internal standard; [c] Starting material recovery of the model sulfonium **S2** established by ^{1}H-NMR analysis using CH_2Br_2 as internal standard.

3.3.2. Aliphatic alcohols

As with this specific class of nucleophiles the results obtained with the model system **S2** were found to significantly differ from the full photochemical process, the optimization study was performed carrying out the full photochemical reaction, as presented below.

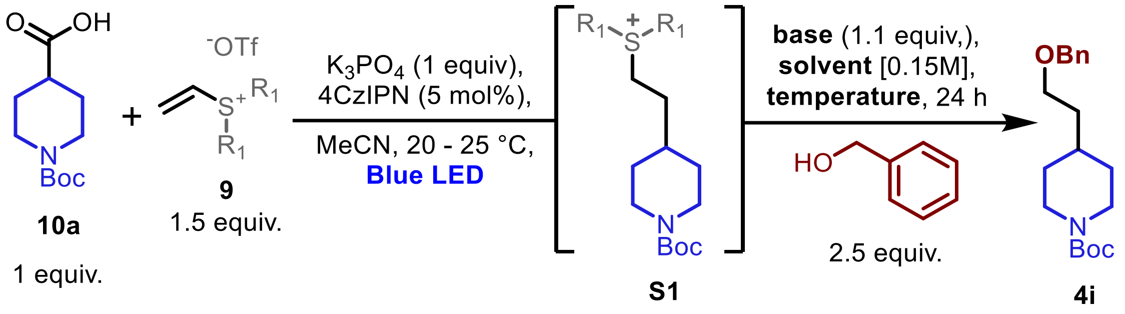

**Entry**[a]	**R₁**	**Temp.** (°C)	**Solvent**	**Base**	**Yield 4i** (%)[b]
1	$CH_2{}^t$Bu	60	MeCN	K_3PO_4[c]	0
2	$CH_2{}^t$Bu	r.t.	MeCN	^{t}BuOK[c]	10
3	$CH_2{}^t$Bu	60	MeCN	^{t}BuOK	0
4	Ph[d,e]	0 to r.t.	MeCN	^{t}BuOK	31
5	Ph[d,e]	0 to r.t.	DMF[f]	^{t}BuOK	13
6	Ph[d,e]	0 to r.t.	DCM[f]	^{t}BuOK	47

[a] Reactions performed in 0.05 mmol scale using **10a** (1.0 equiv.), **9** (1.5 equiv.) and K_3PO_4 (1 equiv.), [**10a**]₀ = 0.15M; [b] ^{1}H-NMR yield using CH_2Br_2 as internal standard; [c] 2.5 equiv. of base was used; [d] 3CzClIPN (5 mol%) was used; [e] Photochemical step was performed at 0 °C; [f] *In-situ* solvent exchange using a standard Schlenk vacuum line

3.3.3. Amines

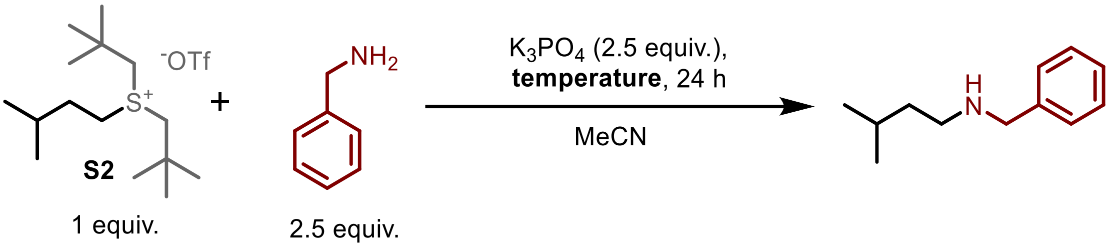

Entry[a]	Temp. (°C)	[S2]0 (M)	Yield (%)[b]	SMR (%)[c]
1	60	0.15	18	77
2	60	0.50	56	44
3	80	0.15	54	5
4	80	0.50	84	0

[a] Reactions performed in 0.05 mmol scale in a 2 mL vial.; [b] ^{1}H-NMR yield using CH_2Br_2 as internal standard; [c] starting material recovery of the model sulfonium **S2** established by ^{1}H-NMR analysis using CH_2Br_2 as internal standard.

3.3.4. Water

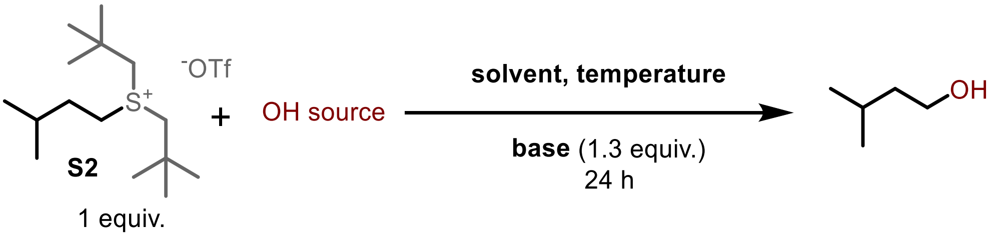

Entry[a]	OH source	Solvent	Temp. (°C)	Base	Yield (%)[b]	SMR (%)[c]
1	Bu_4NOH[d]	DCM (0.15M)	60	-	10	0
2[e]	H_2O[f]	DMPU (0.25M)	100	-	0	88
3[e]	H_2O[f]	HMPA (0.25M)	100	-	50	20
4[e]	H_2O[f]	HMPA (0.25M)	120	-	74	0
5[e]	H_2O[f]	HMPA (0.25M)	120	$KHCO_3$	87	0

[a] Reaction performed in a 0.05 mmol scale in a 2 mL Young tube; [b] ^{1}H-NMR yield using CH_2Br_2 as internal standard; [c] starting material recovery of the model sulfonium **S2** established by ^{1}H-NMR analysis using CH_2Br_2 as internal standard; [d] $Bu_4N^+OH^- \cdot 30H_2O$; [e] Condition inspired by a similar process performed on an alkyl halide, see reference 10 for details; [f] 45 equiv.

3.3.5. Ammonia

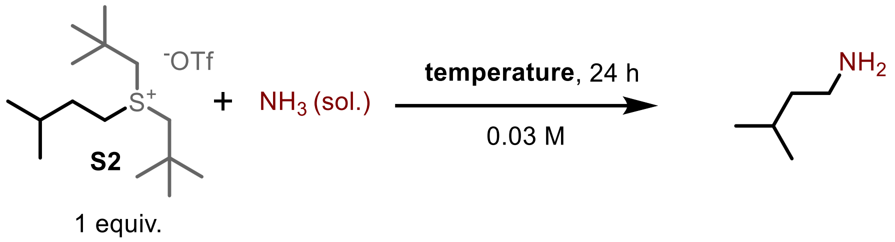

Entry[a]	NH_3 solution	Temp. (°C)	Yield (%)[b]
1	NH_3 in MeOH (7M)	100	94
2	NH_3 in MeOH (7M)	80	83
3	$NH_4OH_{(aq.)}$ (35%)	130	55
4	$NH_4OH_{(aq.)}$ (35%)	100	33

[a] Reactions performed in 0.05 mmol scale in a 2 mL microwave vial.; [b] ^{1}H-NMR yield using CH_2Br_2 as internal standard.

3.3.6. Hydrosulfide

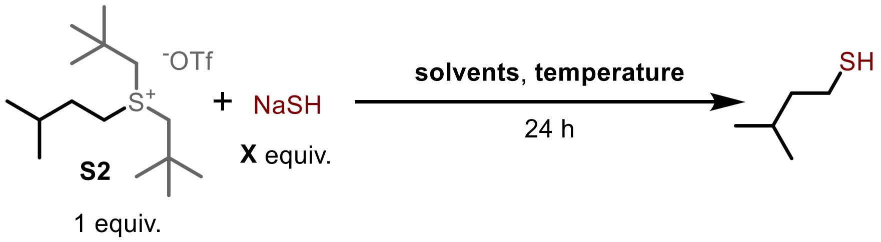

Entry[a]	Solvent	NaSH equiv.	Temp. (°C)	Yield (%)[b]	SMR (%)[c]
3	MeOH	2.50	60	53	0
4	MeOH	1.25	60	49	0
5[d]	MeCN	2.50	r.t.	66	0

[a] Reactions performed in 0.05 mmol scale in a 2 mL vial, $[\textbf{S2}]_0$ = 0.15M.; [b] ^{1}H-NMR yield using CH_2Br_2 as internal standard. The yields presented in this table may also include variable amounts of symmetric disulfide, difficult to estimate by NMR due to signal overlap. Disulfide formation can be completely suppressed using degassed solvents under Ar (see section 4.6 for details); [c] starting material recovery of the model sulfonium **S2** established by ^{1}H-NMR analysis using CH_2Br_2 as internal standard; [d] 30 mol % of tetrabutyl ammonium bromide was added to increase the solubility of the nucleophile.

4. General procedures & product characterization

4.1. General procedure A

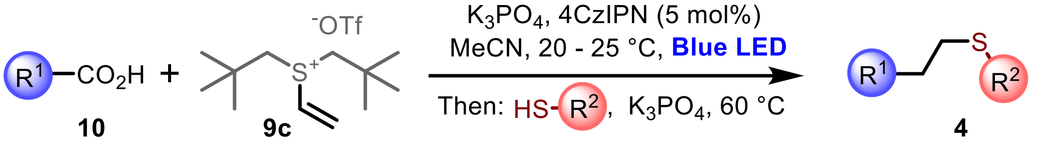

Carboxylic acid **10** (1.0 equiv.; 0.2 mmol), dineopentyl(vinyl)sulfonium triflate **9c** (1.5 equiv.; 0.3 mmol; 105 mg), 4CzIPN (0.05 equiv.; 0.01 mmol; 7.9 mg) and potassium phosphate (1.0 equiv.; 0.2 mmol; 42.4 mg) were introduced into a Schlenk tube. The atmosphere was exchanged to argon, and degassed dry MeCN (1.3 mL, 0.15 M, previously degassed through 10 min argon sparging) was introduced through a syringe. The vessel was sealed and placed in a glass-wall water bath with temperature controlled between 20 - 25 °C. The reaction was irradiated through the glass wall with blue light (Kessil lamp A160WE Tuna Blue Saltwater LED Light 40W) for 24 to 48 h under moderate stirring (500 rpm). The vessel was then removed from the water bath and the corresponding thiol (2.5 equiv.; 0.5 mmol) and further potassium phosphate (1.5 equiv.; 0.3 mmol; 63.6 mg) were introduced into the vessel. The mixture was heated to 60 °C without irradiation for 24 h under vigorous stirring (700 rpm). The crude was filtered through celite and eluted with DCM. Volatiles were evaporated under reduced pressure, and the residue was subjected to chromatography purification on silica gel to afford final compounds.

4.2. General procedure B

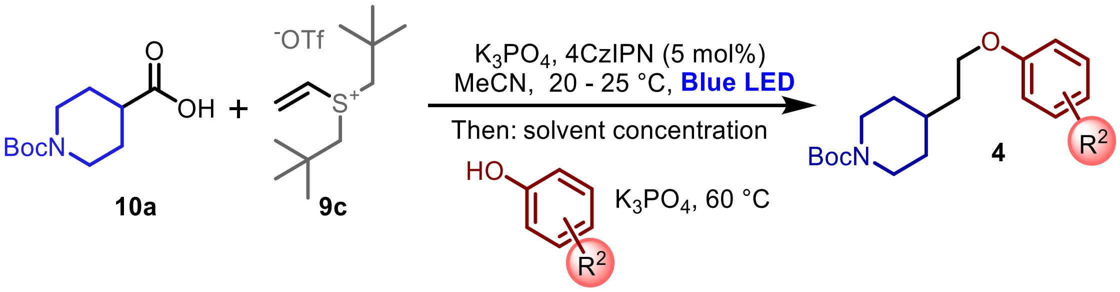

1-(*tert*-butoxycarbonyl)piperidine-4-carboxylic acid **10a** (1.0 equiv.; 0.2 mmol; 45.9 mg), dineopentyl(vinyl)sulfonium triflate **9c** (1.5 equiv.; 0.3 mmol; 105 mg), 4CzIPN (0.05 equiv.; 0.01 mmol; 7.9 mg) and potassium phosphate (1.0 equiv.; 0.2 mmol; 42.4 mg) were introduced into a Schlenk tube. The atmosphere was exchanged to argon, and degassed dry MeCN (1.3 mL, 0.15 M, previously degassed through 10 min argon sparging) was introduced through a syringe. The vessel was sealed and placed in a glass-wall water bath with temperature

controlled between 20 - 25 °C. The reaction was irradiated through the glass wall with blue light (Kessil lamp A160WE Tuna Blue Saltwater LED Light 40W) for 24 h under moderate stirring (500 rpm). The vessel was then removed from the water bath and the reaction mixture transferred in a 5 mL Young tube (washing the original vessel with DCM to ensure quantitative transfer). The solvent was removed blowing nitrogen. The corresponding aromatic alcohol (2.5 equiv.; 0.5 mmol), potassium phosphate (1.5 equiv.; 0.3 mmol; 63.6 mg) and dry acetonitrile (400 µL; 0.5 M) were added. The mixture was heated to 60 °C without irradiation for 24 h under vigorous stirring (700 rpm), after which it was filtered through celite and eluted with DCM. Volatiles were evaporated under reduced pressure, and the residue was subjected to chromatography purification on silica gel to afford final compounds.

4.3. General procedure C

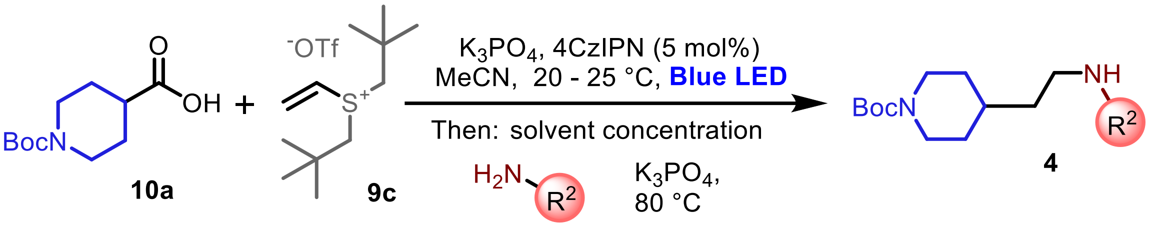

1-(*tert*-butoxycarbonyl)piperidine-4-carboxylic acid **10a** (1.0 equiv.; 0.2 mmol; 45.9 mg), dineopentyl(vinyl)sulfonium triflate **9c** (1.5 equiv.; 0.3 mmol; 105 mg), 4CzIPN (0.05 equiv.; 0.01 mmol; 7.9 mg) and potassium phosphate (1.0 equiv.; 0.2 mmol; 42.4 mg) were introduced into a Schlenk tube. The atmosphere was exchanged to argon and degassed dry MeCN (1.3 mL, 0.15 M, previously degassed through 10 min argon sparging) was introduced through a syringe. The vessel was sealed and placed in a glass-wall water bath with temperature controlled between 20 - 25 °C. The reaction was irradiated through the glass wall with blue light (Kessil lamp A160WE Tuna Blue Saltwater LED Light 40W) for 24 h under moderate stirring (500 rpm). The vessel was removed from the water bath and the reaction mixture transferred into a 5 mL Young tube or a vial with Teflon-coated cap (washing the original vessel with DCM to ensure quantitative transfer). The solvent was removed blowing nitrogen. The corresponding amine (2.5 equiv.; 0.5 mmol), potassium phosphate (1.5 equiv.; 0.3 mmol; 63.6 mg) and dry acetonitrile (400 µL; 0.5 M) were added. The mixture was heated to 80 °C without irradiation for 24 h under vigorous stirring (700 rpm), after which it was filtered through celite and eluted with DCM. Volatiles were evaporated under reduced pressure and the residue was subjected to chromatography purification on silica gel to afford final compounds.

4.4. General procedure D

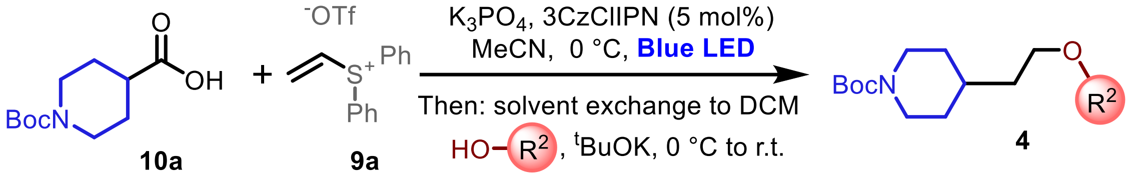

Diphenyl(vinyl)sulfonium triflate **9a** (1.5 equiv.; 0.3 mmol; 109 mg) was introduced into a Schlenk tube and the atmosphere was exchanged to argon. Then, degassed dry MeCN (1.3 mL, 0.15 M, previously degassed through 10 min argon sparging) was added through a syringe and the resultant solution was cooled down to 0 °C. 1-(*tert*-butoxycarbonyl)piperidine-4-carboxylic acid **10a** (1.0 equiv.; 0.2 mmol; 45.9 mg), 3CzClIPN (0.05 equiv.; 0.01 mmol; 6.6 mg) and potassium phosphate (1.0 equiv.; 0.2 mmol; 42.4 mg) were added to the solution at 0 °C. The vessel was sealed and placed in a glass-wall 0 °C bath. The reaction was irradiated through the glass wall with blue light (Kessil lamp A160WE Tuna Blue Saltwater LED Light 40W) for 8 h under moderate stirring (500 rpm). To avoid moisture condensation in the glass wall, which may impact the quality of irradiation, a generous flux of dry nitrogen was directed towards the cold glass wall, exactly where the light source points (see Fig. S3, the colorless plastic tube supplies dry nitrogen). The vessel was then taken out from the low temperature bath and the solvent was removed under vacuum using a standard Schlenk manifold. Dry DCM (1.3 mL, 0.15 M) was introduced into the vessel and cooled down to 0 °C. Aliphatic alcohol (2.5 equiv.; 0.5 mmol) and potassium *tert*-butoxide (1.1 equiv.; 0.22 mmol; 24.7 mg) were added to the reaction mixture at 0 °C. The mixture was slowly warmed up to room temperature, leaving the cold bath melting, and left stirring (700 ppm) at room temperature until complete consumption of sulfonium intermediate monitored by analysing a small aliquot via ^{1}H-NMR *(disappearance of signal at 4.30 ppm).* The mixture was filtered through a thin layer of celite and eluted with DCM. Volatiles were evaporated under reduced pressure and the residue was subjected to chromatography purification on silica gel to afford final compounds.

4.5. Reaction set-up for irradiation of mixtures with blue LEDs

This set up is a modified version of the water-cooled set up originally reported by MacMillan *et al.*[11] Throughout the irradiation, the temperature of the water was monitored with a thermometer to ensure temperature control.

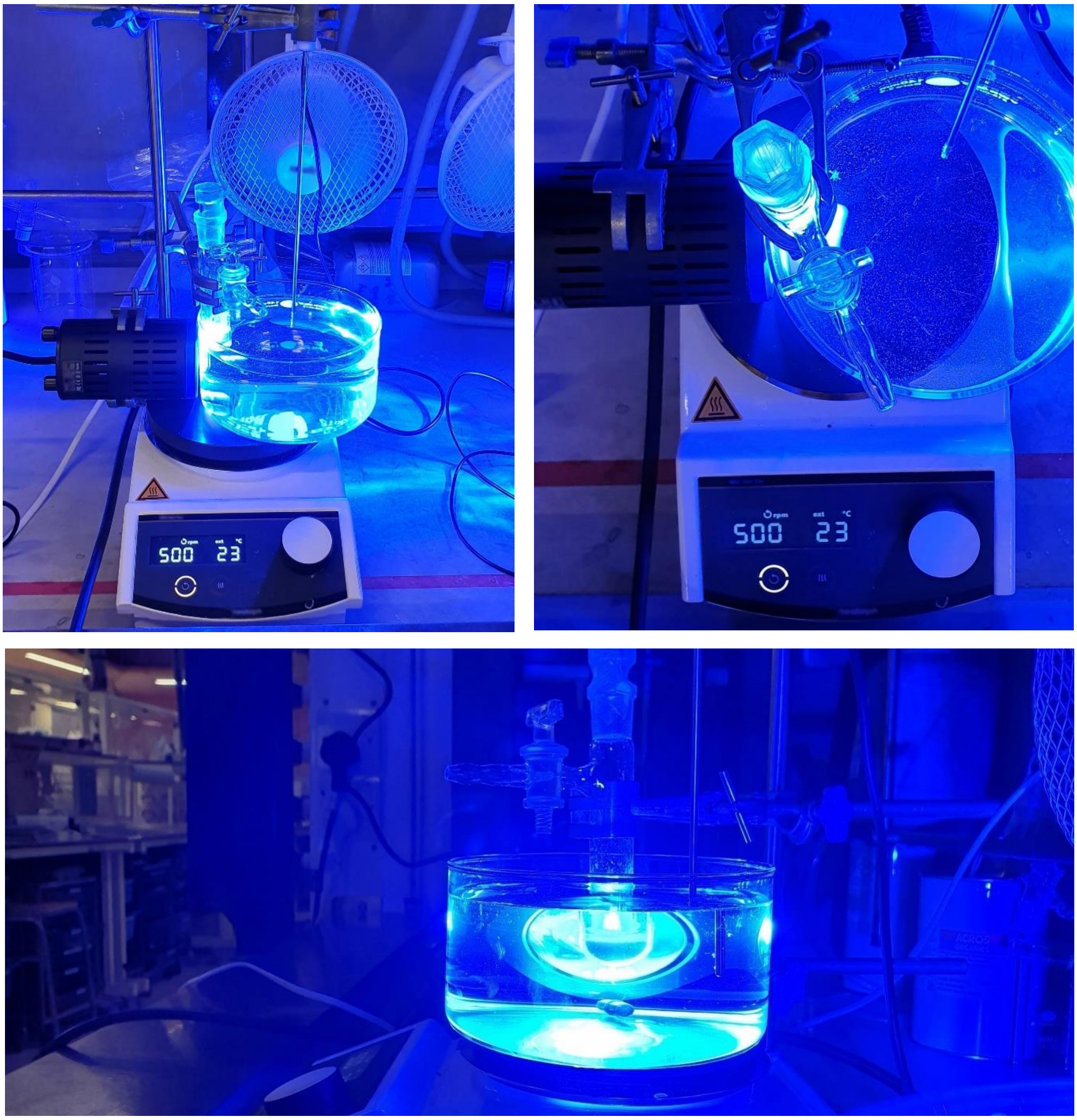

Fig. S2: Visual details of the photochemical reaction set-up: front, top, and lateral views.

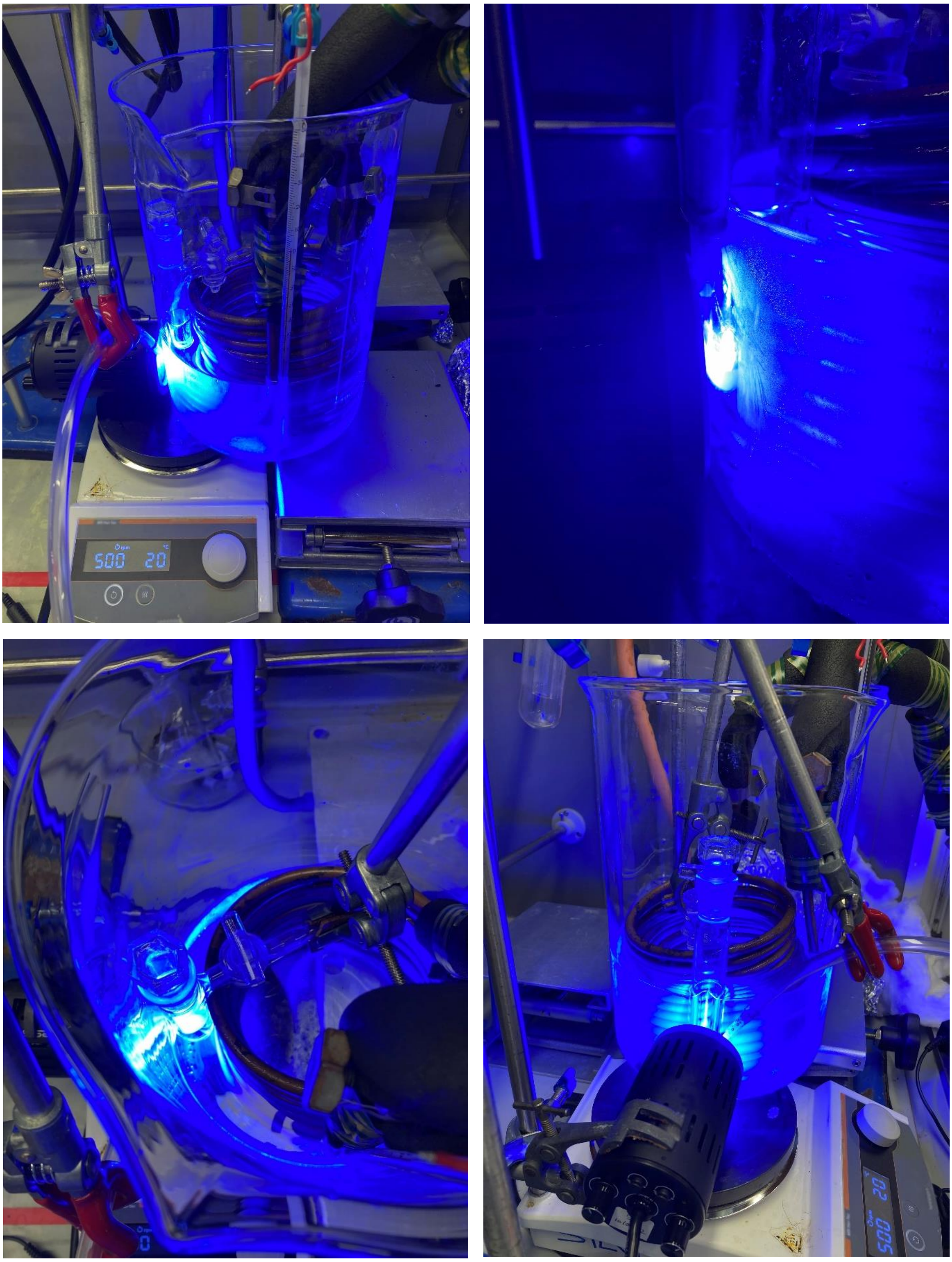

Fig. S3: Visual details of the photochemical reaction set-up at 0 °C: front, top, and lateral views.

4.6. Reaction products and characterization

tert-butyl 4-(2-mercaptoethyl)piperidine-1-carboxylate (**4d**)

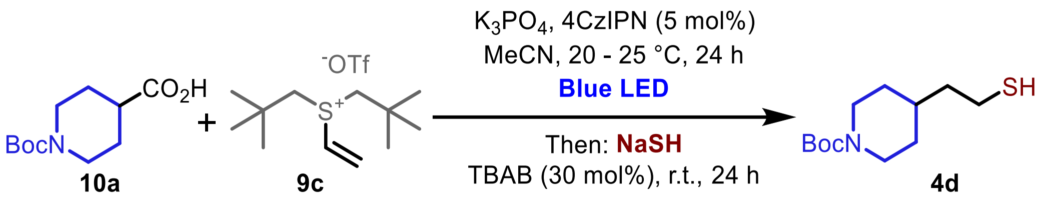

Prepared with a modification of general procedure A, using **10a** (1.0 equiv.; 0.2 mmol; 45.9 mg), dineopentyl(vinyl)sulfonium triflate **9c** (1.5 equiv.; 0.3 mmol; 105 mg), 4CzIPN (0.05 equiv.; 0.01 mmol; 7.9 mg), potassium phosphate (1.0 equiv.; 0.2 mmol; 42.4 mg). The vessel was then removed from the water bath, $NaSH{\cdot}xH_2O$ (118 mg) and catalytic tetrabutylammonium bromide (0.3 equiv.; 0.06 mmol; 19.3 mg) were added and the reaction was left stirring for 24 h at room temperature. After that, 4 mL of a degassed saturated solution of NH_4Cl were added and the reaction left stirring for 10 min under argon. The mixture was then transferred to a separatory funnel and extracted with DCM (10 mL x 3). The organic phases were collected and dried over anhydrous $MgSO_4$, filtered and the solvent removed under reduced pressure. The crude residue was purified by flash column chromatography (SiO_2; *to prevent the dimerization of the product, all the solvents were degassed with the sonicator, left bubbling with argon for 10 min and finally the column was run under argon*; gradient 95:5 to 85:15 hexane:ethyl acetate) to afford compound **4d** (30 mg; 61%) as a yellow oil. **R$_f$** (90:10 hexane:ethyl acetate) 0.2 ; **IR** (film) ν_{max}/cm^{-1}: 2972, 2925, 2555, 1689, 1420, 1366, 127, 1243, 1162, 1121, 1083, 1004, 933, 865, 768; **^{1}H NMR** ($CDCl_3$, 500 MHz) δ (ppm): 4.07 (d, J = 13.2 Hz, 2H), 2.67 (td, J = 13.2, 2.6 Hz, 2H), 2.55 (q, J = 7.3 Hz, 2H), 1.67 – 1.60 (m, 2H), 1.59 – 1.52 (m, 3H), 1.44 (s, 9H), 1.32 (t, J = 7.6 Hz, 1H), 1.15 – 1.01 (m, 2H); **^{13}C NMR** ($CDCl_3$, 126 MHz) δ (ppm): 155.0, 79.4, 44.0, 40.8, 34.8, 31.8, 28.6, 22.0; **HRMS** (ESI-TOF) mass calculated for $[M+Na]^+$ ($C_{12}H_{23}NO_2SNa^+$) expected *m/z* 268.1342; found *m/z* 268.1341.

tert-butyl 4-(2-((2-hydroxyethyl)thio)ethyl)piperidine-1-carboxylate (**4a**)

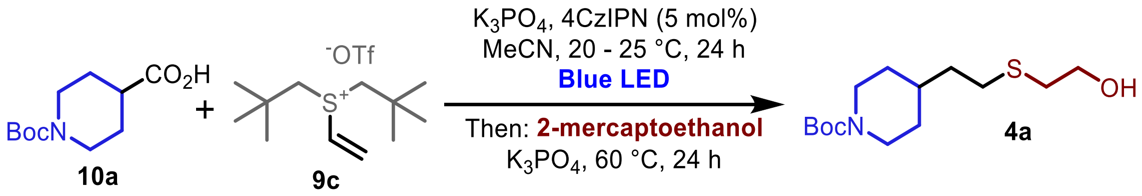

Prepared following general procedure A, using **10a** (1.0 equiv.; 0.2 mmol; 45.9 mg), dineopentyl(vinyl)sulfonium triflate **9c** (1.5 equiv.; 0.3 mmol; 105 mg), 4CzIPN (0.05 equiv.;

0.01 mmol; 7.9 mg), potassium phosphate (1.0 equiv.; 0.2 mmol; 42.4 mg) + (1.5 equiv.; 0.3 mmol; 63.6 mg) and 2-mercaptoethanol (2.5 equiv.; 0.5 mmol; 35.1 µL). The crude residue was purified by flash column chromatography (SiO_2; gradient 9:1 to 6:4 DCM:ethyl acetate) to afford compound **4a** (41.2 mg; 71%) as a pale yellow oil. **R$_f$** (7:3 DCM:ethyl acetate) 0.4; **IR** (film) ν_{max}/cm^{-1}: 3416, 2923, 1690, 1425, 1366, 1245, 1150; **^{1}H NMR** ($CDCl_3$, 500 MHz) δ (ppm): 4.07 (br s, 2H), 3.75 – 3.68 (m, 2H), 2.77 – 2.61 (m, 4H), 2.59 – 2.51 (m, 2H), 2.19 (br s, 1H), 1.65 (d, J = 12.7 Hz, 2H), 1.59 – 1.49 (m, 3H), 1.44 (s, 9H), 1.16 – 1.02 (m, 2H); **^{13}C NMR** ($CDCl_3$, 126 MHz) δ (ppm): 155.0, 79.4, 60.4, 44.0, 36.4, 35.4, 35.2, 32.0, 29.0, 28.6; **HRMS** (ESI-TOF) mass calculated for $[M+Na]^+$ ($C_{14}H_{27}NO_3SNa^+$) expected *m/z* 312.1604; found *m/z* 312.1603.

tert-butyl 4-(2-(cyclohexylthio)ethyl)piperidine-1-carboxylate (**4e**)

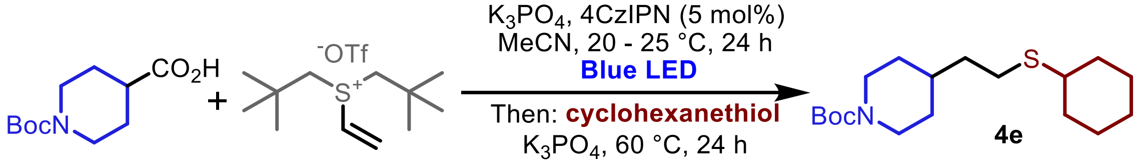

10a **9c** **4e**

Prepared following general procedure A, using **10a** (1.0 equiv.; 0.2 mmol; 45.9 mg), dineopentyl(vinyl)sulfonium triflate **9c** (1.5 equiv.; 0.3 mmol; 105 mg), 4CzIPN (0.05 equiv.; 0.01 mmol; 7.9 mg), potassium phosphate (1.0 equiv.; 0.2 mmol; 42.4 mg) + (1.5 equiv.; 0.3 mmol; 63.6 mg) and cyclohexanethiol (2.5 equiv.; 0.5 mmol; 61.2 µL). The crude residue was purified by flash column chromatography (SiO_2; gradient 99.5:0.5 to 95:5 DCM:ethyl acetate) to afford compound **4e** (51.3 mg; 78%) as a transparent oil. **R$_f$** (199:1 DCM:ethyl acetate) 0.25; **IR** (film) ν_{max}/cm^{-1}: 2926, 2851, 1692, 1421,1365, 1242, 1158, 768; **^{1}H NMR** ($CDCl_3$, 500 MHz) δ (ppm): 4.06 (d, J = 13.1 Hz, 2H), 2.71 – 2.57 (m, 3H), 2.54 (t, J = 7.0 Hz, 2H), 1.98 – 1.89 (m, 2H), 1.79 – 1.71 (m, 2H), 1.68 – 1.58 (m, 3H), 1.54 – 1.47 (m, 3H), 1.43 (s, 9H), 1.35 – 1.20 (m, 5H), 1.13 – 1.02 (m, 2H); **^{13}C NMR** ($CDCl_3$, 126 MHz) δ (ppm): 155.0, 79.3, 44.0, 43.7, 36.7, 35.3, 33.8, 32.0, 28.6, 27.4, 26.3, 26.0; **HRMS** (ESI-TOF) mass calculated for $[M+Na]^+$ ($C_{18}H_{33}NO_2SNa^+$) expected *m/z* 350.2124; found *m/z* 350.2118.

tert-butyl 4-(2-(phenylthio)ethyl)piperidine-1-carboxylate (**4f**)

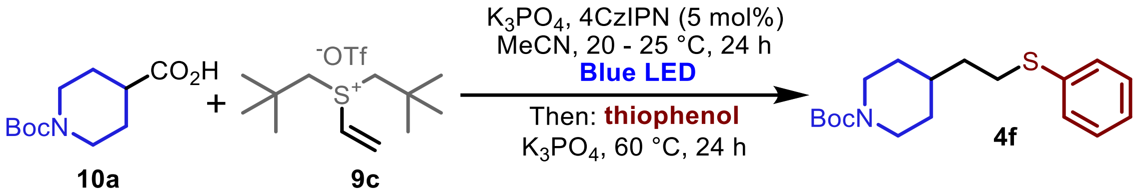

Prepared following general procedure A, using **10a** (1.0 equiv.; 0.2 mmol; 45.9 mg), dineopentyl(vinyl)sulfonium triflate **9c** (1.5 equiv.; 0.3 mmol; 105 mg), 4CzIPN (0.05 equiv.; 0.01 mmol; 7.9 mg), potassium phosphate (1.0 equiv.; 0.2 mmol; 42.4 mg) + (1.5 equiv.; 0.3 mmol; 63.6 mg) and thiophenol (2.5 equiv.; 0.5 mmol; 51.3 µL). The crude residue was purified by flash column chromatography (SiO_2; gradient 95:5 to 9:1 hexane:ethyl acetate) to afford compound **4f** (53.9 mg; 84%) as a pale yellow solid. **R$_f$** (9:1 hexane:ethyl acetate) 0.4; **M.P.** = 50 – 51 °C; **IR** (film) ν_{max}/cm^{-1}: 2927, 2852, 1691, 1479, 1422, 1243, 1159, 738; **^{1}H NMR** ($CDCl_3$, 500 MHz) δ (ppm): 7.34 – 7.25 (m, 4H), 7.19 – 7.14 (m, 1H), 4.07 (br s, 2H), 2.94 (t, *J* = 7.5 Hz, 2H), 2.75 – 2.58 (m, 2H), 1.66 (d, *J* = 13.0 Hz, 2H), 1.63 – 1.52 (m, 3H), 1.45 (s, 9H), 1.17 – 1.02 (m, 2H); **^{13}C NMR** ($CDCl_3$, 126 MHz) δ (ppm): 154.9, 136.7, 129.1, 129.0, 126.0, 79.4, 44.0, 35.8, 35.2, 31.9, 31.0, 28.6; **HRMS** (ESI-TOF) mass calculated for $[M+Na]^+$ ($C_{18}H_{27}NO_2SNa^+$) expected *m/z* 344.1654; found *m/z* 344.1653.

((*S*)-3-((2-(1-(*tert*-butoxycarbonyl)piperidin-4-yl)ethyl)thio)-2-methylpropanoyl)-*L*-proline (**4g**)

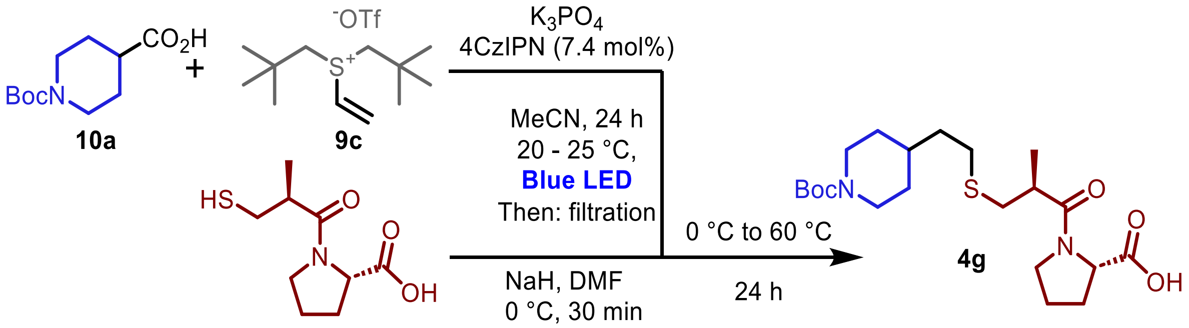

Prepared with a modification of general procedure A, using **10a** (1.4 equiv.; 0.28 mmol; 64.2 mg), dineopentyl(vinyl)sulfonium triflate **9c** (1.54 equiv.; 0.31 mmol; 108 mg), 4CzIPN (0.074 equiv.; 0.015 mmol; 11.7 mg) and potassium phosphate (1.4 equiv.; 0.28 mmol; 59.4 mg). After 24 h under irradiation, the mixture was filtered through celite and eluted with DCM. The solution was transferred into another Schlenk tube and the solvent removed by blowing nitrogen. Dry DMF (250 µL) was added, and the mixture cooled down to 0 °C (Solution A).

Captopril (1.0 equiv.; 0.2 mmol; 43.4 mg) and dry DMF (500 µL) were added in a separate vessel and cooled down to 0 °C. NaH (60% in oil; 2.0 equiv.; 0.4 mmol; 16 mg) was then slowly added and the reaction left stirring at 0 °C for 30 min (Solution B).

Solution B was transferred to solution A at 0 °C washing the vessel using other 550 µL of DMF. The reaction was left for 24 h at 60 °C under moderate stirring (700 rpm). Saturated NH_4Cl solution (10 mL) was added to the reaction mixture and extracted with DCM (25 mL x 3). The organic phases were collected and washed once with brine (20 mL) and dried over anhydrous $MgSO_4$. The solvent was then removed by rotary evaporation. The crude residue was purified by flash column chromatography (SiO_2; gradient 99:1 to 96:4 DCM:methanol + 0.2% AcOH) to afford compound **4g** (45.8 mg; 53%) as a yellow oil. **R_f** (9:1 DCM:methanol + 0.2% AcOH) 0.4; **$[\alpha]_D^{25}$** = -88 (c = 1.00 $CHCl_3$); **IR** (film) ν_{max}/cm^{-1}: 2974, 2926, 2845, 1741, 1687, 1644, 1424, 1366, 1318, 1275, 1243, 1157, 1117, 1090, 964, 918, 864, 769, 732; **^{1}H NMR** ($CDCl_3$, 500 MHz) δ (ppm): 4.64 (dd, *J* = 8.1, 2.5 Hz, 1H), 4.07 (br s, 2H), 3.67 – 3.54 (m, 2H), 2.91 – 2.78 (m, 2H), 2.75 – 2.62 (m, 2H), 2.61 – 2.46 (m, 4H), 2.13 – 1.94 (m, 3H), 1.64 (d, *J* = 13.1 Hz, 2H), 1.55 – 1.47 (m, 3H), 1.45 (s, 9H), 1.24 (d, *J* = 6.5 Hz, 3H), 1.14 – 1.02 (m, 2H); **^{13}C NMR** ($CDCl_3$, 126 MHz) δ (ppm): 177.7, 171.9, 155.0, 79.5, 60.2, 48.1, 44.1, 39.3, 36.4, 35.8, 35.2, 32.0, 30.6, 28.6, 27.3, 25.0, 17.8;

HRMS (ESI-TOF) mass calculated for $[M-H]^-$ ($C_{21}H_{35}N_2O_5S^-$) expected *m/z* 427.2272; found *m/z* 427.2272.

tert-butyl 4-(2-hydroxyethyl)piperidine-1-carboxylate (**4h**)

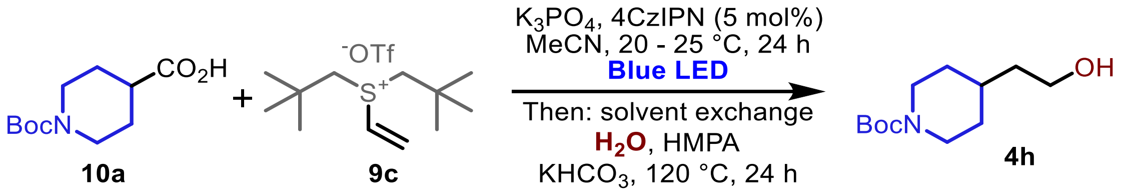

Irradiated following a modification of general procedure A, using **10a** (1.0 equiv.; 0.2 mmol; 45.9 mg), dineopentyl(vinyl)sulfonium triflate **9c** (1.5 equiv.; 0.3 mmol; 105 mg), 4CzIPN (0.05 equiv.; 0.01 mmol; 7.9 mg), potassium phosphate (1.0 equiv.; 0.2 mmol; 42.4 mg). After photochemical step, the crude was filtered through celite pad and eluted with DCM, concentrated under reduced pressure and transferred in a 5 mL Young tube. Solvent was removed by blowing nitrogen and H_2O (45 equiv.; 9.0 mmol; 162 µL), potassium bicarbonate (1.3 equiv.; 0.26 mmol; 100 mg) and HMPA (800 µL; 0.25 M) were added. The reaction was heated to 120 °C for 24 h. After completion, the mixture was cooled down to room temperature, diluted with water (6 mL), added 5% LiCl solution (4 mL), extracted with ethyl acetate (6 mL

x 3). The combined organic layers were washed with brine (5 mL), dried over anhydrous $MgSO_4$ and the solvent was removed by rotary evaporation. The crude residue was purified by flash column chromatography (SiO_2; gradient 6:4 to 3:7 DCM:ethyl acetate) to afford compound **4h** (24.1 mg; 52%) as a pale yellow oil. **R$_f$** (6:4 DCM:ethyl acetate) 0.3; **IR** (film) ν_{max}/cm^{-1}: 3429, 2926, 2857, 1691, 1426, 1367, 1247, 1168, 768; **^{1}H NMR** ($CDCl_3$, 500 MHz) δ (ppm): 4.06 (br s, 2H), 3.70 (t, J = 6.6 Hz, 2H), 2.76 – 2.60 (m, 2H), 1.71 – 1.64 (m, 2H), 1.62 – 1.55 (m, 1H), 1.53 – 1.49 (m, 2H), 1.44 (s, 9H), 1.17 – 1.07 (m, 2H); **^{13}C NMR** ($CDCl_3$, 126 MHz) δ (ppm): 155.0, 79.4, 60.4, 44.3, 39.4, 32.7, 32.3, 28.6; **HRMS** (ESI-TOF) mass calculated for $[M+Na]^+$ ($C_{12}H_{23}NO_3Na^+$) expected *m/z* 252.1570; found *m/z* 252.1571.

tert-butyl 4-(2-(benzyloxy)ethyl)piperidine-1-carboxylate (**4i**)

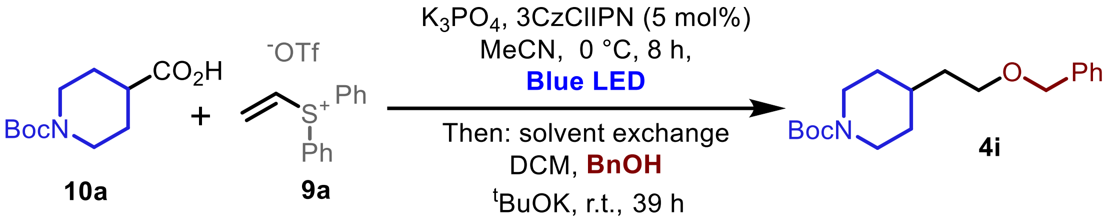

Prepared following general procedure D, using **10a** (1.0 equiv.; 0.2 mmol; 45.9 mg), diphenyl(vinyl)sulfonium triflate **9a** (1.5 equiv.; 0.3 mmol; 108.7 mg), 3CzClIPN (0.05 equiv.; 0.01 mmol; 6.6 mg), potassium phosphate (1.0 equiv.; 0.2 mmol; 42.4 mg). Then, benzyl alcohol (2.5 equiv.; 0.5 mmol; 51.7 µL) and potassium *tert*-butoxide (1.1 equiv.; 0.22 mmol; 24.7 mg) were used. Reaction was continued at r.t. for 39 h. The crude residue was purified by flash column chromatography (SiO_2; gradient 9:1 to 85:15 toluene:Et_2O). The compound was purified a second time by flash column chromatography (SiO_2; gradient 9:1 to 85:15 hexane: ethyl acetate) to afford compound **4i** (25.6 mg; 40%) as a colourless oil. **R$_f$** (9:1 toluene:Et_2O) 0.35; **IR** (film) ν_{max}/cm^{-1}: 2974, 2925, 2854, 1692, 1421, 1365, 1277, 1171, 736; **^{1}H NMR** ($CDCl_3$, 500 MHz) δ (ppm): 7.37 – 7.30 (m, 4H), 7.30 – 7.26 (m, 1H), 4.50 (s, 2H), 4.06 (br s, 2H), 3.51 (t, J = 6.3 Hz, 2H), 2.75 – 2.60 (m, 2H), 1.68 – 1.58 (m, 3H), 1.58 – 1.53 (m, 2H), 1.45 (s, 9H), 1.15 – 1.03 (m, 2H); **^{13}C NMR** ($CDCl_3$, 126 MHz) δ (ppm): 155.0, 138.6, 128.5, 127.8, 127.7, 79.3, 73.1, 67.8, 44.1, 36.4, 33.1, 32.3, 28.6; **HRMS** (ESI-TOF) mass calculated for $[M+Na]^+$ ($C_{19}H_{29}NO_3Na^+$) expected *m/z* 342.2040; found *m/z* 342.2021.

tert-butyl 4-(2-cyclobutoxyethyl)piperidine-1-carboxylate (**4j**)

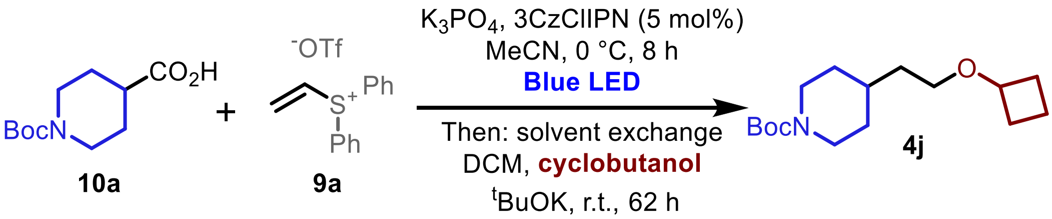

Prepared following general procedure D, using **10a** (1.0 equiv.; 0.2 mmol; 45.9 mg), diphenyl(vinyl)sulfonium triflate **9a** (1.5 equiv.; 0.3 mmol; 108.7 mg), 3CzClIPN (0.05 equiv.; 0.01 mmol; 6.6 mg), potassium phosphate (1.0 equiv.; 0.2 mmol; 42.4 mg). Then, cyclobutanol (2.5 equiv.; 0.5 mmol; 39.2 µL) and potassium *tert*-butoxide (1.1 equiv.; 0.22 mmol; 24.7 mg) were used. Reaction was continued at r.t. for 62 h. The crude residue was purified by flash column chromatography (SiO_2; gradient 95:5 to 8:2 toluene:Et_2O) to afford compound **4j** (21.3 mg; 38%) as a colourless oil. **R_f** (95:5 toluene:Et_2O) 0.25; **IR** (film) ν_{max}/cm^{-1}: 2974, 2929, 2857, 1695, 1421, 1365, 1243, 1149, 1117, 769; **^{1}H NMR** ($CDCl_3$, 500 MHz) δ (ppm): 4.11 – 4.01 (m, 2H), 3.93 – 3.84 (m, 1H), 3.35 (t, J = 6.5 Hz, 2H), 2.68 (td, J = 13.1, 2.7 Hz, 2H), 2.25 – 2.13 (m, 2H), 1.95 -1.83 (m, 2H), 1.71 – 1.61 (m, 3H), 1.59 – 1.53 (m, 1H), 1.53 – 1.46 (m, 3H), 1.45 (s, 9H), 1.16 – 1.03 (m, 2H); **^{13}C NMR** ($CDCl_3$, 126 MHz) δ (ppm): 155.0, 79.3, 73.3, 65.0, 44.3, 36.5, 33.0, 32.3, 30.6, 28.6, 12.7; **HRMS** (ESI-TOF) mass calculated for $[M+Na]^+$ ($C_{16}H_{29}NO_3Na^+$) expected *m/z* 306.2040; found *m/z* 306.2043.

tert-butyl 4-(2-phenoxyethyl)piperidine-1-carboxylate (**4b**)

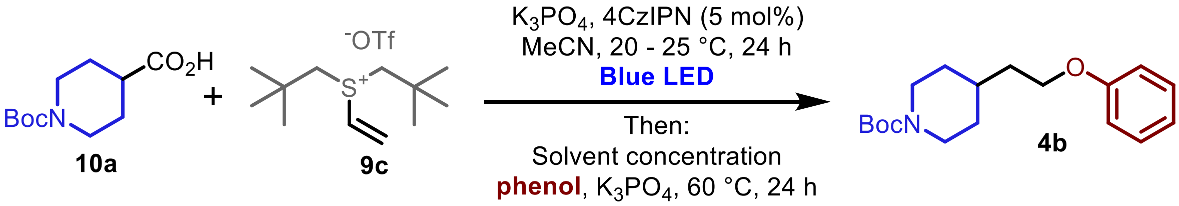

Prepared following general procedure B, using **10a** (1.0 equiv.; 0.2 mmol; 45.9 mg), dineopentyl(vinyl)sulfonium triflate **9c** (1.5 equiv.; 0.3 mmol; 105 mg), 4CzIPN (0.05 equiv.; 0.01 mmol; 7.9 mg), potassium phosphate (1.0 equiv.; 0.2 mmol; 42.4 mg). After the photochemical step the mixture was transferred in a 5 mL Young tube with phenol (2.5 equiv.; 0.5 mmol; 47.1 mg) and potassium phosphate (1.5 equiv.; 0.3 mmol; 63.6 mg). The crude residue was purified by flash column chromatography (SiO_2; gradient 95:5 to 85:15 hexane:ethyl acetate) to afford compound **4b** (38 mg; 62%) as a white solid. **R_f** (8:2 hexane:ethyl acetate) 0.5; **M.P.** = 68 – 69 °C; **IR** (film) ν_{max}/cm^{-1}: 2974, 2928, 2864, 1691, 1599, 1587, 1497, 1473, 1447, 1422, 1392, 1365, 1277, 1244, 1171, 1142, 1079, 1032, 979,

866, 754, 692; **^{1}H NMR** ($CDCl_3$, 500 MHz) δ (ppm): 7.31 – 7.27 (m, 2H), 6.94 (t, *J* = 7.4 Hz, 1H), 6.89 (d, *J* = 8.4Hz, 2H), 4.09 (br s, 2H), 4.01 (t, *J* = 6.0 Hz, 2H) 2.68 – 2.60 (m, 2H), 1.78 – 1.65 (m, 5H), 1.46 (s, 9H), 1.24 – 1.10 (m, 2H); **^{13}C NMR** ($CDCl_3$, 126 MHz) δ (ppm): 159.1, 155.0, 129.6, 120.8, 114.6, 79.4, 65.3, 44.0, 35.9, 33.1, 32.2, 28.6; **HRMS** (ESI-TOF) mass calculated for $[M+Na]^+$ ($C_{18}H_{27}NO_3Na^+$) expected *m/z* 328.1883; found *m/z* 328.1876.

tert-butyl 4-(2-(((4*R*,4a*R*,7*S*,7a*R*,12b*S*)-7-hydroxy-3-methyl-2,3,4,4a,7,7a-hexahydro-1*H*-4,12-methanobenzofuro[3,2-*e*]isoquinolin-9-yl)oxy)ethyl)piperidine-1-carboxylate (**4k**)

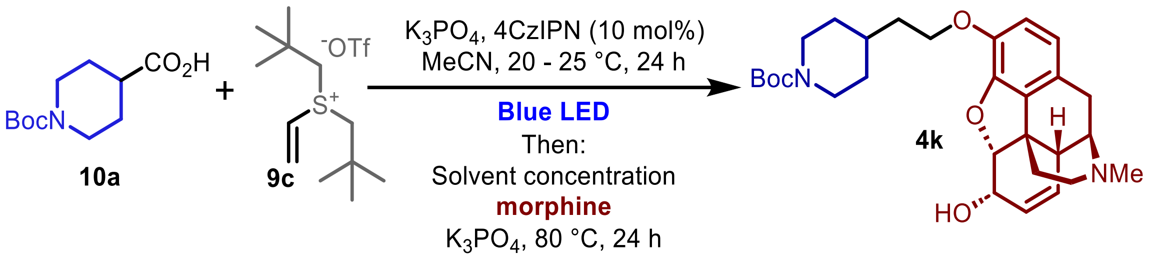

Prepared following a modification of general procedure B, using **10a** (2.0 equiv.; 0.2 mmol; 45.9 mg), dineopentyl(vinyl)sulfonium triflate **9c** (2.2 equiv.; 0.22 mmol; 77.1 mg), 4CzIPN (0.10 equiv.; 0.01 mmol; 7.9 mg), potassium phosphate (2.0 equiv.; 0.2 mmol; 42.4 mg). After photochemical step, the crude was transferred in a 5 mL Young tube and solvent was removed by blowing nitrogen and under high vacuum using a standard Schlenk manifold. Then morphine (1.0 equiv.; 0.1 mmol; 28.5 mg), potassium phosphate (1.0 equiv.; 0.1 mmol; 21.2 mg) and dry acetonitrile (660 µL; 0.15 M) were added. The reaction was heated to 80 °C for 24 h. Then the mixture was cooled down to room temperature and saturated $NaHCO_3$ solution was added (5 mL) and the mixture extracted with DCM (5 mL x 4). The organic layers were collected and washed with brine (5 mL), dried over anhydrous $MgSO_4$ and concentrated under reduced pressure. The crude residue was purified by flash column chromatography (SiO_2; gradient 95:5 to 94:6 $CHCl_3$:MeOH + 2% ammonia in water (35%)). The compound was purified a second time by flash column chromatography (SiO_2; gradient 95:5 to 93:7 DCM:MeOH + 1% ammonia in water (35%)) to afford compound **4k** (38.8 mg; 78%) as white solid. **R_f** (95:5 $CHCl_3$:MeOH + 2% ammonia in water (35%)) 0.3; **$[\alpha]_D^{25}$** = -52 (c = 1.00 $CHCl_3$); **M.P.** = 62 – 63 °C; **IR** (film) ν_{max}/cm^{-1}: 3412, 2926, 2856, 1690, 1603, 1499, 1446, 1367, 1277, 1248, 1170, 759; **^{1}H NMR** ($CDCl_3$, 500 MHz) δ (ppm): 6.64 (d, *J* = 8.1 Hz, 1H), 6.54 (d, *J* = 8.1 Hz, 1H), 5.73 – 5.66 (m, 1H), 5.32 – 5.26 (m, 1H), 4.88 (dd, *J* = 6.6, 1.2 Hz, 1H), 4.21 – 4.15 (m, 1H), 4.14 – 3.98 (m, 4H), 3.34 (dd, *J* = 6.2, 3.3 Hz, 1H), 3.03 (d, *J* = 18.6 Hz, 1H), 2.88 (br s, 1H), 2.75 – 2.64 (m, 3H), 2.59 (dd, *J* = 12.2, 4.5 Hz, 1H), 2.44 (s, 3H), 2.43 – 2.36 (m, 1H), 2.29 (dd, *J* = 18.6, 6.3 Hz, 1H), 2.06 (td, *J* = 12.5, 5.1 Hz, 1H), 1.90 –

1.84 (m, 1H), 1.76 – 1.63 (m, 5H), 1.45 (s, 9H), 1.20 – 1.09 (m, 2H); **^{13}C NMR** ($CDCl_3$, 126 MHz) δ (ppm): 155.0, 146.7, 141.6, 133.5, 131.3, 128.4, 127.5, 119.8, 114.9, 91.3, 79.4, 67.2, 66.4, 59.0, 46.6, 43.8, 43.2, 43.0, 40.9, 36.1, 35.9, 32.9, 32.2, 28.6, 20.6; **HRMS** (ESI-TOF) mass calculated for $[M+H]^+$ ($C_{29}H_{41}N_2O_5^+$) expected *m/z* 497.3010; found *m/z* 497.2997.

tert-butyl 4-(2-((5-((3aS,4S,6aR)-2-oxohexahydro-1*H*-thieno[3,4-*d*]imidazol-4-yl)pentanoyl)oxy)ethyl)piperidine-1-carboxylate (**4l**)

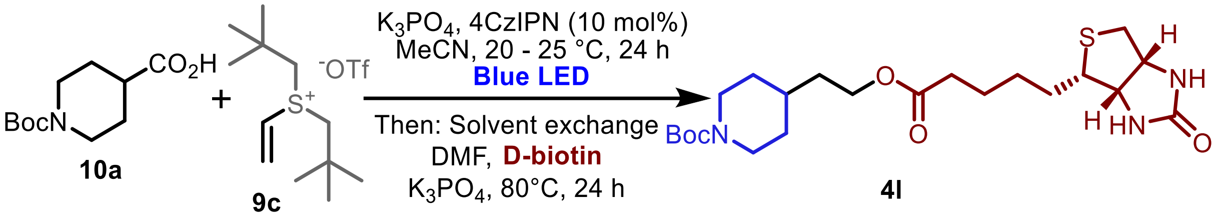

Prepared following a modification of general procedure A, using **10a** (2.0 equiv.; 0.2 mmol; 45.9 mg), dineopentyl(vinyl)sulfonium triflate **9c** (2.2 equiv.; 0.22 mmol; 77.1 mg), 4CzIPN (0.10 equiv.; 0.01 mmol; 7.9 mg), potassium phosphate (2.0 equiv.; 0.2 mmol; 42.4 mg). After photochemical step, the solvent was exchanged with DMF (0.66 mL; 0.15M) and D-biotin (1.0 equiv.; 0.1 mmol; 24.4 mg) and potassium phosphate (1.0 equiv.; 0.1 mmol; 21.2 mg) were added. The reaction was heated to 80 °C for 24 h. The crude residue was diluted in EtOAc (50 mL) and extracted with water (40 mL x 5) and brine (30 mL x 1). The solvent was evaporated under reduced pressure and the crude purified by flash column chromatography (SiO_2; gradient 99:3 to 95:5 DCM:MeOH) to afford compound **4l** (37.5 mg; 82%) as a white sticky solid. **R_f** (95:5 DCM:MeOH) 0.35; **$[\alpha]_D^{25}$** = +28 (c = 1.00 $CHCl_3$); **M.P.** = 75 – 76 °C; **IR** (film) ν_{max}/cm^{-1}: 3211, 2925, 2854, 1732, 1692, 1469, 1422, 1365, 1277, 1246, 1169, 1144, 1080, 1014, 971, 866, 761, 665, 604; **^{1}H NMR** ($CDCl_3$, 500 MHz) δ (ppm): 5.46 (br s, 1H), 5.10 (br s, 1H), 4.52 (dd, *J* = 7.7, 5.0, 1H), 4.32 (dd, *J* = 7.8, 4.6 Hz, 1H), 4.14 – 4.01 (m, 4H), 3.19 – 3.13 (m, 1H), 2.92 (dd, *J* = 12.9, 5.0 Hz, 1H), 2.74 (dd, *J* = 12.8, 1.1 Hz, 1H), 2.68 (t, *J* = 12.7 Hz, 2H), 2.33 (t, *J* = 7.4 Hz, 2H), 1.77 – 1.62 (m, 6H), 1.60 – 1.55 (m, 2H), 1.55 – 1.50 (m, 1H), 1.50 – 1.38 (m, 11H), 1.12 (qd, *J* = 12.4, 4.3 Hz, 2H); **^{1}H NMR** (DMSO-d_6, 500 MHz) δ (ppm): 6.42 (s, 1H), 6.35 (s, 1H), 4.30 (dd, *J* = 7.7, 5.0 Hz, 1H), 4.15 – 4.10 (m, 1H), 4.05 (t, *J* = 6.2 Hz, 2H), 3.90 (d, *J* = 13.0 Hz, 2H), 3.12 – 3.06 (m, 1H), 2.82 (dd, *J* = 12.4, 5.1 Hz, 1H), 2.67 (br s, 2H), 2.58 (d, *J* = 12.4 Hz, 1H), 2.29 (t, *J* = 7.4 Hz, 2H), 1.66 – 1.58 (m, 3H), 1.58 – 1.42 (m, 6H), 1.38 (s, 9H), 1.37 – 1.27 (m, 2H), 1.04 – 0.94 (m, 2H); **^{13}C NMR** ($CDCl_3$, 126 MHz) δ (ppm): 173.8, 163.4, 155.0, 79.4, 62.3, 62.1, 60.3, 55.5, 44.1, 40.7, 35.3, 34.0, 33.1,

32.1, 28.6, 28.5, 28.4, 24.9; **HRMS** (ESI-TOF) mass calculated for $[M+Na]^+$ ($C_{22}H_{37}N_3O_5SNa^+$) expected *m/z* 478.2346; found *m/z* 478.2338.

tert-butyl 4-(2-aminoethyl)piperidine-1-carboxylate (**4m**)

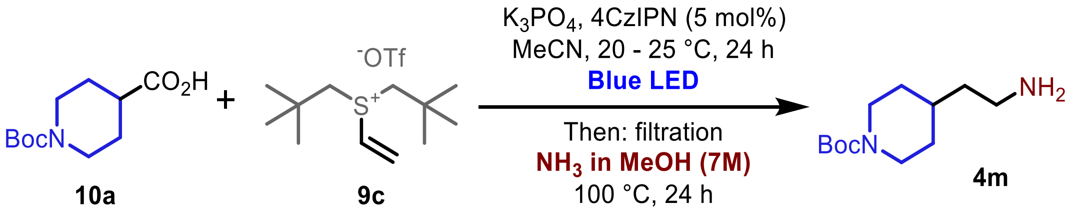

Prepared following a modification of general procedure C, using **10a** (1.0 equiv.; 0.2 mmol; 45.9 mg), dineopentyl(vinyl)sulfonium triflate **9c** (1.5 equiv.; 0.3 mmol; 105 mg), 4CzIPN (0.05 equiv.; 0.01 mmol; 7.9 mg), potassium phosphate (1.0 equiv.; 0.2 mmol; 42.4 mg). After the photochemical step, the crude was filtered through celite, collected in a microwave vial and the solvent removed blowing nitrogen. Ammonia (7M in methanol; 6 mL; 0.03M) was added and the reaction was heated to 100 °C for 24 h covered by a blast shield. A solution of NaOH (2 M; 5 mL) was added and the mixture extracted three times with DCM (20 mL x 3). The organic phases were collected and dried with anhydrous $MgSO_4$ and the solvent removed by rotary evaporator. The crude residue was purified by flash column chromatography (SiO_2; gradient 9:1 to 7:3 DCM:MeOH + 0.5% ammonia in water (35%)) to afford compound **4m** (36 mg; 79%) as a yellow oil. $\mathbf{R_f}$ (8:2 DCM:MeOH + 0.5% ammonia in water (35%)) 0.1; **IR** (film) ν_{max}/cm^{-1}: 3369, 2973, 2924, 2855, 1688, 1473, 1423, 1366, 1338, 1277, 1241, 1159, 1099, 1004, 970, 924, 864, 815, 767, 533; **^{1}H NMR** ($CDCl_3$, 500 MHz) δ (ppm): 5.53 (br s, 2H), 4.07 (br s, 2H), 2.92 (t, *J* = 7.5 Hz, 2H), 2.75 – 2.61 (m, 2H), 1.70 – 1.48 (m, 5H), 1.44 (s, 9H), 1.19 – 1.05 (m, 2H); **^{13}C NMR** ($CDCl_3$, 126 MHz) δ (ppm): 155.0, 79.5, 44.0, 38.5, 37.0, 33.6, 32.0, 28.6; **HRMS** (ESI-TOF) mass calculated for $[M+H]^+$ ($C_{12}H_{25}N_2O_2^+$) expected *m/z* 229.1911; found *m/z* 229.1911.

tert-butyl 4-(2-(benzylamino)ethyl)piperidine-1-carboxylate (**4c**)

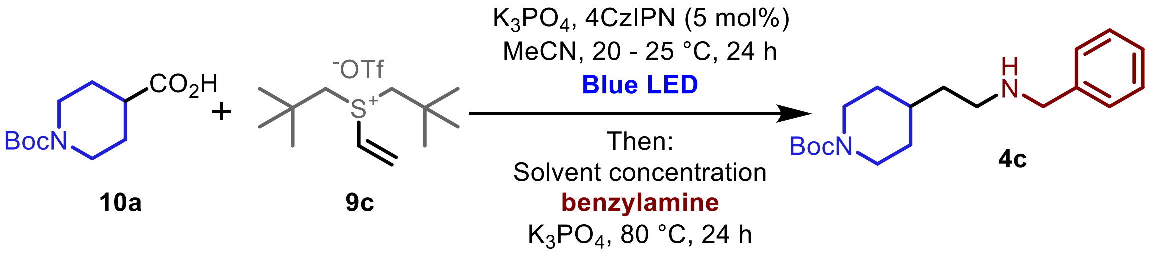

Prepared following general procedure C, using **10a** (1.0 equiv.; 0.2 mmol; 45.9 mg), dineopentyl(vinyl)sulfonium triflate **9c** (1.5 equiv.; 0.3 mmol; 105 mg), 4CzIPN (0.05 equiv.;

0.01 mmol; 7.9 mg), potassium phosphate (1.0 equiv.; 0.2 mmol; 42.4 mg). After photochemical step, the crude was transferred in a 2 mL teflon-coated screw cap vial with benzylamine (2.5 equiv.; 0.5 mmol; 54.6 µL) and potassium phosphate (1.5 equiv.; 0.3 mmol; 63.6 mg). The crude residue was purified by flash column chromatography (SiO_2; gradient 3:7 to 2:8 DCM:ethyl acetate + 0.5% ammonia in water (35%)) followed by 98:2 to 95:5 DCM:MeOH + 0.5% ammonia in water (35%)) to afford compound **4c** (41.1 mg; 65%) as a yellow oil. **R$_f$** (98:2 DCM:MeOH + 0.5% ammonia in water (35%)) 0.2; **IR** (film) ν_{max}/cm^{-1}: 3316, 2972, 2923, 2851, 1690, 1422, 1365, 1244, 1169, 737; **^{1}H NMR** ($CDCl_3$, 500 MHz) δ (ppm): 7.34 – 7.29 (m, 4H), 7.27 – 7.22 (m, 1H), 4.05 (br s, 2H), 3.79 (s, 2H), 2.72 – 2.59 (m, 4H), 1.84 (br s, 1H), 1.62 (d, *J* = 13.1 Hz, 2H), 1.49 – 1.45 (m, 3H), 1.44 (s, 9H), 1.15 – 1.03 (m, 2H); **^{13}C NMR** ($CDCl_3$, 126 MHz) δ (ppm): 155.0, 140.2, 128.6, 128.3, 127.2, 79.3, 54.1, 46.7, 43.8, 36.8, 34.1, 32.3, 28.6; **HRMS** (ESI-TOF) mass calculated for $[M+H]^+$ ($C_{19}H_{31}N_2O_2^+$) expected *m/z* 319.2380; found *m/z* 319.2382.

tert-butyl 4-(2-(pyrrolidin-1-yl)ethyl)piperidine-1-carboxylate (**4n**)

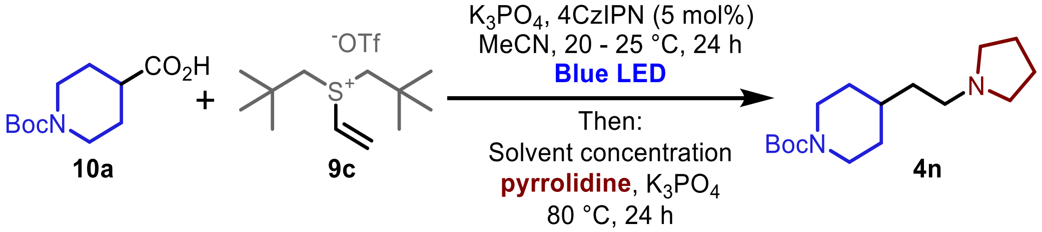

Prepared following general procedure C, using **10a** (1.0 equiv.; 0.2 mmol; 45.9 mg), dineopentyl(vinyl)sulfonium triflate **9c** (1.5 equiv.; 0.3 mmol; 105 mg), 4CzIPN (0.05 equiv.; 0.01 mmol; 7.9 mg), potassium phosphate (1.0 equiv.; 0.2 mmol; 42.4 mg). After photochemical step, the crude was transferred in a 5 mL Young tube with pyrrolidine (2.5 equiv.; 0.5 mmol; 42.3 µL) and potassium phosphate (1.5 equiv.; 0.3 mmol; 63.6 mg). The crude residue was purified by flash column chromatography (SiO_2; previously deactivated with 0.5% ammonia in water (35%) in DCM; gradient 95:5 to 9:1 DCM:methanol + 0.5% ammonia in water (35%)) to afford compound **4n** (40 mg; 71%) as a yellow solid. **R$_f$** (9:1 DCM:methanol + 0.5% ammonia in water (35%)) 0.4; **M.P.** = 158 – 160 °C; **IR** (film) ν_{max}/cm^{-1}: 2969, 2928, 2856, 1689, 1423, 1366, 1277, 1245, 1169, 768; **^{1}H NMR** ($CDCl_3$, 500 MHz) δ (ppm): 4.06 (br s, 2H), 2.85 (br s, 4H), 2.75 – 2.56 (m, 4H), 1.98 – 1.88 (m, 4H), 1.72 – 1.57 (m, 4H), 1.53 – 1.46 (m, 1H), 1.44 (s, 9H), 1.18 – 1.06 (m, 2H); **^{13}C NMR** ($CDCl_3$, 126 MHz) δ (ppm): 154.9, 79.5, 54.1, 53.8, 43.8, 34.4, 34.1, 32.1, 28.6, 23.5; **HRMS** (ESI-TOF) mass calculated for $[M+H]^+$ ($C_{16}H_{31}N_2O_2^+$) expected *m/z* 283.2380; found *m/z* 283.2386.

tert-butyl 4-(2-(phenylamino)ethyl)piperidine-1-carboxylate (**4o**)

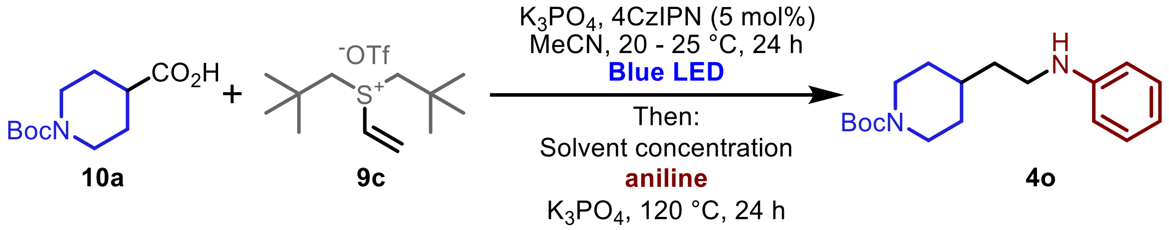

Prepared following a modification of general procedure C, using **10a** (1.0 equiv.; 0.2 mmol; 45.9 mg), dineopentyl(vinyl)sulfonium triflate **9c** (1.5 equiv.; 0.3 mmol; 105 mg), 4CzIPN (0.05 equiv.; 0.01 mmol; 7.9 mg), potassium phosphate (1.0 equiv.; 0.2 mmol; 42.4 mg). After photochemical step, the crude was transferred in a 5 mL Young tube with aniline (2.5 equiv.; 0.5 mmol; 45.6 µL) and potassium phosphate (1.5 equiv.; 0.3 mmol; 63.6 mg). The reaction was heated to 120 °C for 24 h. The crude residue was purified by flash column chromatography (SiO_2; gradient 97:3 to 95:5 DCM:ethyl acetate) to afford compound **4o** (32.3 mg; 53%) as a yellow solid. **M.P.** = 96 – 97 °C; **R$_f$** (97:3 DCM:ethyl acetate) 0.3; **IR** (film) ν_{max}/cm^{-1}: 3375, 2974, 2927, 2852, 1680, 1603, 1508, 1425, 1365, 1246, 1170, 748, 693; **^{1}H NMR** ($CDCl_3$, 500 MHz) δ (ppm): 7.21 – 7.14 (m, 2H), 6.71 (tt, *J* = 7.2, 1.1 Hz, 1H), 6.64 – 6.59 (m, 2H), 4.09 (br s, 2H), 3.19 – 3.11 (m, 2H), 2.76 – 2.61 (m, 2H), 1.69 (d, *J* = 13.1 Hz, 2H), 1.61 – 1.53 (m, 3H), 1.46 (s, 9H), 1.21 - 1.10 (m, 2H); **^{13}C NMR** ($CDCl_3$, 126 MHz) δ (ppm): 155.0, 148.4, 129.4, 117.5, 112.9, 79.4, 44.1, 41.5, 36.4, 34.0, 32.3, 28.6; **HRMS** (ESI-TOF) mass calculated for $[M+H]^+$ ($C_{18}H_{29}N_2O_2^+$) expected *m/z* 305.2224; found *m/z* 305.2223.

tert-butyl 4-(2-(((1*S*,2*S*)-1-hydroxy-1-phenylpropan-2-yl)(methyl)amino)ethyl)piperidine-1-carboxylate (**4p**)

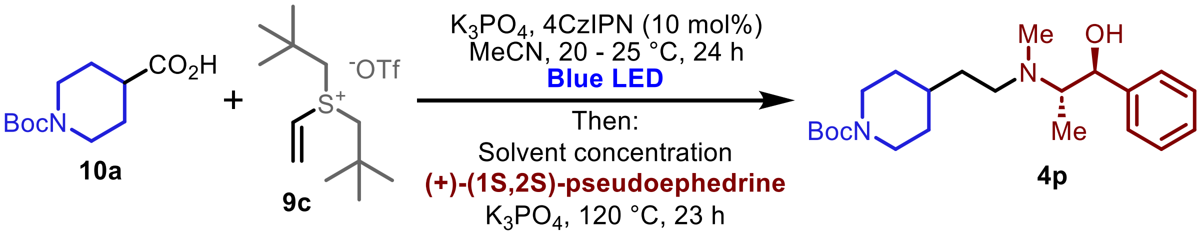

Prepared following a modification of general procedure C, using **10a** (2.0 equiv.; 0.2 mmol; 45.9 mg), dineopentyl(vinyl)sulfonium triflate **9c** (2.2 equiv.; 0.22 mmol; 77.1 mg), 4CzIPN (0.10 equiv.; 0.01 mmol; 7.9 mg), potassium phosphate (2.0 equiv.; 0.2 mmol; 42.4 mg). After photochemical step, the crude was transferred in a 5 mL Young tube with (+)-(1S,2S)-pseudoephedrine (1.0 equiv.; 0.1 mmol; 16.5 mg) and potassium phosphate (1.0 equiv.; 0.1 mmol; 21.2 mg). The reaction was heated to 120 °C for 23 h. The crude residue was purified by flash column chromatography (SiO_2; gradient 99:1 to 95:5 DCM:MeOH + 1% ammonia in

water (35%)) to afford compound **4p** (22.3 mg; 59%) as a yellow oil. **R$_f$** (97:3 DCM:MeOH + 1% ammonia in water (35%)) 0.25; **[α]$_D$^{25}** = +48 (c = 1.00 $CHCl_3$); **IR** (film) ν_{max}/cm^{-1}: 3366, 2926, 2855, 1692, 1422, 1367, 1244, 1169764, 702; **^{1}H NMR** ($CDCl_3$, 500 MHz) δ (ppm): 7.38 – 7.31 (m, 4H), 7.30 – 7.26 (m, 1H), 4.22 (d, *J* = 9.7 Hz, 1H), 4.09 (br s, 2H), 2.78 – 2.53 (m, 4H), 2.48 – 2.38 (m, 1H), 2.28 (s, 3H), 1.73 – 1.63 (m, 2H), 1.56 – 1.48 (m, 3H), 1.46 (s, 9H), 1.20 – 1.08 (m, 2H), 0.75 (d, *J* = 6.6 Hz, 3H); **^{13}C NMR** ($CDCl_3$, 126 MHz) δ (ppm): 155.0, 142.1, 128.4, 127.9, 127.5, 79.4, 74.8, 65.8, 51.0, 44.3, 36.1, 34.8, 34.1, 32.5, 32.3, 28.6, 7.5; **HRMS** (ESI-TOF) mass calculated for $[M+H]^+$ ($C_{22}H_{37}N_2O_3^+$) expected *m/z* 377.282799; found *m/z* 377.2802.

tert-butyl 2-(2-((2-hydroxyethyl)thio)ethyl)pyrrolidine-1-carboxylate (**4q**)

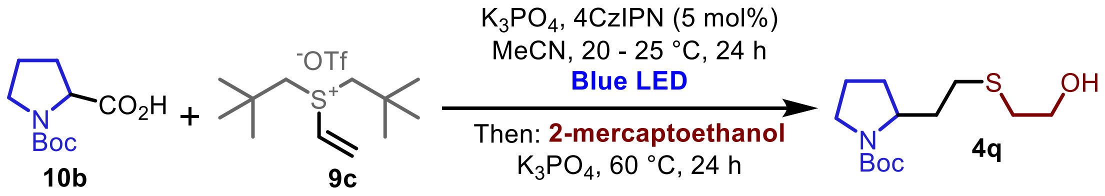

Prepared following general procedure A, using **10b** (1.0 equiv.; 0.2 mmol; 43.0 mg), dineopentyl(vinyl)sulfonium triflate **9c** (1.5 equiv.; 0.3 mmol; 105 mg), 4CzIPN (0.05 equiv.; 0.01 mmol; 7.9 mg), potassium phosphate (1.0 equiv.; 0.2 mmol; 42.4 mg) + (1.5 equiv.; 0.3 mmol; 63.6 mg) and 2-mercaptoethanol (2.5 equiv.; 0.5 mmol; 35.1 µL). The crude residue was purified by flash column chromatography (SiO_2; gradient 6:4 to 1:1 hexane:ethyl acetate) to afford compound **4q** (45.1 mg; 82%) as a pale yellow oil. **R$_f$** (6:4 hexane:ethyl acetate) 0.25; **IR** (film) ν_{max}/cm^{-1}: 3440, 2966, 2927, 1688, 1399, 1169, 1118, 774; **^{1}H NMR** ($CDCl_3$, 500 MHz) δ (ppm): 3.93 – 3.84 (m, 1H), 3.72 (t, *J* = 5.8 Hz, 2H), 3.41 – 3.33 (m, 1H), 3.33 – 3.26 (m, 1H), 2.80 – 2.68 (m, 2H), 2.59 – 2.46 (m, 2H), 2.26 – 2.18 (m, 1H), 2.03 – 1.90 (m, 2H), 1.89 – 1.77 (m, 2H), 1.66 – 1.56 (m, 2H), 1.45 (s, 9H); **^{13}C NMR** ($CDCl_3$, 126 MHz) δ (ppm): 154.9, 79.4, 60.4, 56.5, 46.4, 35.6, 34.8, 30.6, 28.7, 28.6, 23.6; **HRMS** (ESI-TOF) mass calculated for $[M+Na]^+$ ($C_{13}H_{25}NO_3SNa^+$) expected *m/z* 298.1447; found *m/z* 298.1444.

2-((2-(tetrahydrofuran-2-yl)ethyl)thio)ethan-1-ol (**4r**)

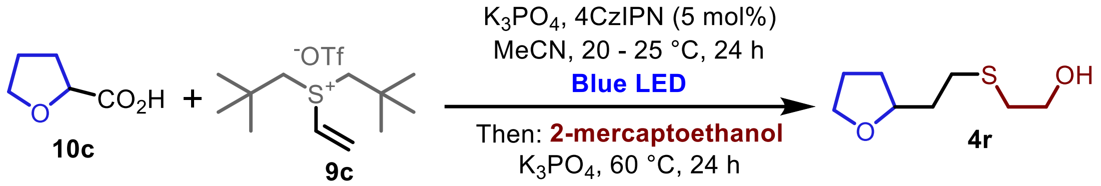

Prepared following general procedure A, using **10c** (1.0 equiv.; 0.2 mmol; 19.2 μL), dineopentyl(vinyl)sulfonium triflate **9c** (1.5 equiv.; 0.3 mmol; 105 mg), 4CzIPN (0.05 equiv.; 0.01 mmol; 7.9 mg), potassium phosphate (0.25 equiv.; 0.05 mmol; 10.6 mg) + (2.25 equiv.; 0.45 mmol; 95.4 mg) and 2-mercaptoethanol (2.5 equiv.; 0.5 mmol; 35.1 μL). The crude residue was purified by flash column chromatography (SiO_2; gradient 7:3 to 1:1 DCM:ethyl acetate) to afford compound **4r** (25.3 mg; 72%) as a transparent oil. **R$_f$** (1:1 DCM:ethyl acetate) 0.4; **IR** (film) ν_{max}/cm^{-1}: 3398, 2925, 2867, 1284, 1050, 1014; **^{1}H NMR** ($CDCl_3$, 500 MHz) δ (ppm): 3.95 – 3.88 (m, 1H), 3.87 – 3.81 (m, 1H), 3.75 – 3.68 (m, 3H), 2.73 (t, *J* = 5.9 Hz, 2H), 2.70 – 2.63 (m, 1H), 2.63 – 2.56 (m, 1H), 2.38 – 2.31 (m, 1H), 2.04 – 1.96 (m, 1H), 1.93 – 1.84 (m, 2H), 1.84 – 1.72 (m, 2H), 1.51 – 1.42 (m, 1H); **^{13}C NMR** ($CDCl_3$, 126 MHz) δ (ppm): 77.9, 67.9, 60.5, 35.9, 35.6, 31.5, 28.6, 25.8; **HRMS** (ESI-TOF) mass calculated for $[M+Na]^+$ ($C_8H_{16}O_2SNa^+$) expected *m/z* 199.0763; found *m/z* 199.0762.

2-((2-(tetrahydro-2*H*-pyran-4-yl)ethyl)thio)ethan-1-ol (**4s**)

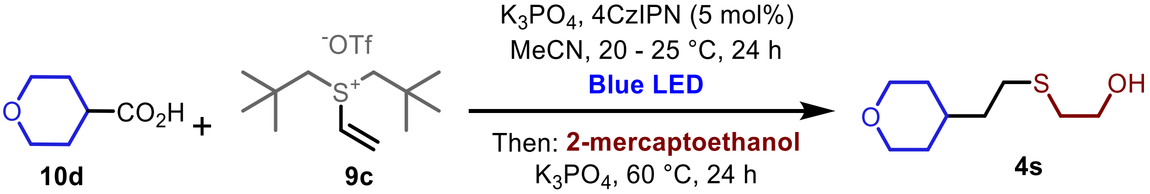

Prepared following general procedure A, using **10d** (1.0 equiv.; 0.2 mmol; 26.0 mg), dineopentyl(vinyl)sulfonium triflate **9c** (1.5 equiv.; 0.3 mmol; 105 mg), 4CzIPN (0.05 equiv.; 0.01 mmol; 7.9 mg), potassium phosphate (1.0 equiv.; 0.2 mmol; 42.4 mg) + (1.5 equiv.; 0.3 mmol; 63.6 mg) and 2-mercaptoethanol (2.5 equiv.; 0.5 mmol; 35.1 μL). The crude residue was purified by flash column chromatography (SiO_2; gradient 6:4 to 1:1 hexane:ethyl acetate) to afford compound **4s** (26 mg; 68%) as a transparent oil. **R$_f$** (3:2 hexane:ethyl acetate) 0.3; **IR** (film) ν_{max}/cm^{-1}: 3416, 2921, 2847, 1444, 1294, 1089, 857; **^{1}H NMR** ($CDCl_3$, 500 MHz) δ (ppm): 3.97 – 3.92 (m, 2H), 3.76 – 3.68 (m, 2H), 3.37 (td, *J* = 11.8, 2.0 Hz, 2H), 2.73 (t, *J* = 6.0 Hz, 2H), 2.59 – 2.52 (m, 2H), 2.18 (br s, 1H), 1.69 – 1.51 (m, 5H), 1.34 – 1.22 (m, 2H); **^{13}C NMR** ($CDCl_3$, 126 MHz) δ (ppm): 68.1, 60.4, 36.8, 35.5, 34.2, 32.9, 28.8; **HRMS** (ESI-TOF) mass calculated for $[M+Na]^+$ ($C_9H_{18}O_2SNa^+$) expected *m/z* 213.0920; found *m/z* 213.0914.

2-((2-cyclododecylethyl)thio)ethan-1-ol (**4t**)

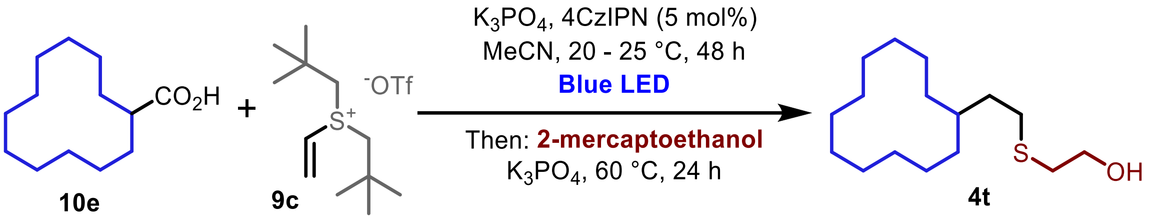

Prepared following general procedure A, irradiated for 48 h, using **10e** (1.0 equiv.; 0.2 mmol; 42.5 mg), dineopentyl(vinyl)sulfonium triflate **9c** (1.5 equiv.; 0.3 mmol; 105 mg), 4CzIPN (0.05 equiv.; 0.01 mmol; 7.9 mg), potassium phosphate (1.0 equiv.; 0.2 mmol; 42.4 mg) + (1.5 equiv.; 0.3 mmol; 63.6 mg) and 2-mercaptoethanol (2.5 equiv.; 0.5 mmol; 35.1 µL). The crude residue was purified by flash column chromatography (SiO_2; gradient 99:1 to 98:2 DCM:ethyl acetate) to afford compound **4t** (28 mg; 51%) as a transparent oil. **R_f** (97:3 hexane:ethyl acetate) 0.3; **IR** (film) ν_{max}/cm^{-1}: 3366, 2928, 2860, 1470, 1445, 1046, 1012, 719; **^{1}H NMR** ($CDCl_3$, 500 MHz) δ (ppm): 3.72 (q, *J* = 6.0 Hz, 2H), 2.73 (t, *J* = 6.0 Hz, 2H), 2.57 – 2.48 (m, 2H), 2.18 (td, *J* = 6.2, 2.1 Hz, 1H), 1.55 – 1.45 (m, 3H), 1.39 – 1.19 (m, 22H); **^{13}C NMR** ($CDCl_3$, 126 MHz) δ (ppm): 60.2, 35.5, 35.1, 33.6, 29.8, 29.0, 24.9, 24.3, 23.5, 23.4, 21.8; **HRMS** (ESI-TOF) mass calculated for $[M+Na]^+$ ($C_{16}H_{32}SONa^+$) expected *m/z* 295.2066; found *m/z* 295.2062.

tert-butyl 2-(2-((2-hydroxyethyl)thio)ethyl)azetidine-1-carboxylate (**4u**)

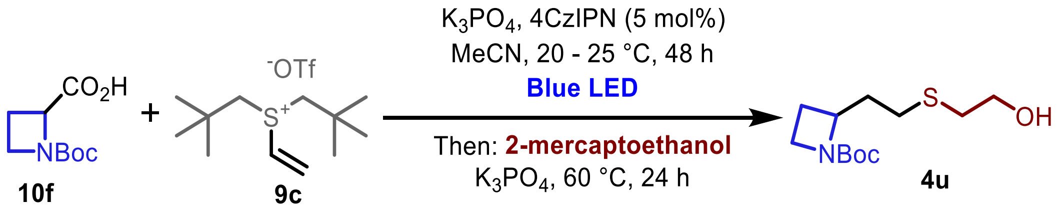

Prepared following general procedure A, using **10f** (1.0 equiv.; 0.2 mmol; 42.5 mg), dineopentyl(vinyl)sulfonium triflate **9c** (1.5 equiv.; 0.3 mmol; 105 mg), 4CzIPN (0.05 equiv.; 0.01 mmol; 7.9 mg), potassium phosphate (1.0 equiv.; 0.2 mmol; 42.4 mg) + (1.5 equiv.; 0.3 mmol; 63.6 mg) and 2-mercaptoethanol (2.5 equiv.; 0.5 mmol; 35.1 µL). The crude residue was purified by flash column chromatography (SiO_2; gradient 85:15 to 7:3 DCM:ethyl acetate) to afford compound **4u** (38 mg; 73%) as a transparent oil. **R_f** (7:3 DCM:ethyl acetate) 0.2; **IR** (film) ν_{max}/cm^{-1}: 3436, 2972, 2928, 2892, 1697, 1676, 1393, 1366, 1290, 1141, 1047, 863, 775; **^{1}H NMR** ($CDCl_3$, 500 MHz) δ (ppm): 4.34 – 4.26 (m, 1H), 3.87 – 3.75 (m, 2H), 3.73 (t, *J* = 5.9 Hz, 2H), 2.73 (t, *J* = 6.0 Hz, 2H), 2.59 (t, *J* = 7.6 Hz, 2H), 2.34 – 2.25 (m, 1H), 2.20 – 2.09 (m, 2H), 1.93 – 1.79 (m, 2H), 1.43 (s, 9H); **^{13}C NMR** ($CDCl_3$, 126 MHz) δ (ppm): 156.9, 79.6,

61.1, 60.4, 46.7, 36.0, 35.4, 28.6, 27.3, 22.0; **HRMS** (ESI-TOF) mass calculated for $[M+Na]^+$ ($C_{12}H_{23}NO_3SNa^+$) expected *m/z* 284.1291; found *m/z* 284.1296.

2-((2-((1*s*,3*s*)-adamantan-1-yl)ethyl)thio)ethan-1-ol (**4v**)

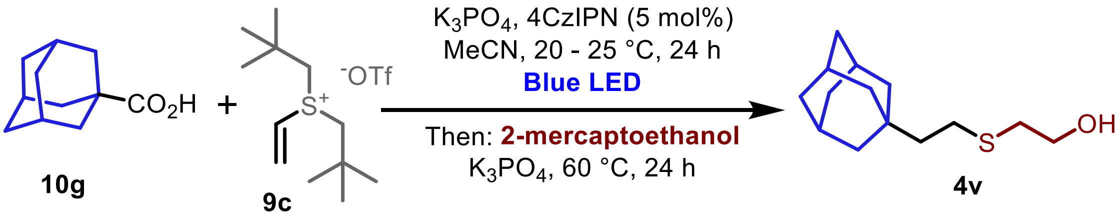

Prepared following general procedure A, using **10g** (1.0 equiv.; 0.2 mmol; 36.0 mg), dineopentyl(vinyl)sulfonium triflate **9c** (1.5 equiv.; 0.3 mmol; 105 mg), 4CzIPN (0.05 equiv.; 0.01 mmol; 7.9 mg), potassium phosphate (1.0 equiv.; 0.2 mmol; 42.4 mg) + (1.5 equiv.; 0.3 mmol; 63.6 mg) and 2-mercaptoethanol (2.5 equiv.; 0.5 mmol; 35.1 µL). The crude residue was purified by flash column chromatography (SiO_2; gradient 9:1 to 8:2 hexane:ethyl acetate). The compound was purified a second time by flash column chromatography (SiO_2; gradient 95:5 to 9:1 toluene:Et_2O) to afford compound **4v** (37.3 mg; 77%) as a transparent oil. $\mathbf{R_f}$ (8:2 hexane:ethyl acetate) 0.35; **IR** (film) ν_{max}/cm^{-1}: 3386, 2901, 2845, 1448, 1221, 1048, 772; **^{1}H NMR** ($CDCl_3$, 500 MHz) δ (ppm): 3.71 (q, *J* = 6.0 Hz, 2H), 2.73 (t, *J* = 5.9 Hz, 2H), 2.51 – 2.44 (m, 2H), 2.21 – 2.15 (m, 1H), 1.98 – 1.92 (m, 3H), 1.74 – 1.67 (m, 3H), 1.65 – 1.59 (m, 3H), 1.48 (d, *J* = 2.8 Hz, 6H), 1.39 – 1.33 (m, 2H); **^{13}C NMR** ($CDCl_3$, 126 MHz) δ (ppm): 60.2, 44.6, 42.3, 37.2, 35.5, 32.9, 28.7, 25.7; **HRMS** (ESI-TOF) mass calculated for $[M+Na]^+$ ($C_{14}H_{24}OSNa^+$) expected *m/z* 263.1440; found *m/z* 263.1447.

2-(2-((2-hydroxyethyl)thio)ethyl)-2,5,7,8-tetramethylchroman-6-ol (**4w**)

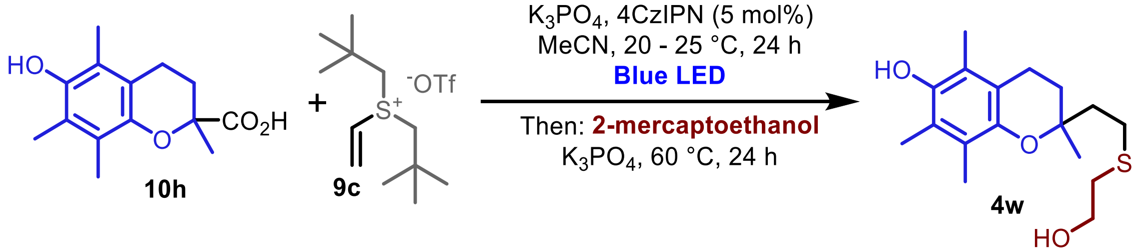

Prepared following general procedure A, using **10h** (1.0 equiv.; 0.2 mmol; 50.0 mg), dineopentyl(vinyl)sulfonium triflate **9c** (1.5 equiv.; 0.3 mmol; 105 mg), 4CzIPN (0.05 equiv.; 0.01 mmol; 7.9 mg), potassium phosphate (1.0 equiv.; 0.2 mmol; 42.4 mg) + (1.5 equiv.; 0.3 mmol; 63.6 mg) and 2-mercaptoethanol (2.5 equiv.; 0.5 mmol; 35.1 µL). The crude residue was purified by flash column chromatography (SiO_2; gradient 8:2 to 7:3 hexane:acetone) to afford compound **4w** (46 mg; 74%) as a yellow solid. $\mathbf{R_f}$ (7:3 hexane:acetone) 0.2; **M.P.** = 32

– 33 °C; **IR** (film) ν_{max}/cm^{-1}: 3380, 2926, 2868, 1454, 1422, 1379, 1343, 1297, 1166, 1086, 1058, 1008, 925, 856, 608, 575, 549, 519, 477, 447; **^{1}H NMR** ($CDCl_3$, 500 MHz) δ (ppm): 4.28 – 4.22 (m, 1H), 3.70 (q, *J* = 5.9 Hz, 2H), 2.73 – 2.59 (m, 6H), 2.16 (s, 3H), 2.18 – 2.08 (m, 6H), 1.98 – 1.90 (m, 1H), 1.86 – 1.74 (m, 3H), 1.24 (s, 3H); **^{13}C NMR** ($CDCl_3$, 126 MHz) δ (ppm): 145.2, 145.0, 122.7, 121.3, 118.7, 117.2, 74.0, 60.2, 40.1, 35.5, 31.8, 25.9, 23.7, 20.7, 12.4, 12.0, 11.4; **HRMS** (ESI-TOF) mass calculated for $[M+Na]^+$ ($C_{17}H_{26}O_3SNa^+$) expected *m/z* 333.1495; found *m/z* 333.1489.

4-chloro-*N*-(4-((4-((2-hydroxyethyl)thio)-2-methylbutan-2-yl)oxy)phenethyl)benzamide (**4x**)

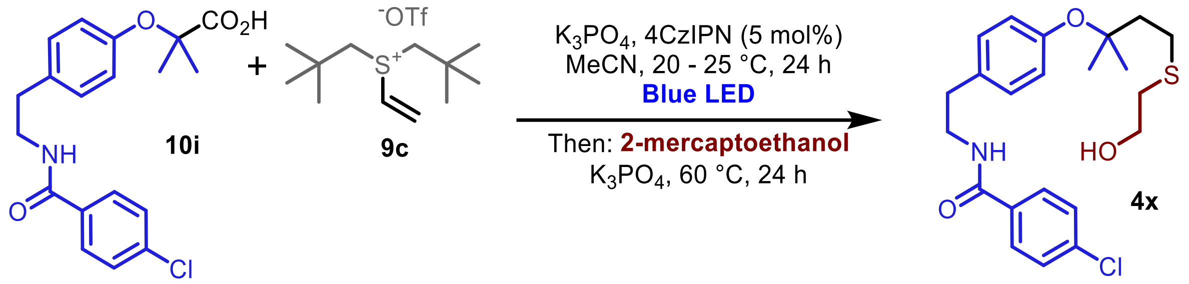

Prepared following general procedure A, using **10i** (1.0 equiv.; 0.2 mmol; 50 mg), dineopentyl(vinyl)sulfonium triflate **9c** (1.5 equiv.; 0.3 mmol; 105 mg), 4CzIPN (0.05 equiv.; 0.01 mmol; 7.9 mg), potassium phosphate (1.0 equiv.; 0.2 mmol; 42.4 mg) + (1.5 equiv.; 0.3 mmol; 63.6 mg) and 2-mercaptoethanol (2.5 equiv.; 0.5 mmol; 35.1 µL). The crude residue was purified by flash column chromatography (SiO_2; gradient 1:1 to 4:6 hexane:ethyl acetate) to afford compound **4x** (67 mg; 79%) as a yellow solid. **R$_f$** (4:6 hexane:ethyl acetate) 0.2; **M.P.** = 37 – 38 °C; **IR** (film) ν_{max}/cm^{-1}: 3317, 3086, 2973, 2930, 2872, 1638, 1597, 1544, 1506, 1487, 1439, 1384, 1367, 1316, 1233, 1193, 1162, 1108, 1094, 1046, 1015, 895, 847, 758, 525; **^{1}H NMR** ($CDCl_3$, 500 MHz) δ (ppm): 7.66 – 7.57 (m, 2H), 7.44 – 7.34 (m, 2H), 7.16 – 7.08 (m, 2H), 6.97 – 6.87 (m, 2H), 6.15 – 6.04 (m, 1H), 3.76 – 3.61 (m, 4H), 2.88 (t, *J* = 6.9 Hz, 2H), 2.79 – 2.67 (m, 4H), 2.18 (t, *J* = 6.4 Hz, 1H), 1.98 – 1.88 (m, 2H), 1.29 (s, 6H); **^{13}C NMR** ($CDCl_3$, 126 MHz) δ (ppm): 166.5, 153.9, 137.8, 133.8, 133.1, 129.5, 129.0, 128.4, 124.1, 79.8, 60.4, 42.4, 41.4, 35.5, 35.0, 26.8, 26.4; **HRMS** (ESI-TOF) mass calculated for $[M+Na]^+$ ($C_{22}H_{28}NO_3SClNa^+$) expected *m/z* 444.1371; found *m/z* 444.1369.

2-((6-(2,5-dimethylphenoxy)-3,3-dimethylhexyl)thio)ethan-1-ol (**4y**)

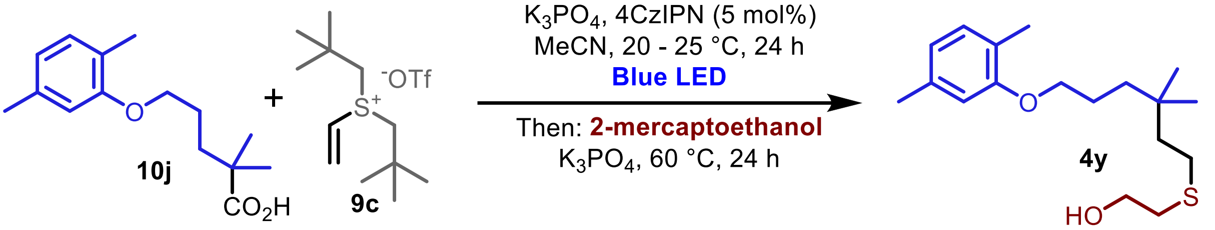

Prepared following general procedure A, using **10j** (1.0 equiv.; 0.2 mmol; 50.1 mg), dineopentyl(vinyl)sulfonium triflate **9c** (1.5 equiv.; 0.3 mmol; 105 mg), 4CzIPN (0.05 equiv.; 0.01 mmol; 7.9 mg), potassium phosphate (1.0 equiv.; 0.2 mmol; 42.4 mg) + (1.5 equiv.; 0.3 mmol; 63.6 mg) and 2-mercaptoethanol (2.5 equiv.; 0.5 mmol; 35.1 µL). The crude residue was purified by flash column chromatography (SiO_2; gradient 9:1 to 85:15 hexane:ethyl acetate) to afford compound **4y** (47.4 mg; 84%) as a transparent oil. **R**$_f$ (8:2 hexane:ethyl acetate) 0.35; **IR** (film) ν_{max}/cm^{-1}: 3402, 2953, 2867, 1615, 1585, 1509, 1470, 1265, 1157, 1130, 1049, 804; **^{1}H NMR** ($CDCl_3$, 500 MHz) δ (ppm): 7.01 (d, *J* = 7.5 Hz, 1H), 6.66 (d, *J* = 7.5 Hz, 1H), 6.62 (s, 1H), 3.92 (t, *J* = 6.3 Hz, 2H), 3.72 (t, *J* = 5.9 Hz, 2H), 2.74 (t, *J* = 5.9 Hz, 2H), 2.53 – 2.46 (m, 2H), 2.31 (s, 3H), 2.18 (s, 3H), 1.78 – 1.70 (m, 2H), 1.57 – 1.51 (m, 2H), 1.42 – 1.35 (m, 2H), 0.92 (s, 6H); **^{13}C NMR** ($CDCl_3$, 126 MHz) δ (ppm): 157.1, 136.6, 130.4, 123.7, 120.8, 112.1, 68.5, 60.3, 42.1, 38.0, 35.5, 33.1, 27.1, 26.8, 24.4, 21.5, 15.9; **HRMS** (ESI-TOF) mass calculated for $[M+Na]^+$ ($C_{18}H_{30}O_2SNa^+$) expected *m/z* 333.1859; found *m/z* 333.1862.

(3*S*,4a*R*,6a*R*,6b*S*,8a*R*,12a*R*,14a*R*,14b*R*)-8a-(2-((2-hydroxyethyl)thio)ethyl)-4,4,6a,6b,11,11,14b-heptamethyl-1,2,3,4,4a,5,6,6a,6b,7,8,8a,9,10,11,12,12a,14,14a,14b-icosahydropicen-3-ol (**4z**)

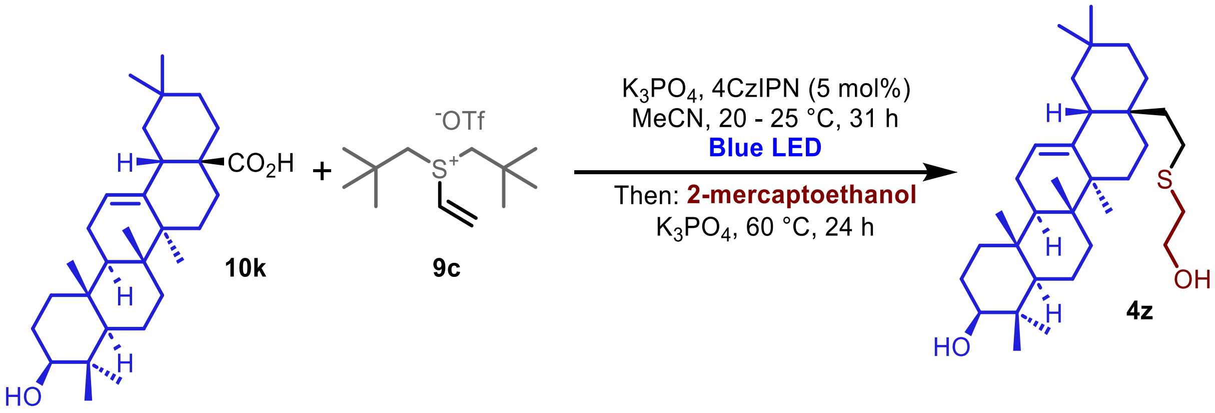

Prepared following general procedure A, irradiating for 31 h, using **10k** (1.0 equiv.; 0.2 mmol; 91.3 mg), dineopentyl(vinyl)sulfonium triflate **9c** (1.5 equiv.; 0.3 mmol; 105 mg), 4CzIPN

(0.05 equiv.; 0.01 mmol; 7.9 mg), potassium phosphate (1.0 equiv.; 0.2 mmol; 42.4 mg) + (1.5 equiv.; 0.3 mmol; 63.6 mg) and 2-mercaptoethanol (2.5 equiv.; 0.5 mmol; 35.1 µL). The crude residue was purified by flash column chromatography (SiO_2; gradient 95:5 to 8:2 DCM:ethyl acetate) to afford compound **4z** (57.9 mg; 56%; d.r. >20:1) as a white solid. The stereochemistry is assigned by assuming analogous mechanistic pathway to a Giese reaction reported for the same substrate.[12] **R_f** (9:1 DCM:ethyl acetate) 0.2; **$[\alpha]_D^{25}$** = +40 (c = 1.00 $CHCl_3$); **M.P.** = 121 – 122 °C; **IR** (film) ν_{max}/cm^{-1}: 3373, 2923, 2867, 1462, 1383 , 1284, 1218, 1137, 1036, 998, 820, 755, 662, 600; **^{1}H NMR** ($CDCl_3$, 500 MHz) δ (ppm): 5.20 (t, *J* = 3.7 Hz, 1H), 3.70 (t, *J* = 5.9 Hz, 2H), 3.26 – 3.13 (m, 1H), 2.72 (t, *J* = 5.9 Hz, 2H), 2.50 – 2.33 (m, 2H), 1.98 – 1.90 (m, 2H), 1.89 – 1.80 (m, 3H), 1.78 – 1.67 (m, 3H), 1.65 – 1.59 (m, 3H), 1.58 – 1.51 (m, 3H), 1.52 – 1.48 (m, 1H), 1.41 – 1.37 (m, 1H), 1.36 – 1.33 (m, 1H), 1.32 – 1.28 (m, 2H), 1.28 – 1.22 (m, 1H), 1.22 – 1.17 (m, 1H), 1.16 – 1.10 (m, 4H), 1.06 – 1.02 (m, 1H), 1.01 – 0.91 (m, 12H), 0.87 (s, 3H), 0.86 (s, 3H), 0.79 (s, 3H), 0.75 – 0.71 (m, 1H); **^{13}C NMR** ($CDCl_3$, 126 MHz) δ (ppm): 144.4, 122.7, 79.1, 60.1, 55.3, 47.7, 47.1, 46.7, 41.8, 40.3, 40.0, 38.9, 38.7, 37.1, 35.4, 35.3, 34.5, 33.4, 33.2, 32.7, 31.1, 28.2, 27.4, 26.2, 25.9, 25.5, 23.7, 23.7, 23.3, 18.5, 17.0, 15.7, 15.7; **HRMS** (ESI-TOF) mass calculated for $[M+Na]^+$ ($C_{33}H_{56}O_2SNa^+$) expected *m/z* 539.3893; found *m/z* 539.3893.

(4a*R*,6a*S*,6b*R*,8a*R*,10*S*,12a*S*,12b*R*,14b*R*)-10-hydroxy-2-(2-((2-hydroxyethyl)thio)ethyl)-2,4a,6a,6b,9,9,12a-heptamethyl-1,3,4,4a,5,6,6a,6b,7,8,8a,9,10,11,12,12a,12b,14b-octadecahydropicen-13(2*H*)-one (**4aa**)

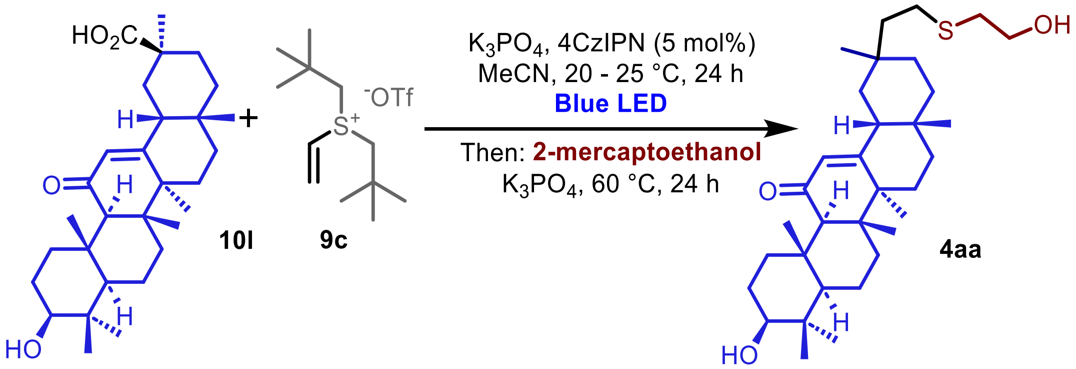

Prepared following general procedure A, using **10l** (1.0 equiv.; 0.2 mmol; 94.2 mg), dineopentyl(vinyl)sulfonium triflate **9c** (1.5 equiv.; 0.3 mmol; 105 mg), 4CzIPN (0.05 equiv.; 0.01 mmol; 7.9 mg), potassium phosphate (1.0 equiv.; 0.2 mmol; 42.4 mg) + (1.5 equiv.; 0.3 mmol; 63.6 mg) and 2-mercaptoethanol (2.5 equiv.; 0.5 mmol; 35.1 µL). The crude residue was purified by flash column chromatography (SiO_2; gradient 97.5:2.5 to 92.5:7.5 DCM:methanol). The compound was purified a second time by flash column chromatography

(SiO_2; gradient 75:25 to 1:1 DCM:ethyl acetate) to afford compound **4aa** (87.0 mg; 82%; d.r. 2.3:1) as a white solid. **R_f** (1:1 DCM:ethyl acetate) 0.4; **M.P.** = 113 – 115 °C; **$[\alpha]_D^{25}$** = +72 (c = 1.00 $CHCl_3$); **IR** (film) ν_{max}/cm^{-1}: 3394, 2925, 2865, 1652, 1460, 1386, 1208, 1043, 994, 755; **^{1}H NMR** ($CDCl_3$, 500 MHz) δ (ppm): 5.57 (s, 1H), 3.76 – 3.68 (m, 2H), 3.25 – 3.18 (m, 1H), 2.81 – 2.69 (m, 3H), 2.53 – 2.38 (m, 2H), 2.32 (s, 1H), 2.15 – 2.06 (m, 1H), 2.06 – 1.98 (m, 1H), 1.85 – 1.77 (m, 3H), 1.74 – 1.52 (m, 6H), 1.49 – 1.39 (m, 4H), 1.36 – 1.28 (m, 5H), 1.23 – 1.07 (m, 9H), 1.01 – 0.93 (m, 5H), 0.89 (s, 2H), 0.87 – 0.83 (m, 4H), 0.80 (s, 3H), 0.72 – 0.66 (m, 1H); **^{13}C NMR** ($CDCl_3$, 126 MHz) diastereomeric mixture δ (ppm) : 200.4, 170.2, 128.4, 78.9, 61.9, 60.6, 60.3, 55.1, 47.2, 47.1, 46.1, 45.6, 45.5, 43.5, 43.5, 43.4, 43.2, 39.3, 37.2, 36.0, 35.6, 35.5, 35.4, 34.2, 33.9, 33.0, 32.9, 32.8, 32.6, 32.4, 29.3, 28.8, 28.2, 27.4, 26.8, 26.6, 26.5, 26.2, 23.6, 23.5, 20.8, 18.9, 17.6, 16.5, 15.7; **HRMS** (ESI-TOF) mass calculated for $[M+H]^+$ ($C_{33}H_{55}O_3S^+$) expected *m/z* 531.3866; found *m/z* 531.3840.

4.7. Reaction with *in-situ* generation of the vinyl sulfonium

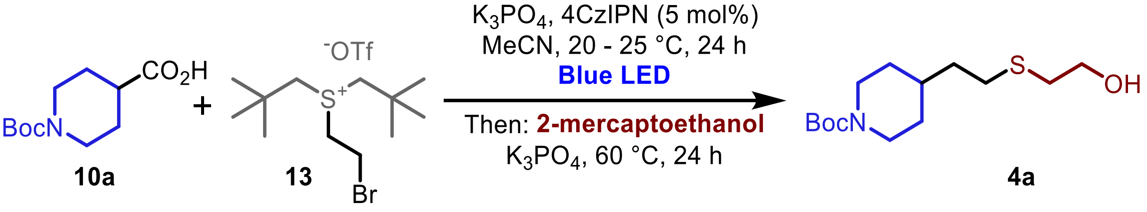

Prepared following general procedure A, using **10a** (1.0 equiv.; 0.2 mmol; 45.9 mg), (2-bromoethyl)dineopentylsulfonium triflate **13** (1.5 equiv.; 0.3 mmol; 129 mg), 4CzIPN (0.05 equiv.; 0.01 mmol; 7.9 mg), potassium phosphate (2.5 equiv.; 0.5 mmol; 106 mg) + (1.5 equiv.; 0.3 mmol; 63.6 mg) and 2-mercaptoethanol (2.5 equiv.; 0.5 mmol; 35.1 µL). The crude residue was purified by flash column chromatography (SiO_2; gradient 9:1 to 6:4 DCM:ethyl acetate) to afford compound **4a** (38.8 mg; 67%) as a pale yellow oil. See compound **4a** for full characterization.

5. References

[1] Fuentes, J. A.; Carpenter, I.; Kann, N.; Clarke, M. L. *Chem. Commun*. **2013**, *49*, 10245–10247.

[2] Chu, W.; Zhang, J.; Zeng, C.; Rothfuss, J.; Tu, Z.; Chu, Y.; Reichert, D. E.; Welch, M. J.; Mach, R. H. *J. Med. Chem.* **2005**, *48*, 7637–7647.

[3] Wang, Y.; Zhang, W.; Colandrea, V. J.; Jimenez, L. S. *Tetrahedron* **1999**, *55*, 10659–10672.

[4] Yar, M.; McGarrigle, E. M.; Aggarwal, V. K. *Angew. Chem. Int. Ed*. **2008**, *20*, 3784–3786.

[5] Huang, H.; Yu, C.; Zhang, Y.; Zhang, Y.; Mariano, P. S.; Wang, W. *J. Am. Chem. Soc.* **2017**, *139*, 9799–9802.

[6] Garreau, M.; Le Vaillant, F.; Waser, J. *Angew. Chem. Int. Ed.* **2019**, *58*, 8182–8186.

[7] Speckmeier, E.; Fischer, T. G.; Zeitler, K. *J. Am. Chem. Soc.* **2018**, *45*, 15353–15365.

[8] Landini, D.; Rolla, F. *Synthesis* **1974**, *8*, 565–566.

[9] Freeman, F.; Angeletakis, C. N.; Maricich, T. J. *Org. Mag. Reson.* **1981**, *17*, 53–58.

[10] Sambol, M.; Benčić, P.; Erben, A.; Matković, M.; Mihaljević, B.; Piantanida, I.; Kralj, M.; Basarić, N. *Molecules* **2021**, *26*, 3355.

[11] Perry, I. B.; Brewer, T.; Sarver, P.J.; Schultz, D. M.; DiRocco, D.A.; MacMillan, D.W.C. *Nature* **2018**, *560*, 70-75.

[12] Qin, T.; Malins, L. R.; Edwards, J. T.; Merchant, R. R.; Novak, A. J. E.; Zhong, J. Z.; Mills, R. B.; Yan, M.; Yuan, C.; Eastgate, M. D.; Baran, P. S. *Angew. Chem. Int. Ed.* **2017**, *56*, 260–265.

6. NMR Spectra

Dineopentyl sulfide

^{1}H-NMR ($CDCl_3$, 500 MHz)

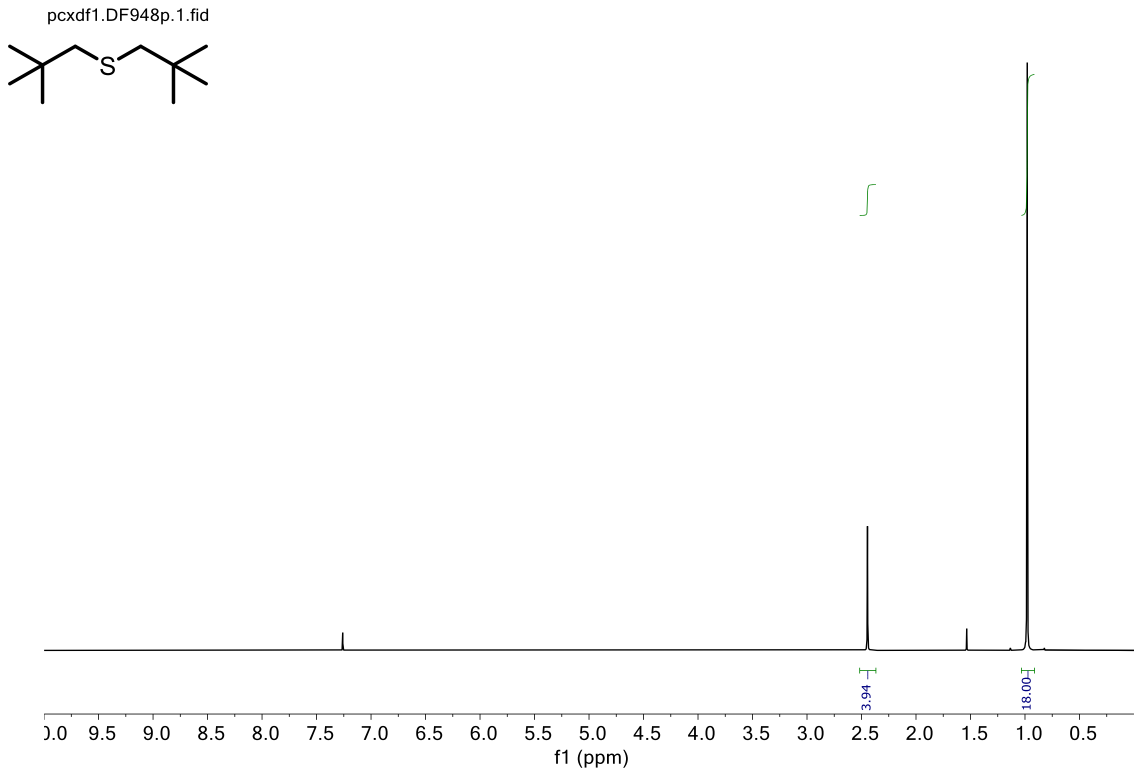

^{13}C-NMR ($CDCl_3$, 126 MHz)

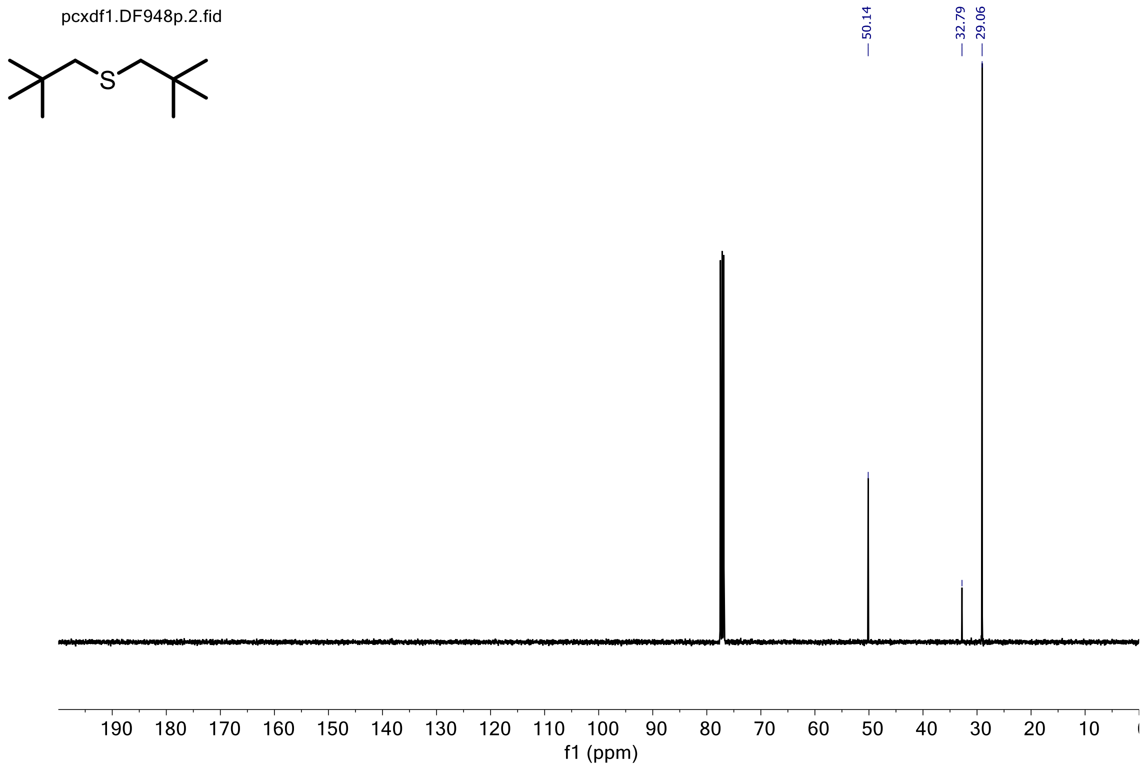

(2-bromoethyl)dineopentylsulfonium triflate (13)

^{1}H-NMR ($CDCl_3$, 500 MHz)

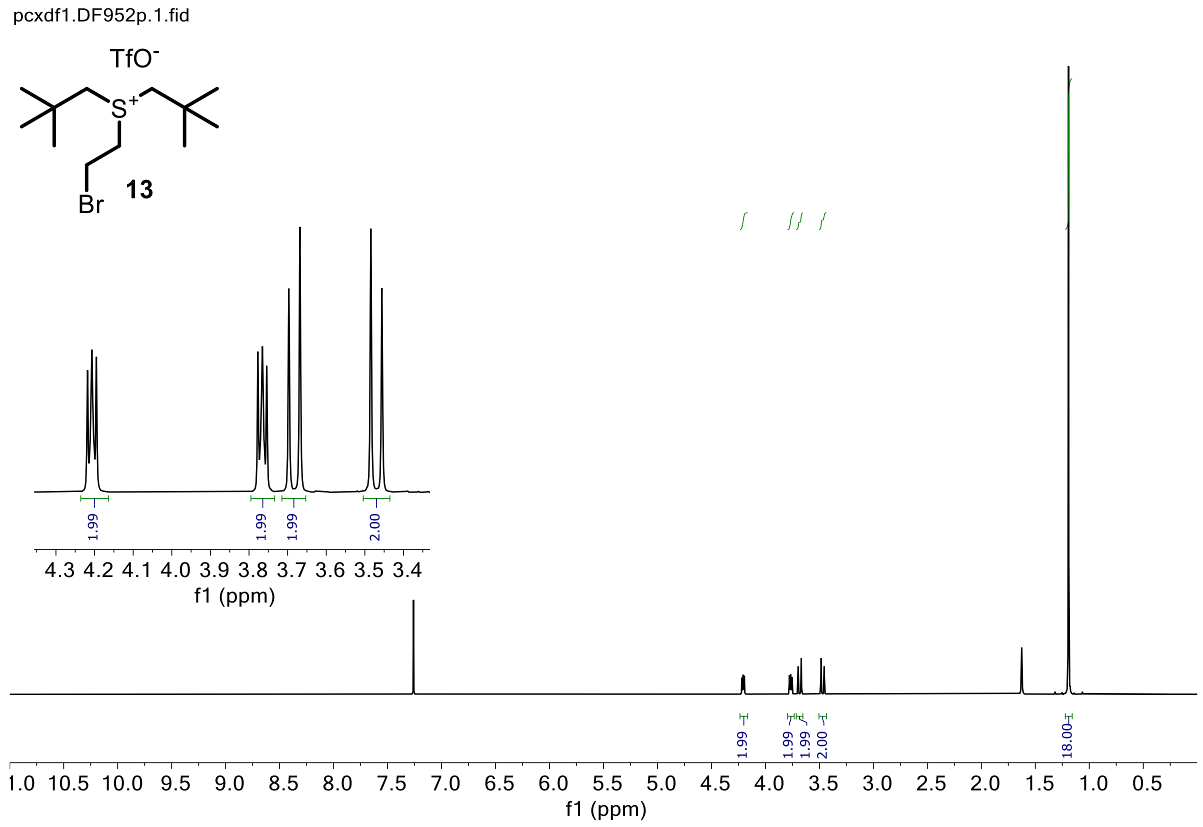

^{13}C-NMR ($CDCl_3$, 126 MHz)

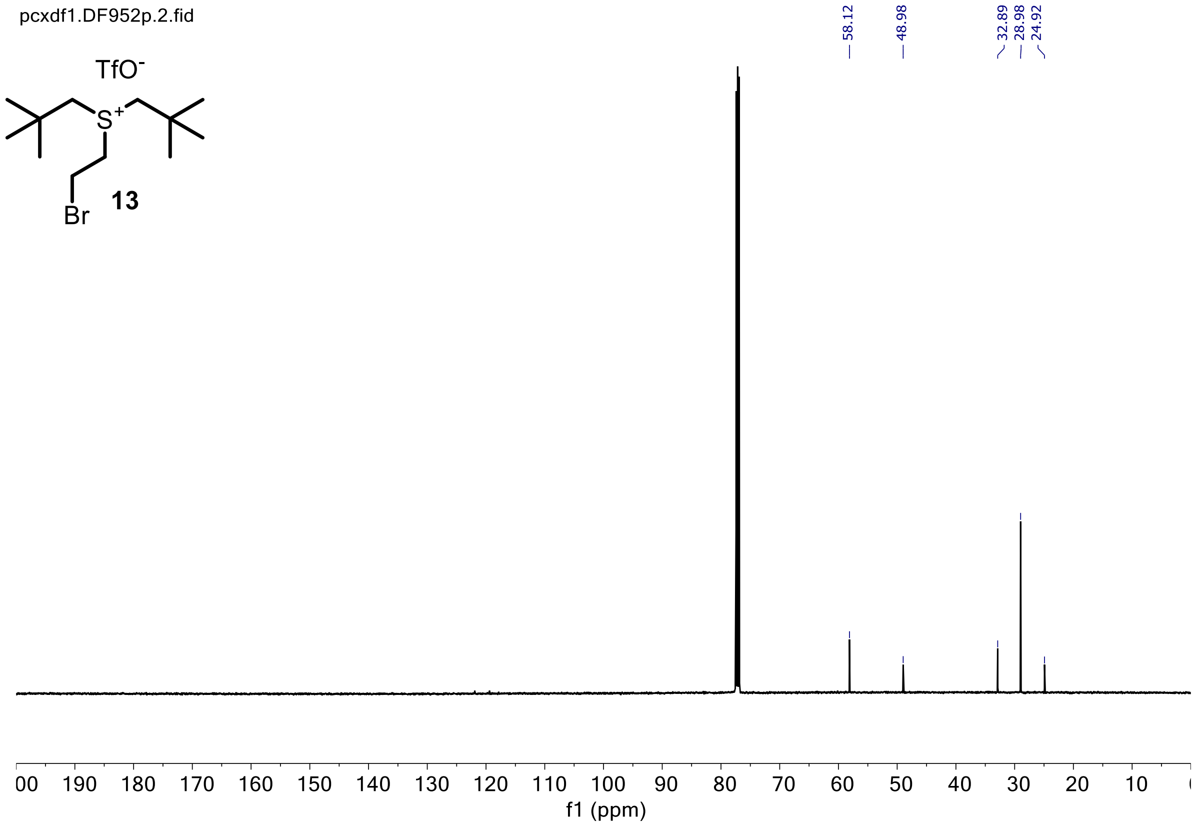

^{19}F-NMR (CDCl3, 376 MHz)

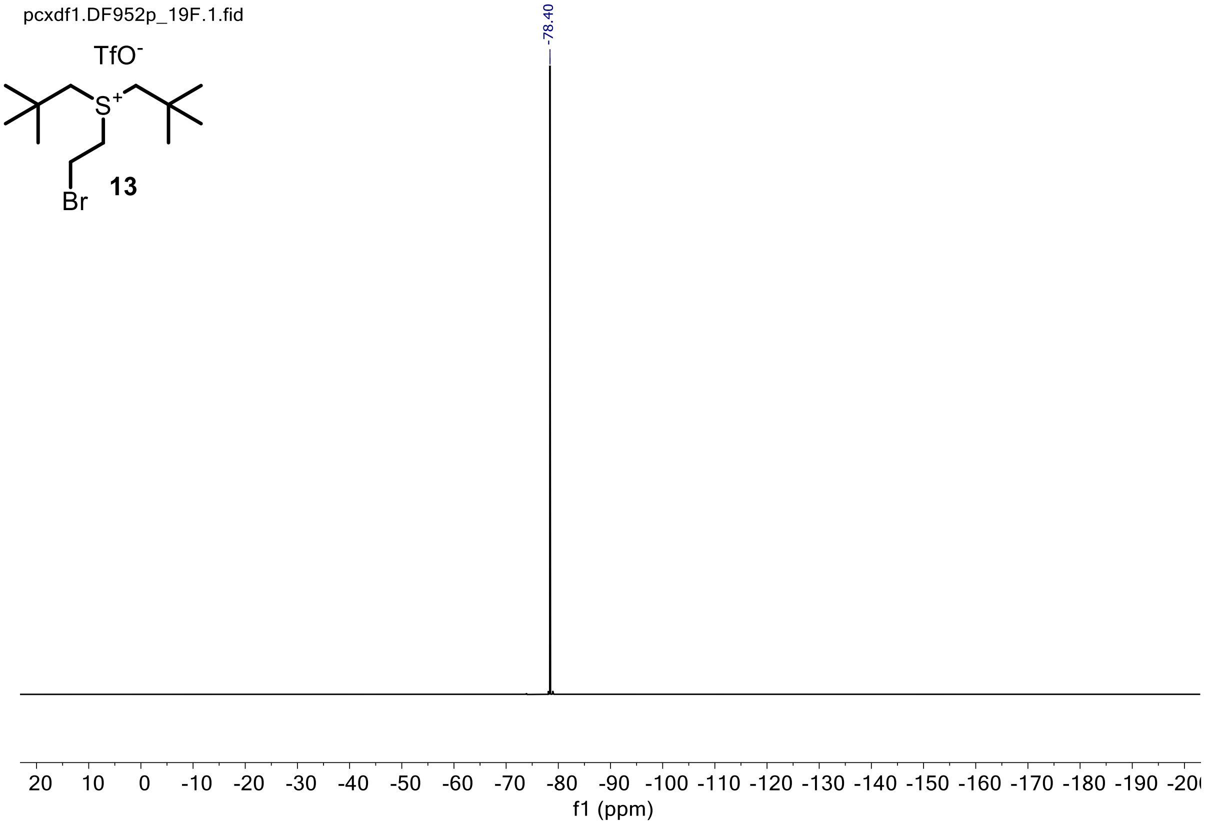

Dineopentyl(vinyl)sulfonium triflate (9c)

^{1}H-NMR ($CDCl_3$, 500 MHz)

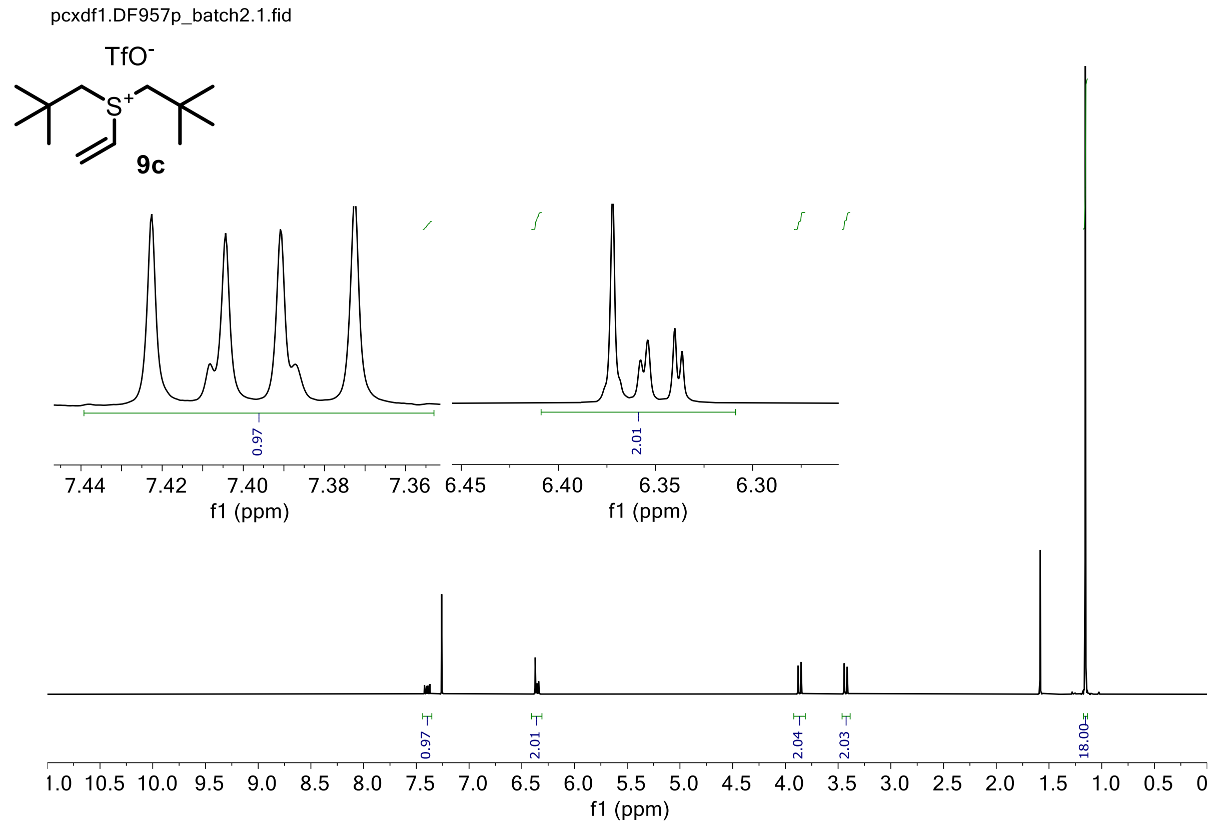

^{1}H-NMR (DMSO-d_6, 500 MHz)

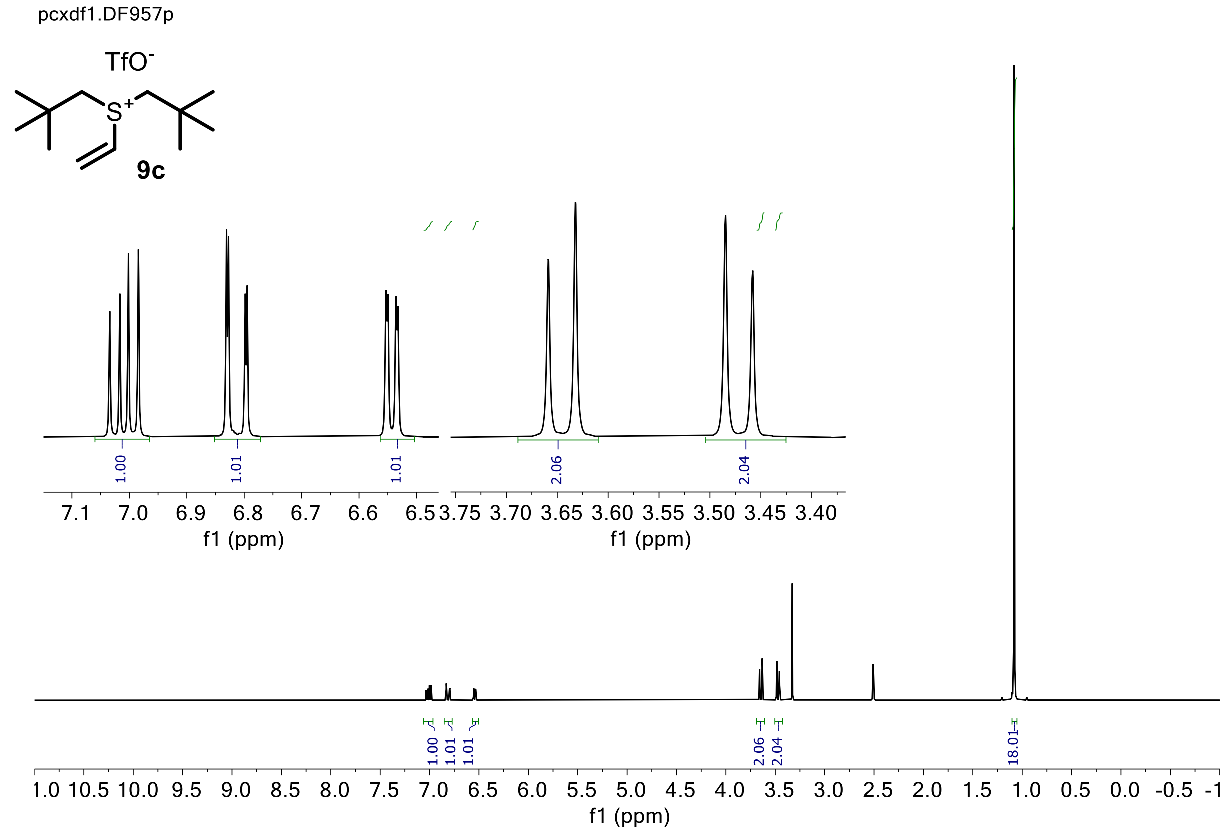

^{13}C-NMR (CDCl$_3$, 126 MHz)

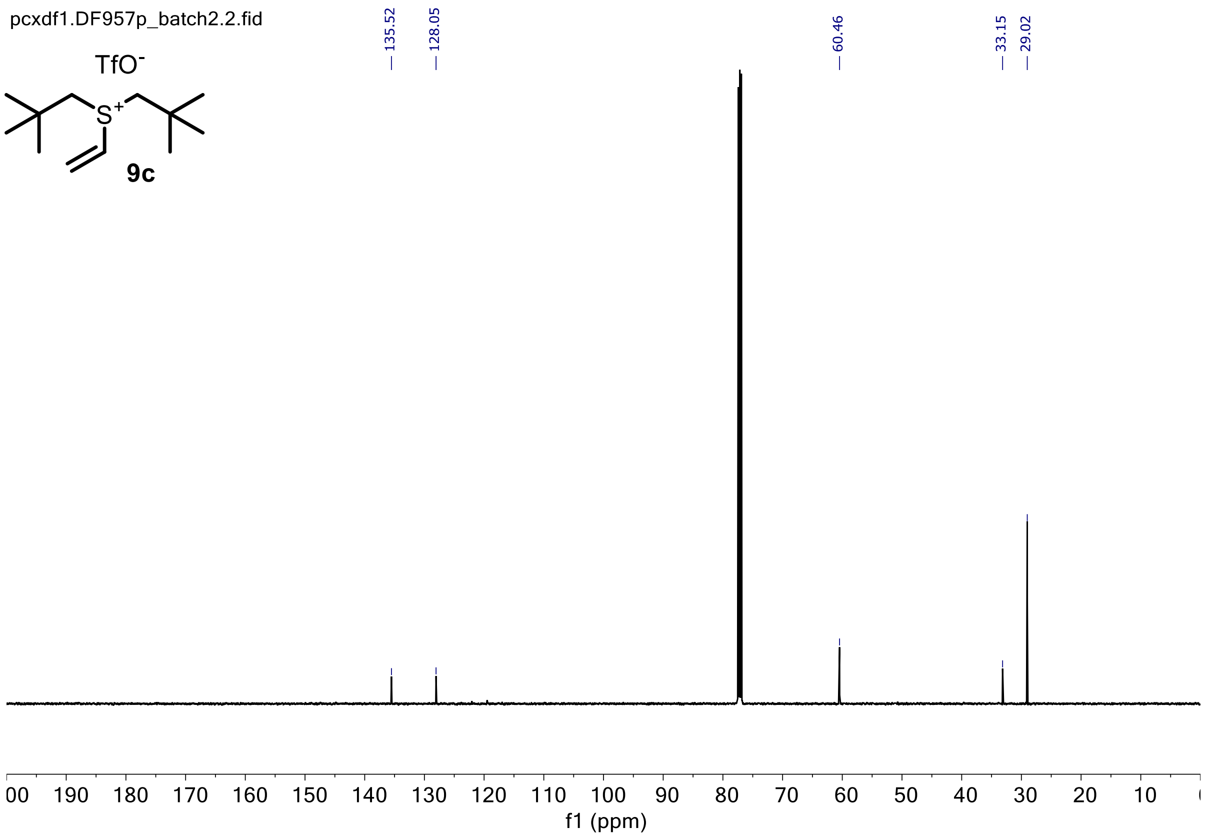

^{19}F-NMR (CDCl3, 376 MHz)

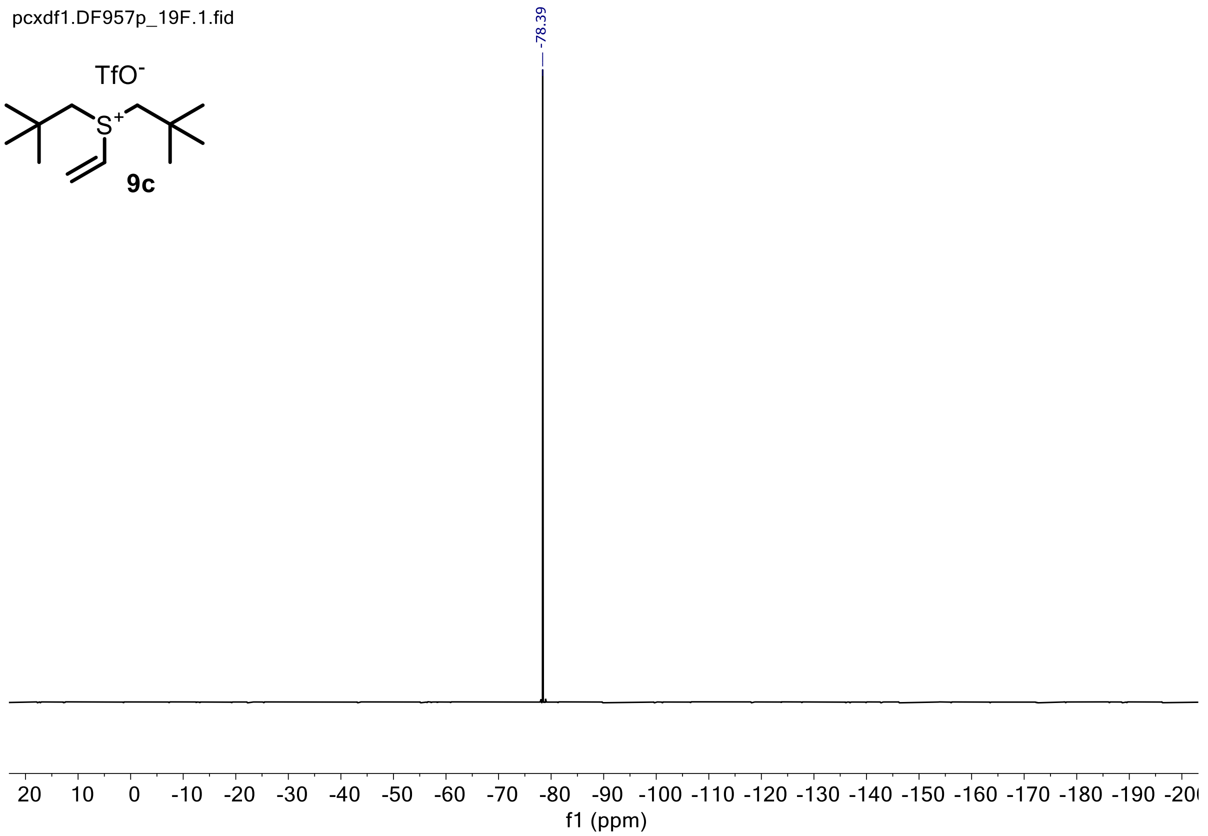

Isopentyldineopentylsulfonium triflate (S2)

^{1}H-NMR ($CDCl_3$, 500 MHz)

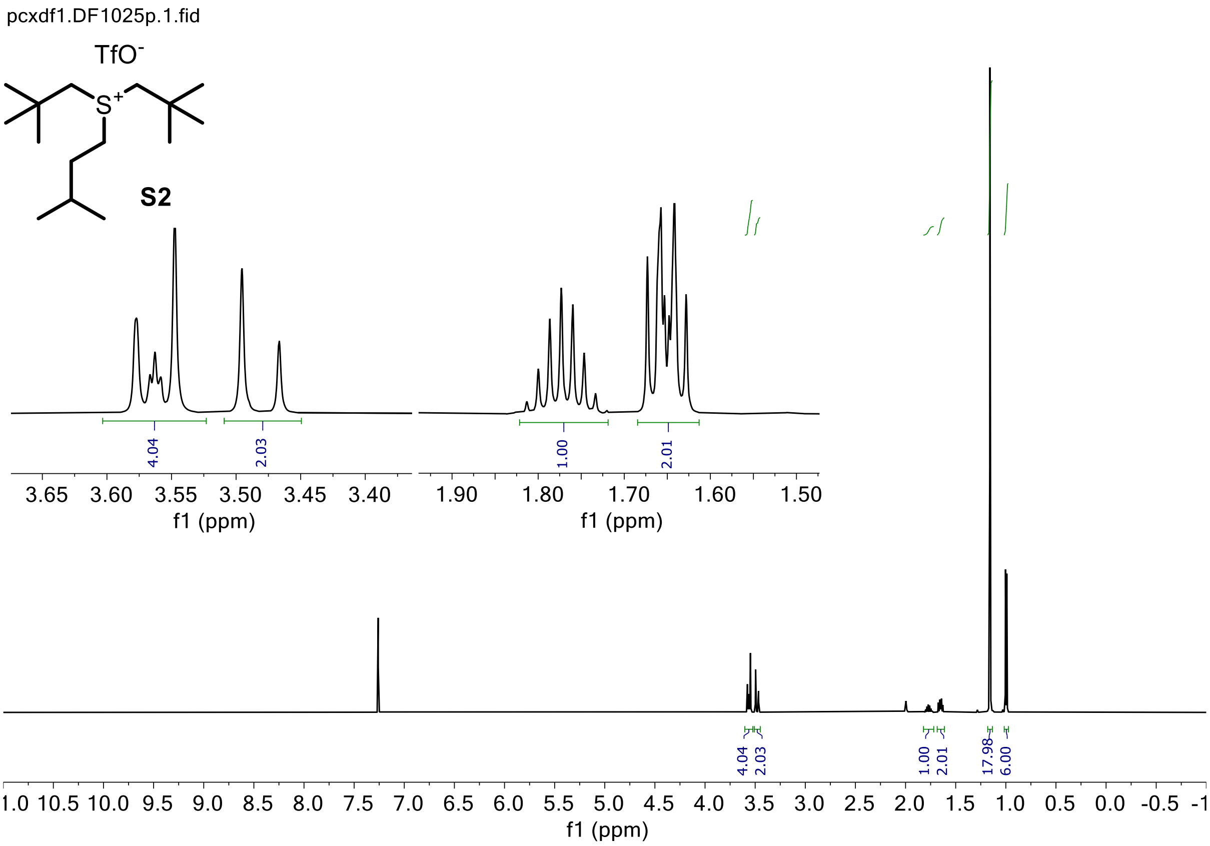

^{13}C-NMR ($CDCl_3$, 126 MHz)

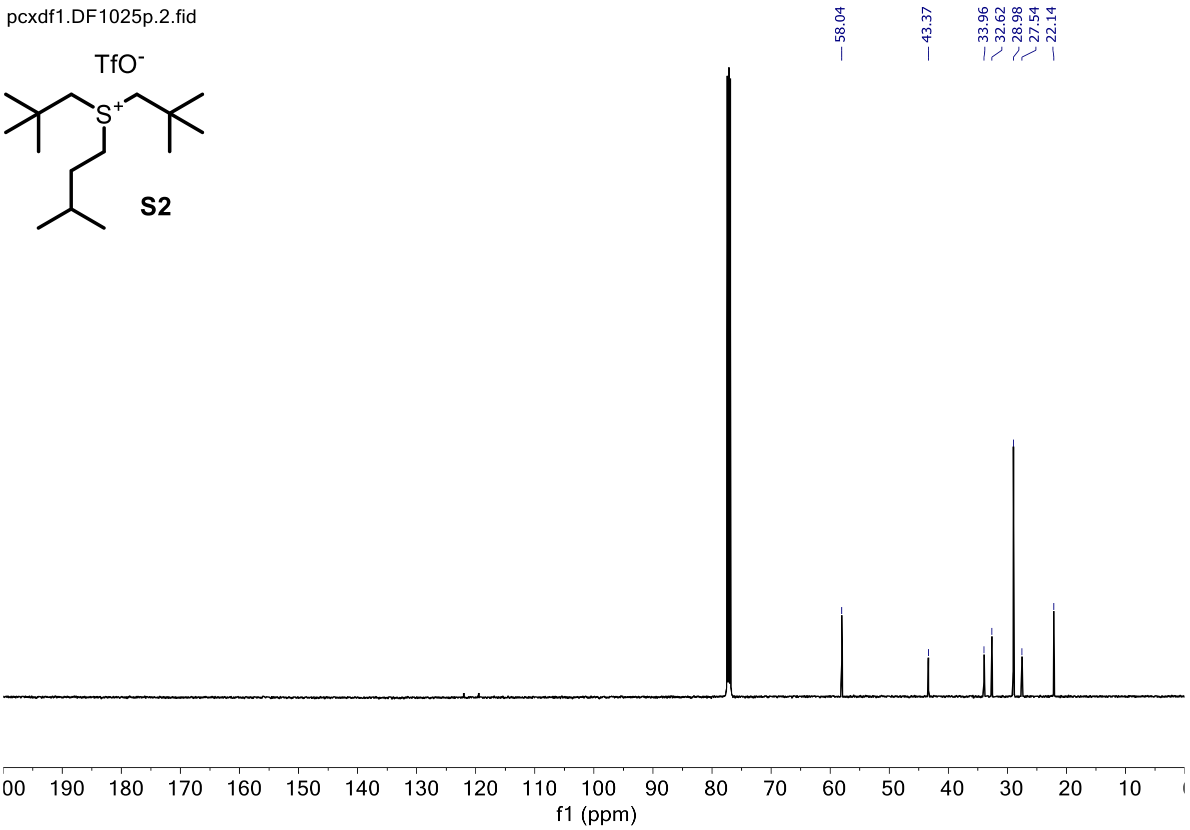

^{19}F-NMR (CDCl3, 376 MHz)

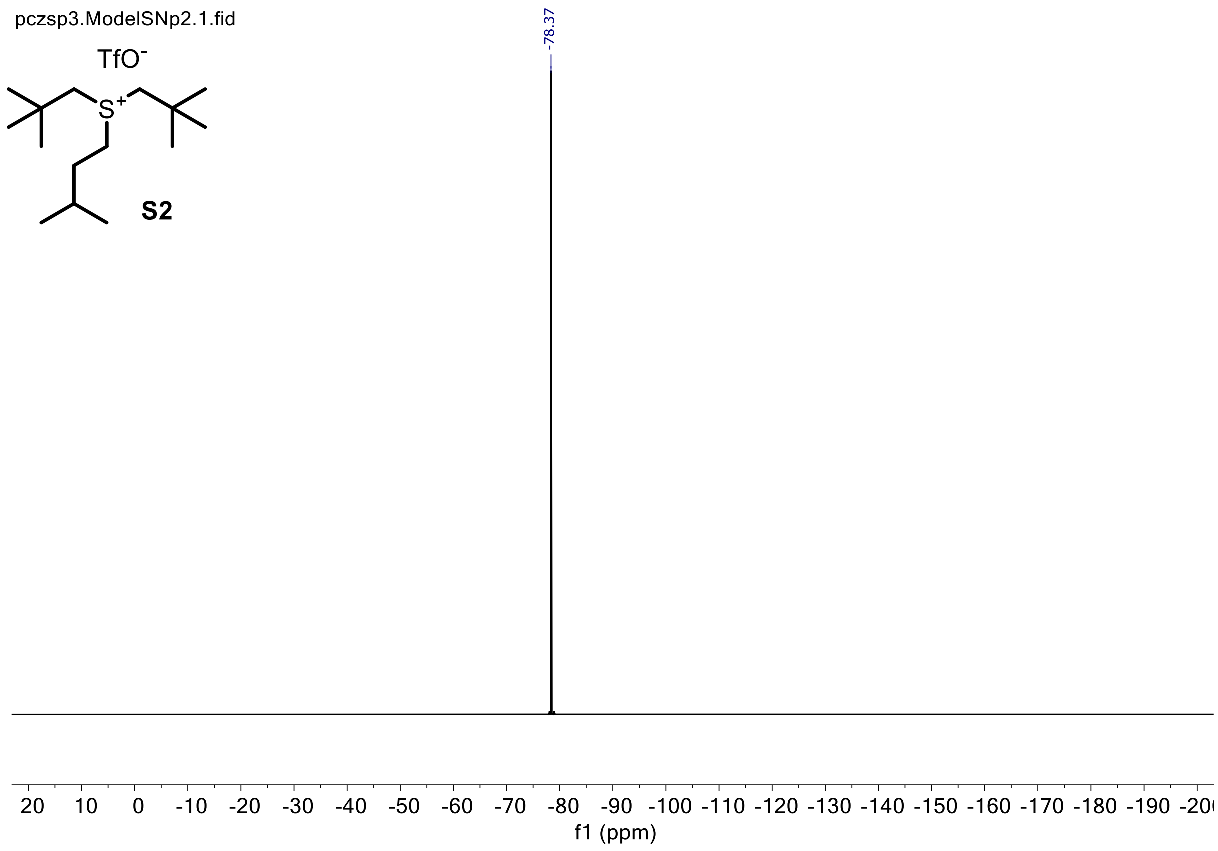

tert-butyl 4-(2-mercaptoethyl)piperidine-1-carboxylate (4d)

^{1}H-NMR ($CDCl_3$, 500 MHz)

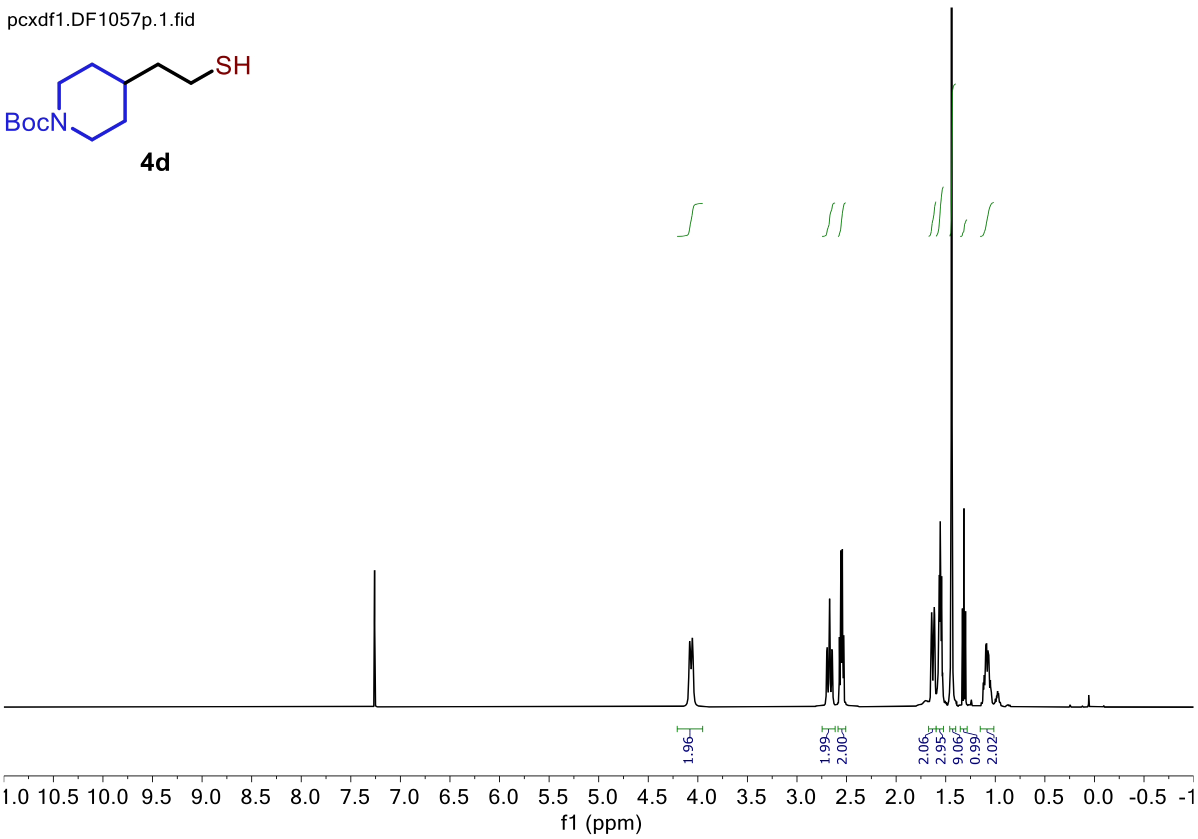

^{13}C-NMR ($CDCl_3$, 126 MHz)

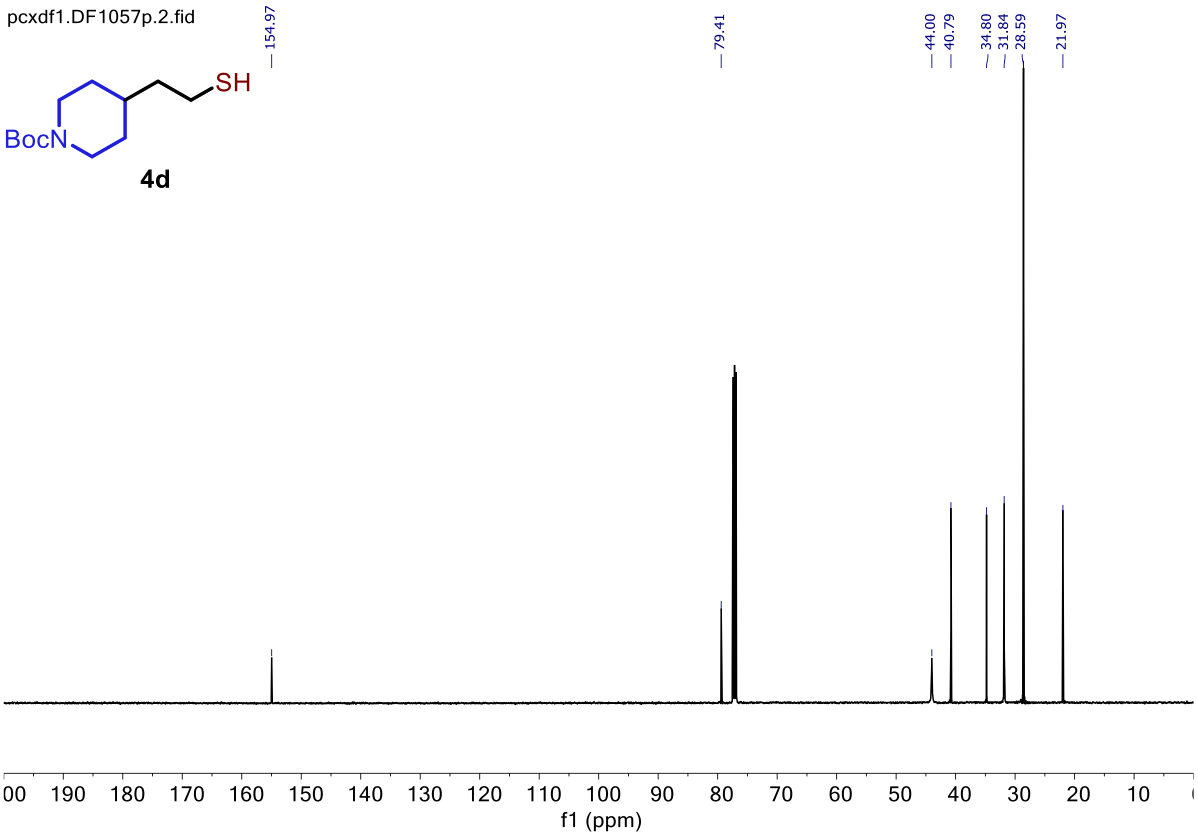

tert-butyl 4-(2-((2-hydroxyethyl)thio)ethyl)piperidine-1-carboxylate (4a)

^{1}H-NMR ($CDCl_3$, 500 MHz)

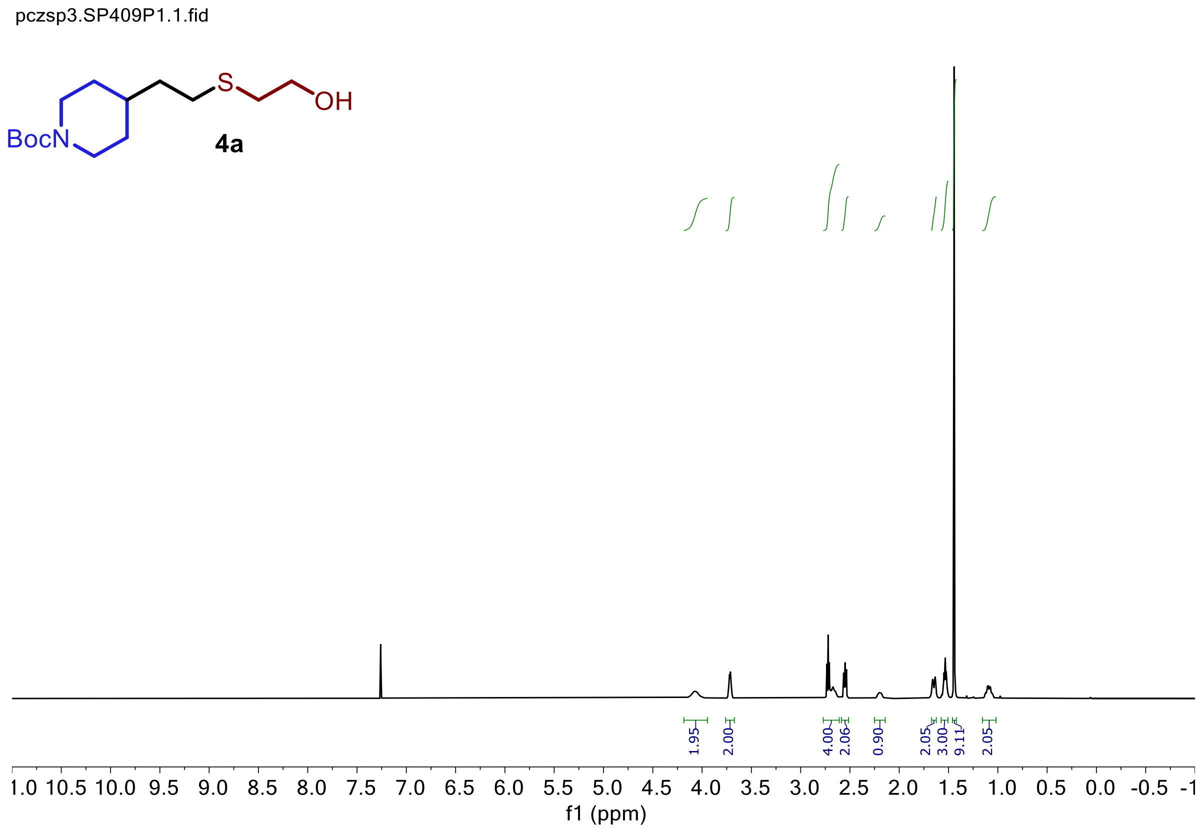

^{13}C-NMR ($CDCl_3$, 126 MHz)

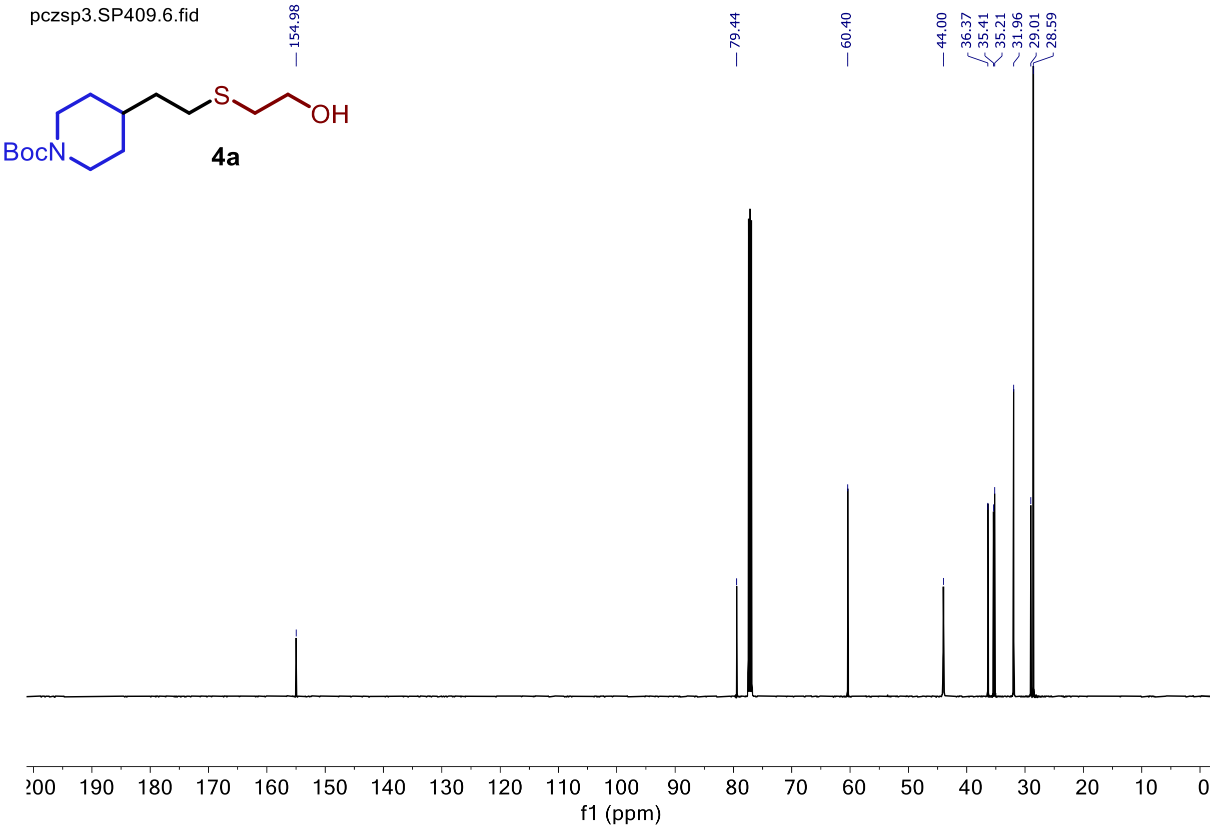

tert-butyl 4-(2-(cyclohexylthio)ethyl)piperidine-1-carboxylate (4e)

^{1}H-NMR (CDCl$_3$, 500 MHz)

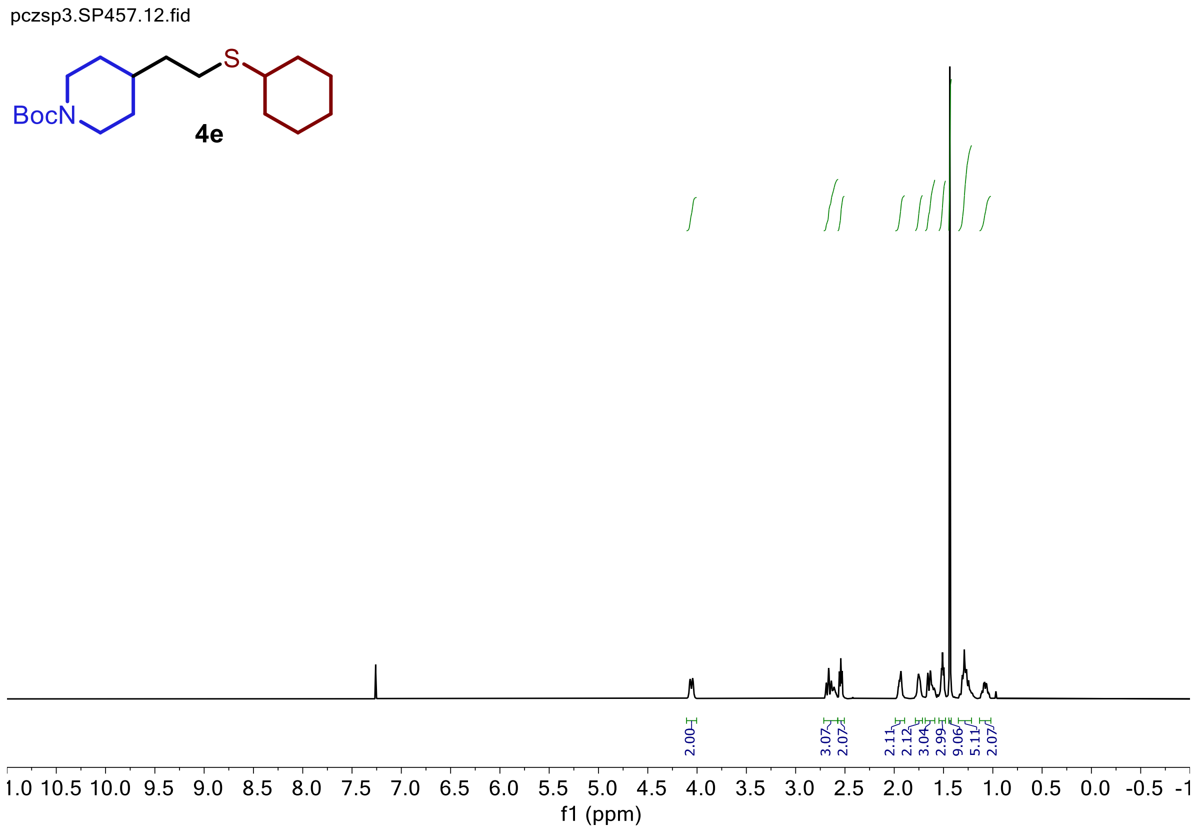

^{13}C-NMR (CDCl$_3$, 126 MHz)

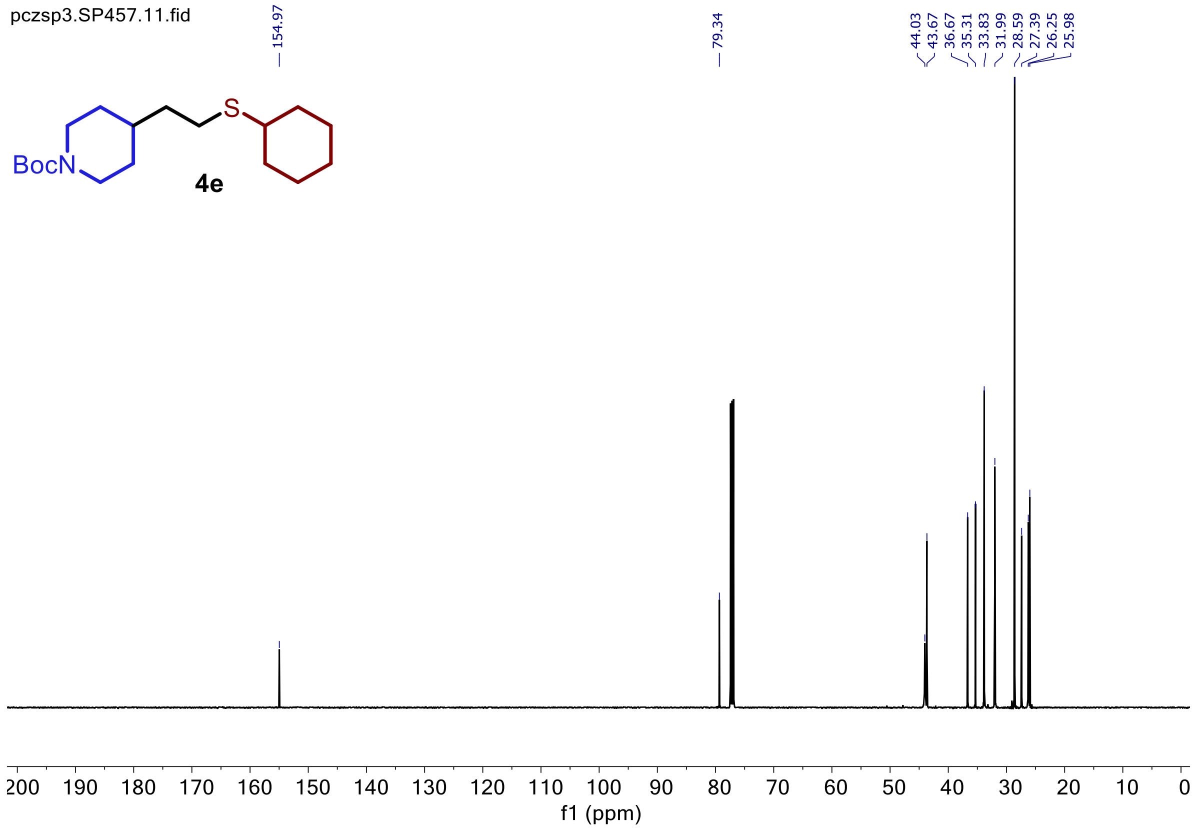

tert-butyl 4-(2-(phenylthio)ethyl)piperidine-1-carboxylate (4f)

^{1}H-NMR ($CDCl_3$, 500 MHz)

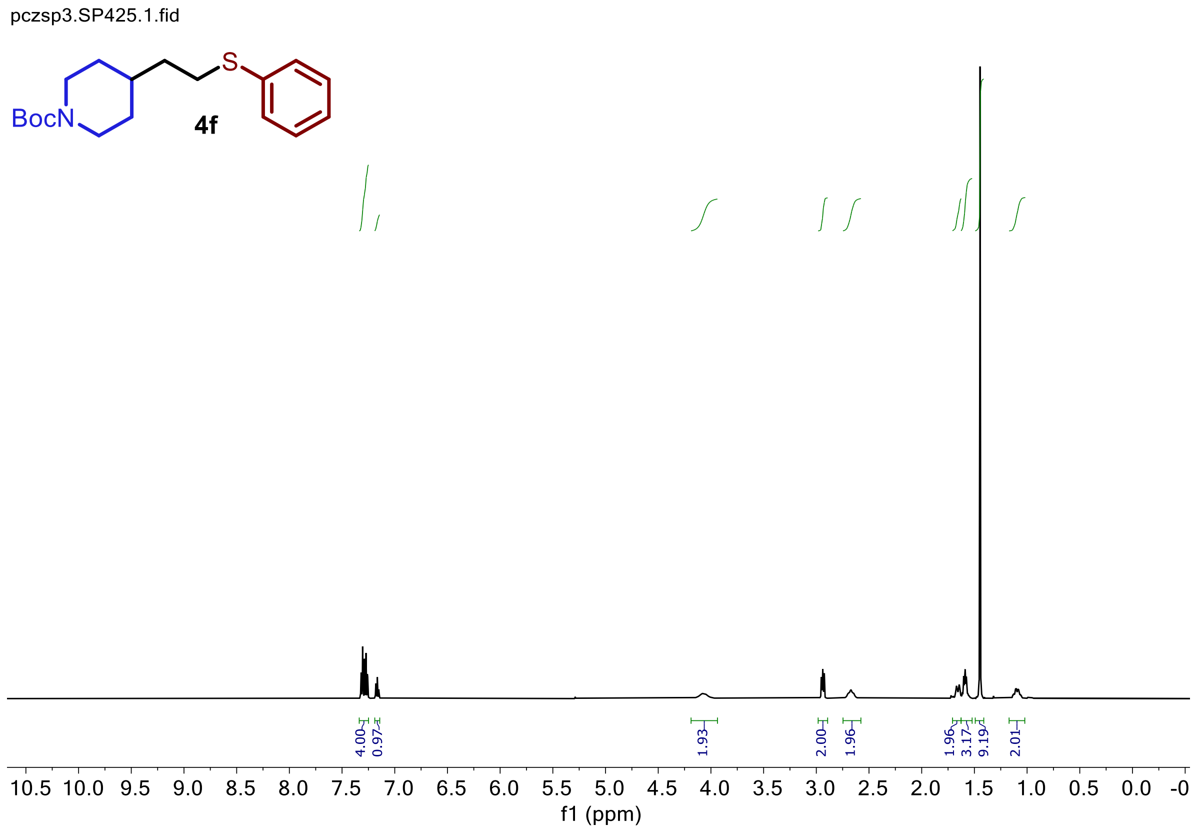

^{13}C-NMR ($CDCl_3$, 126 MHz)

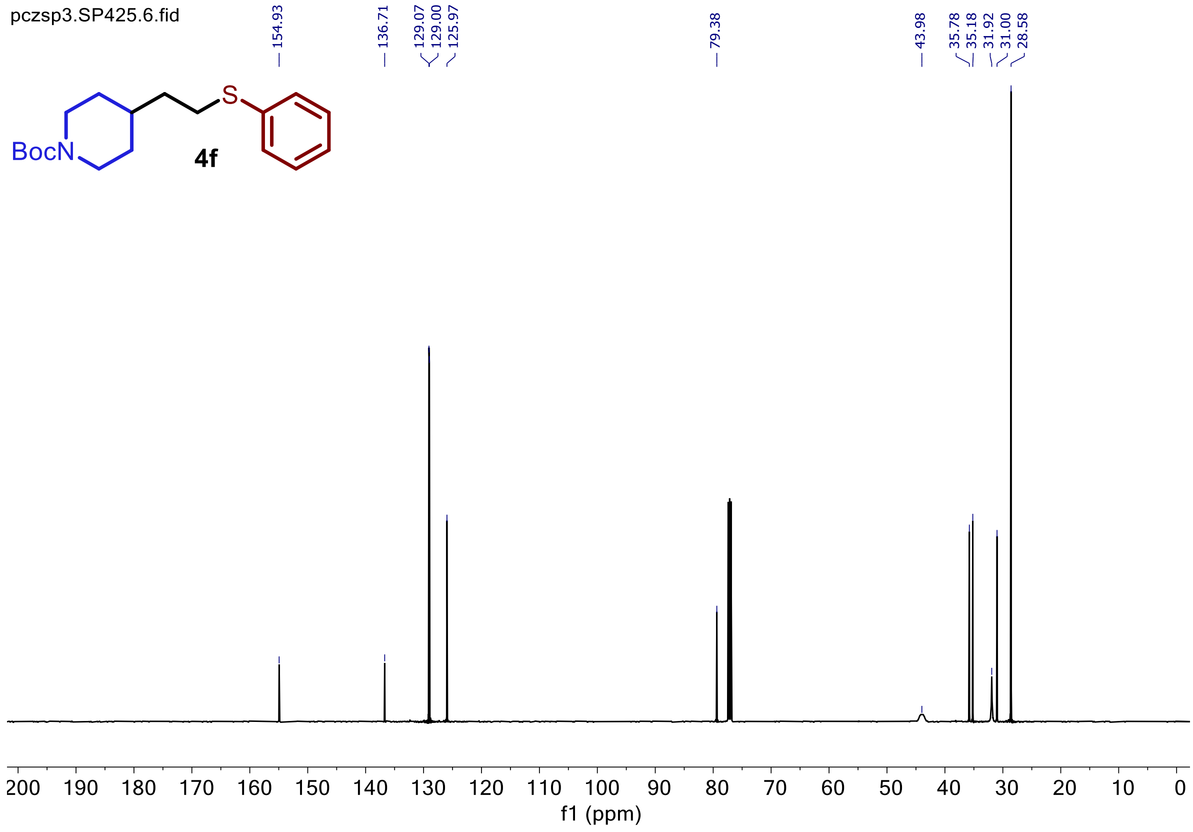

((*S*)-3-((2-(1-(tert-butoxycarbonyl)piperidin-4-yl)ethyl)thio)-2-methylpropanoyl)-*L*-proline (4g)

^{1}H-NMR ($CDCl_3$, 500 MHz)

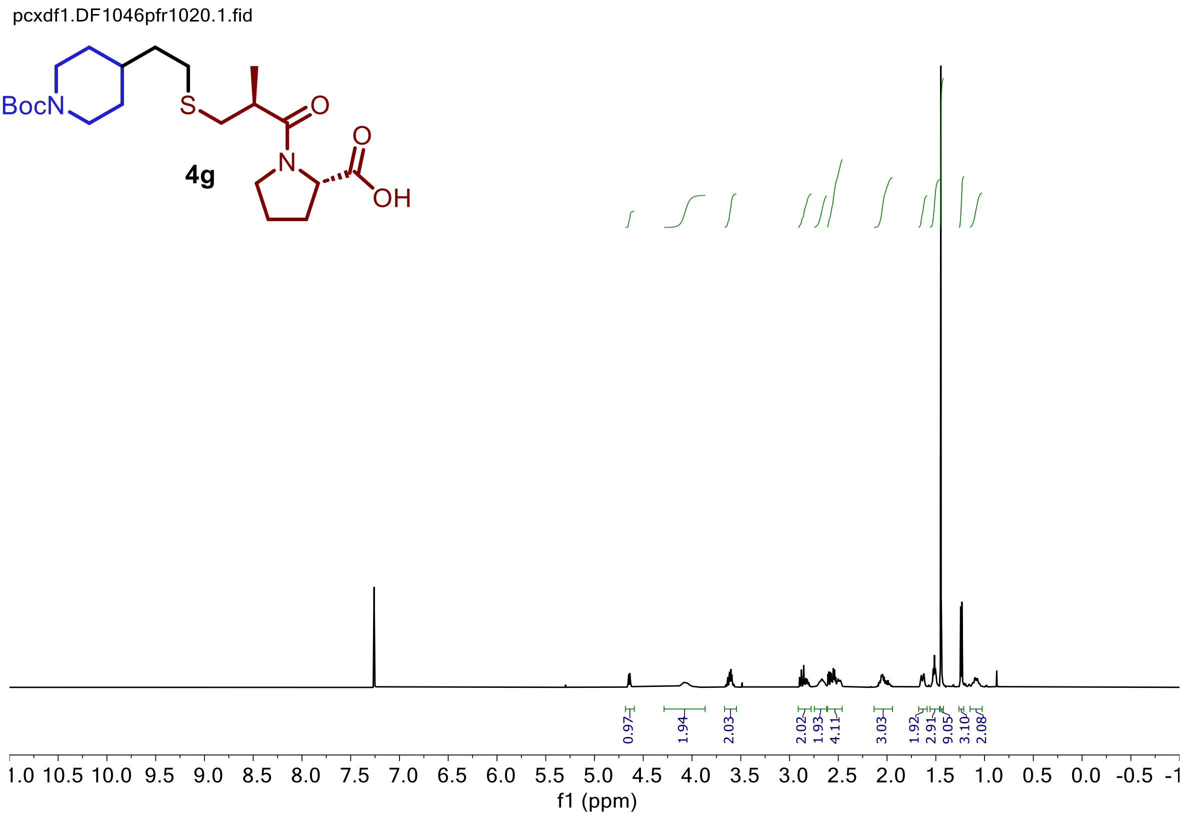

^{13}C-NMR ($CDCl_3$, 126 MHz)

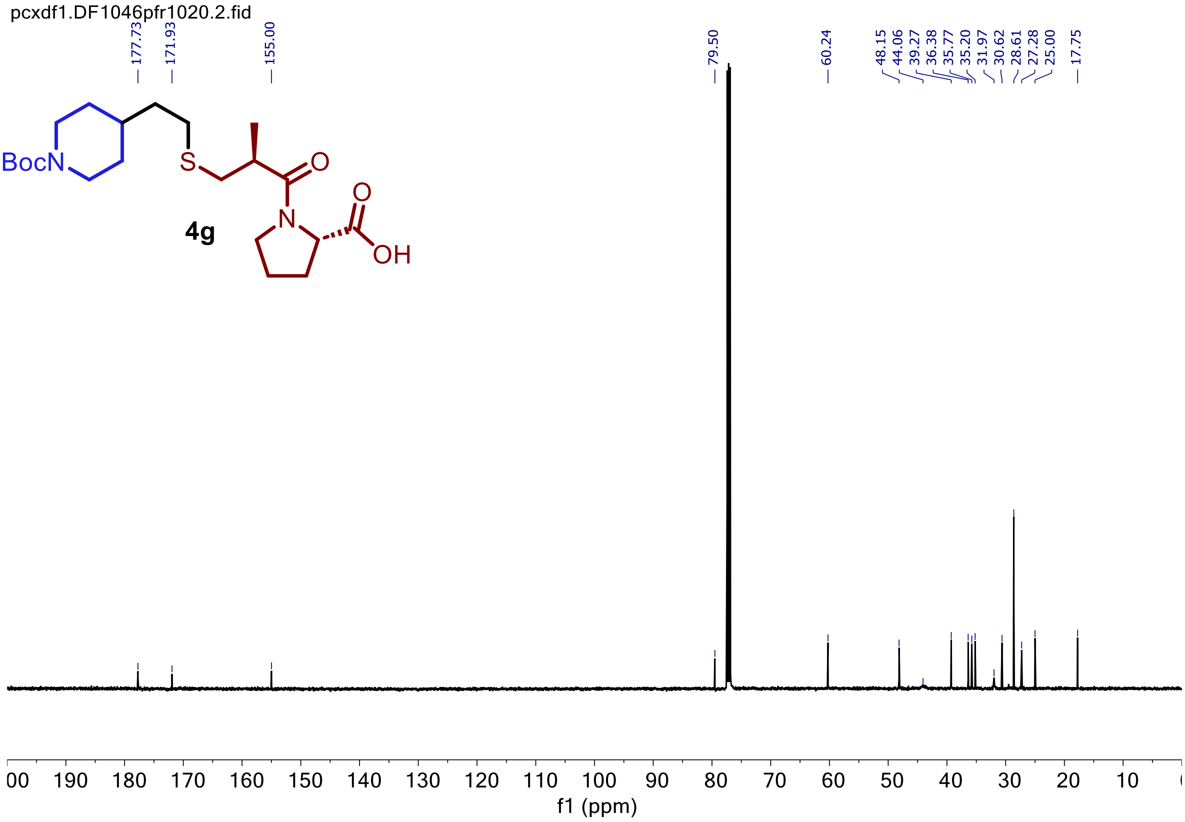

tert-butyl 4-(2-hydroxyethyl)piperidine-1-carboxylate (4h)

^{1}H-NMR ($CDCl_3$, 500 MHz)

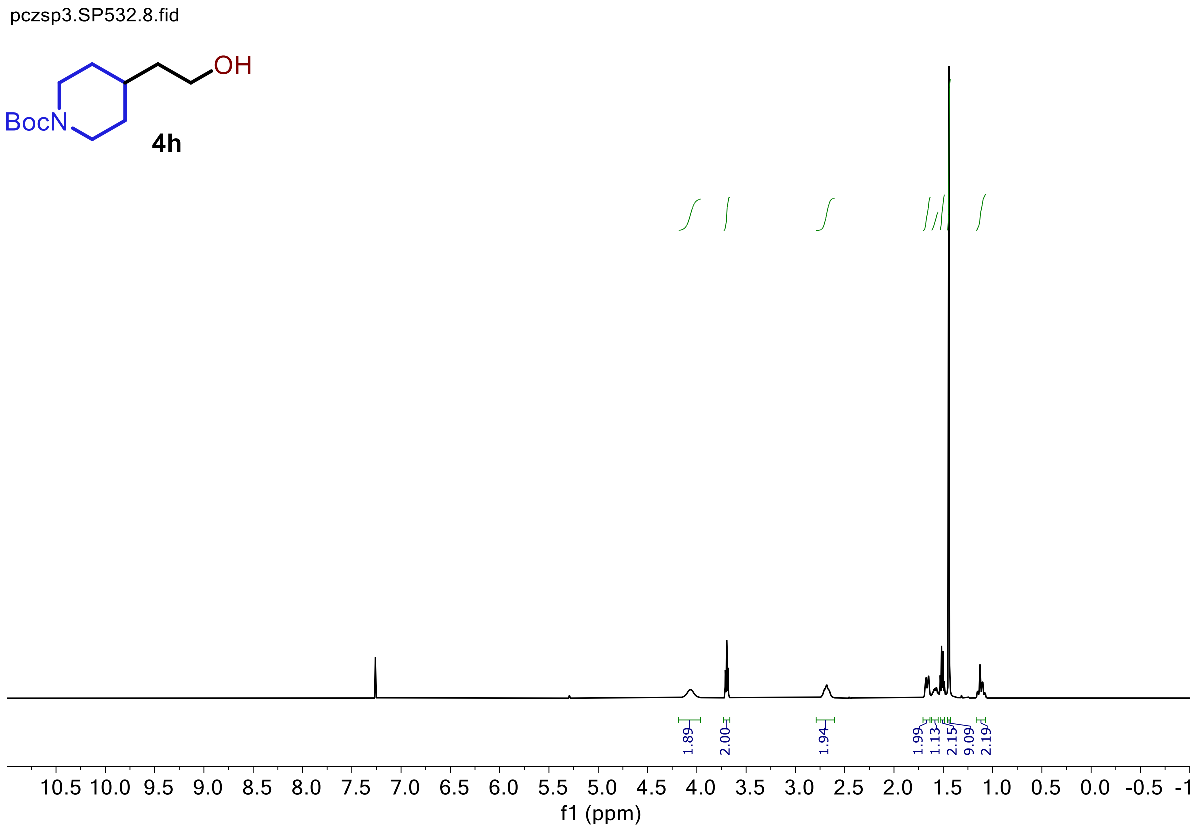

^{13}C-NMR ($CDCl_3$, 126 MHz)

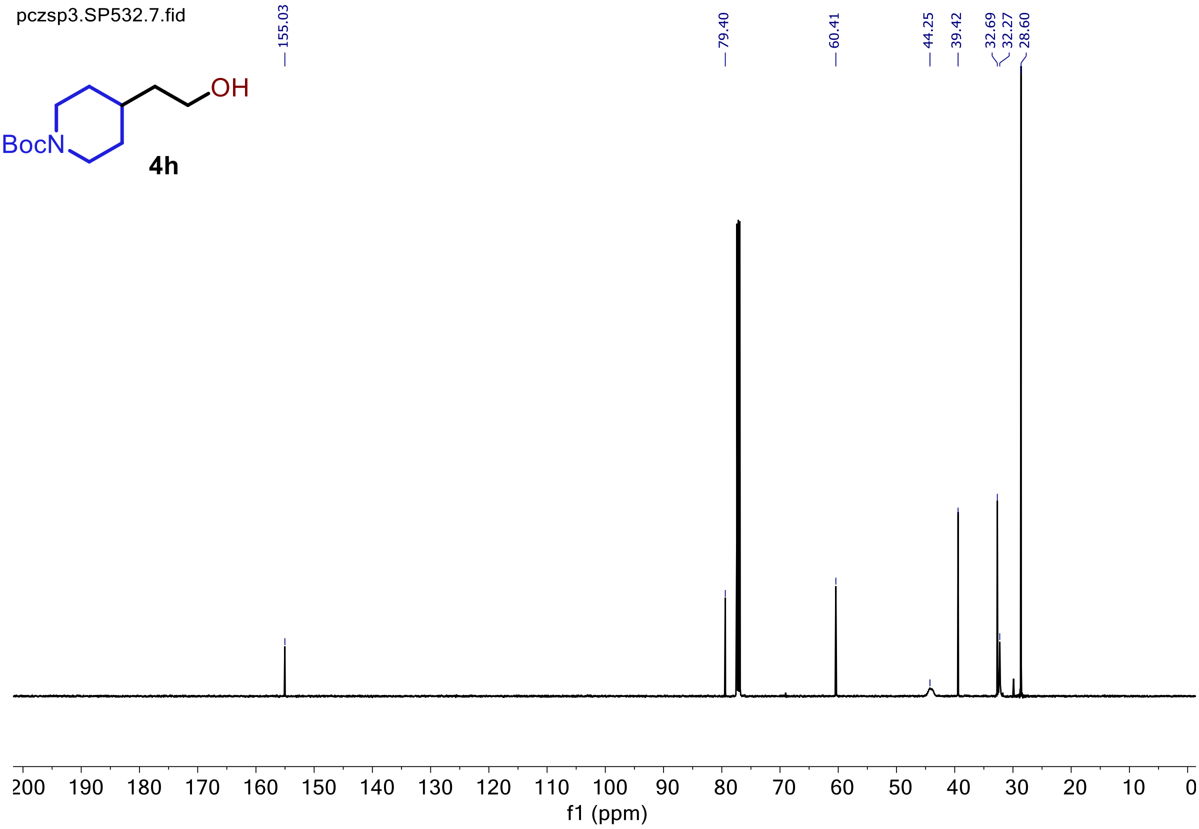

***tert*-butyl 4-(2-(benzyloxy)ethyl)piperidine-1-carboxylate (4i)**

^{1}H-NMR ($CDCl_3$, 500 MHz)

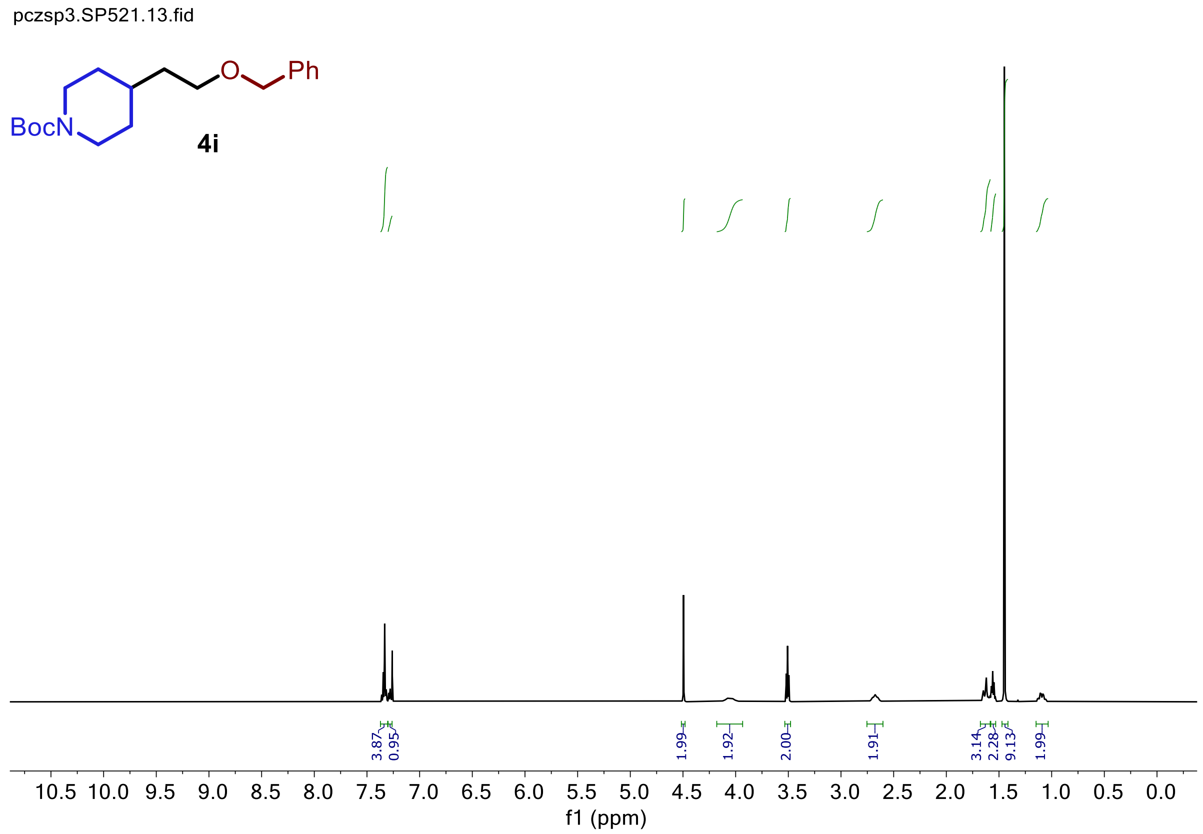

^{13}C-NMR ($CDCl_3$, 126 MHz)

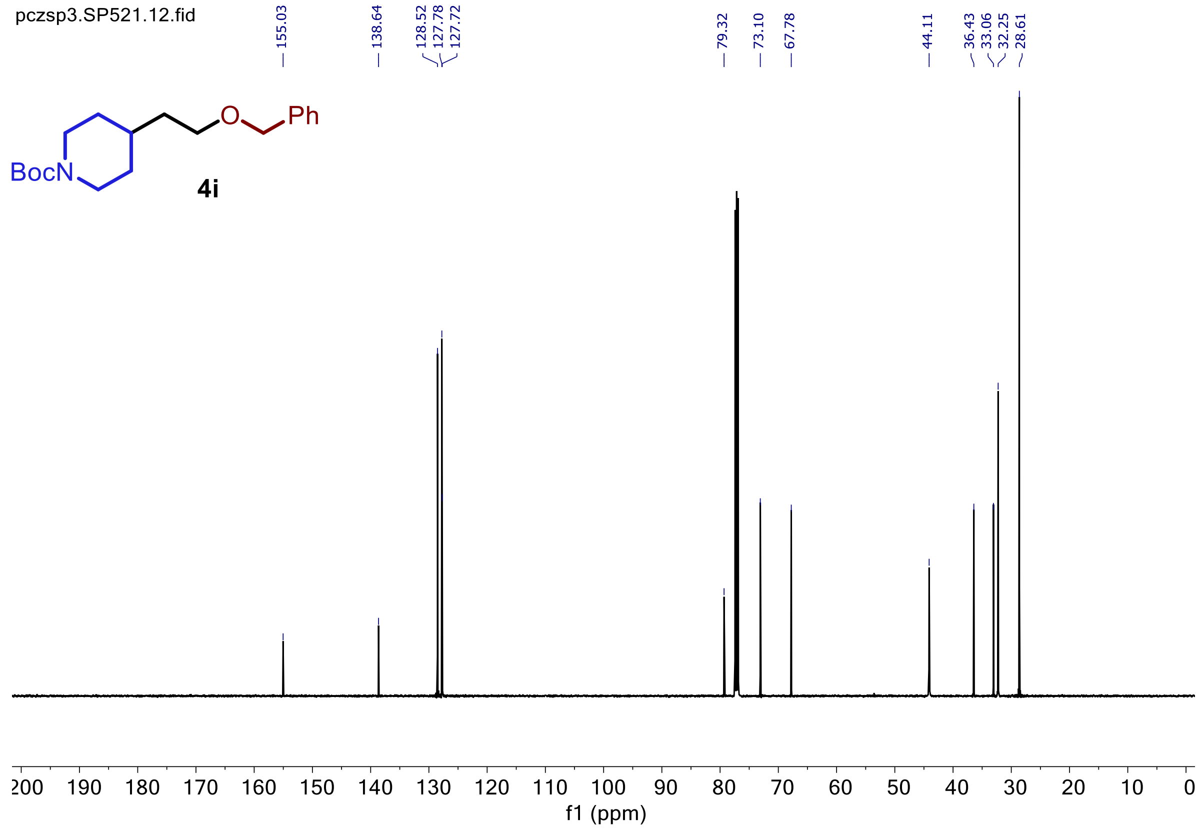

tert-butyl 4-(2-cyclobutoxyethyl)piperidine-1-carboxylate (4j)

^{1}H-NMR ($CDCl_3$, 500 MHz)

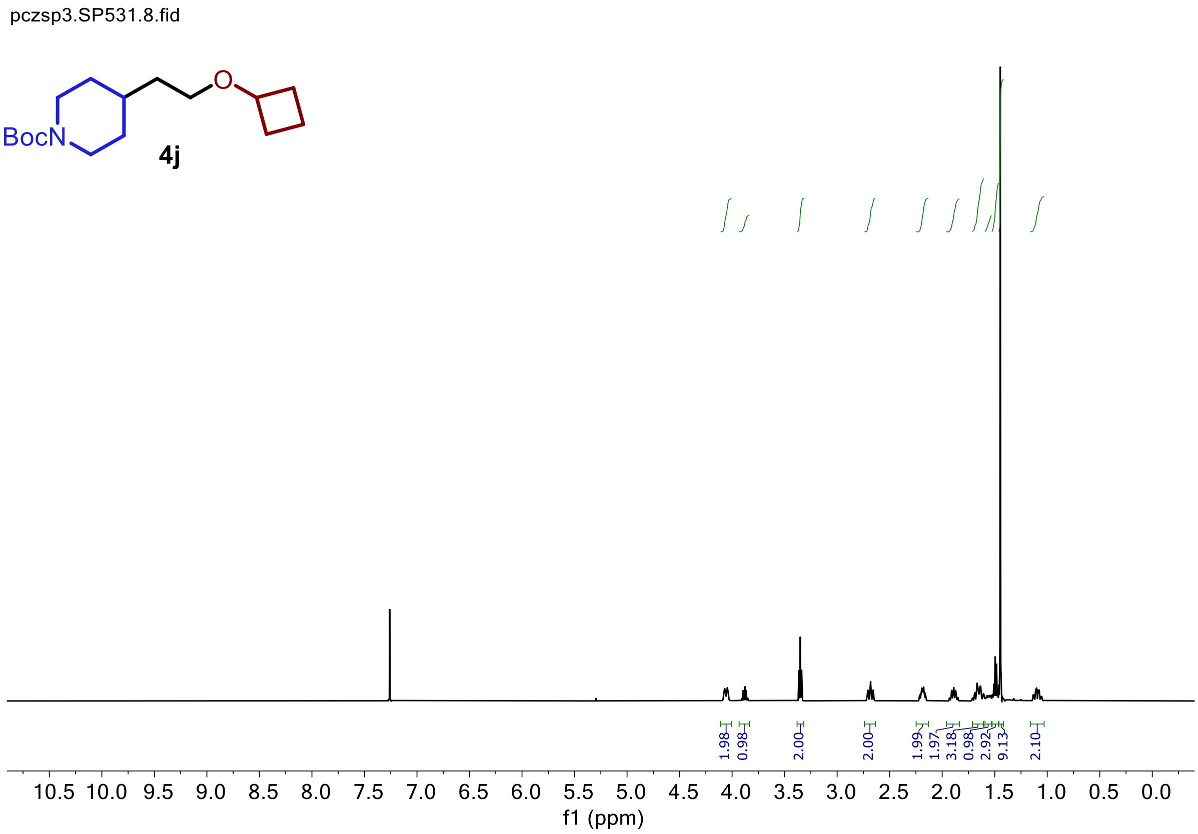

^{13}C-NMR ($CDCl_3$, 126 MHz)

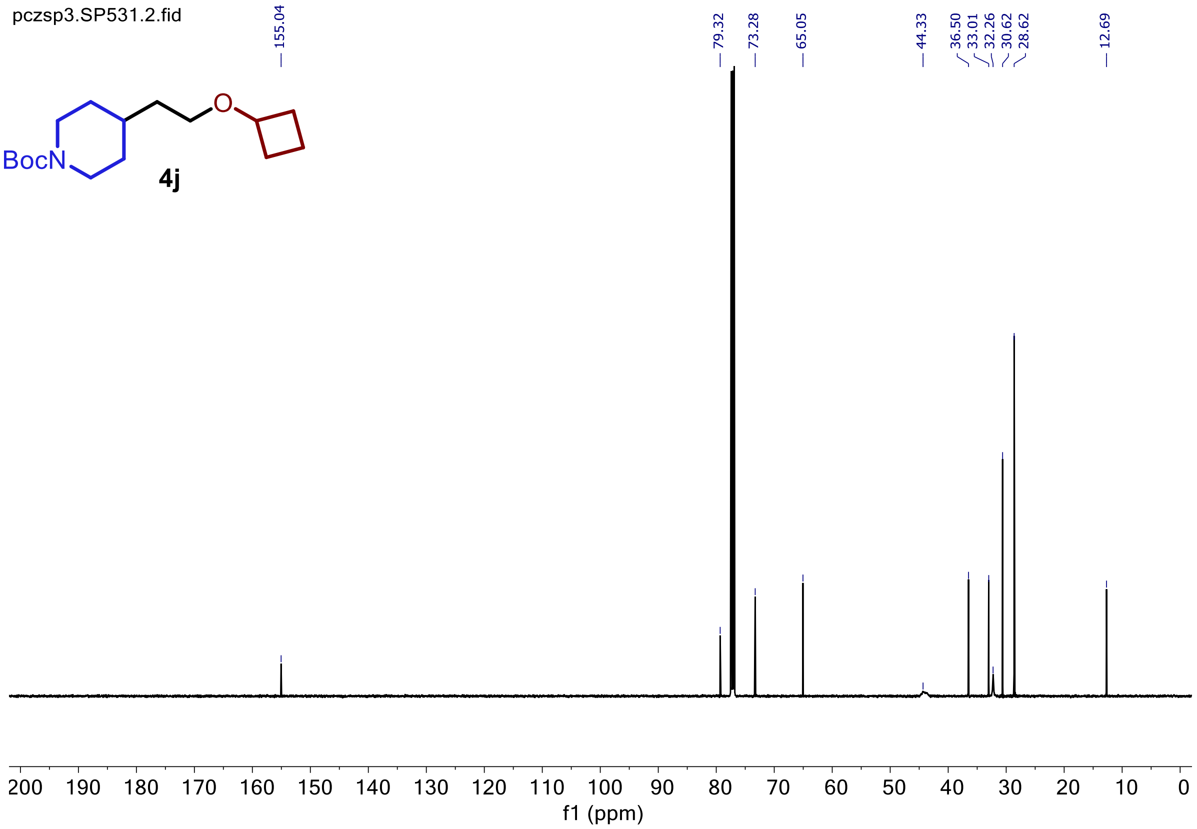

tert-butyl 4-(2-phenoxyethyl)piperidine-1-carboxylate (4b)

^{1}H-NMR ($CDCl_3$, 500 MHz)

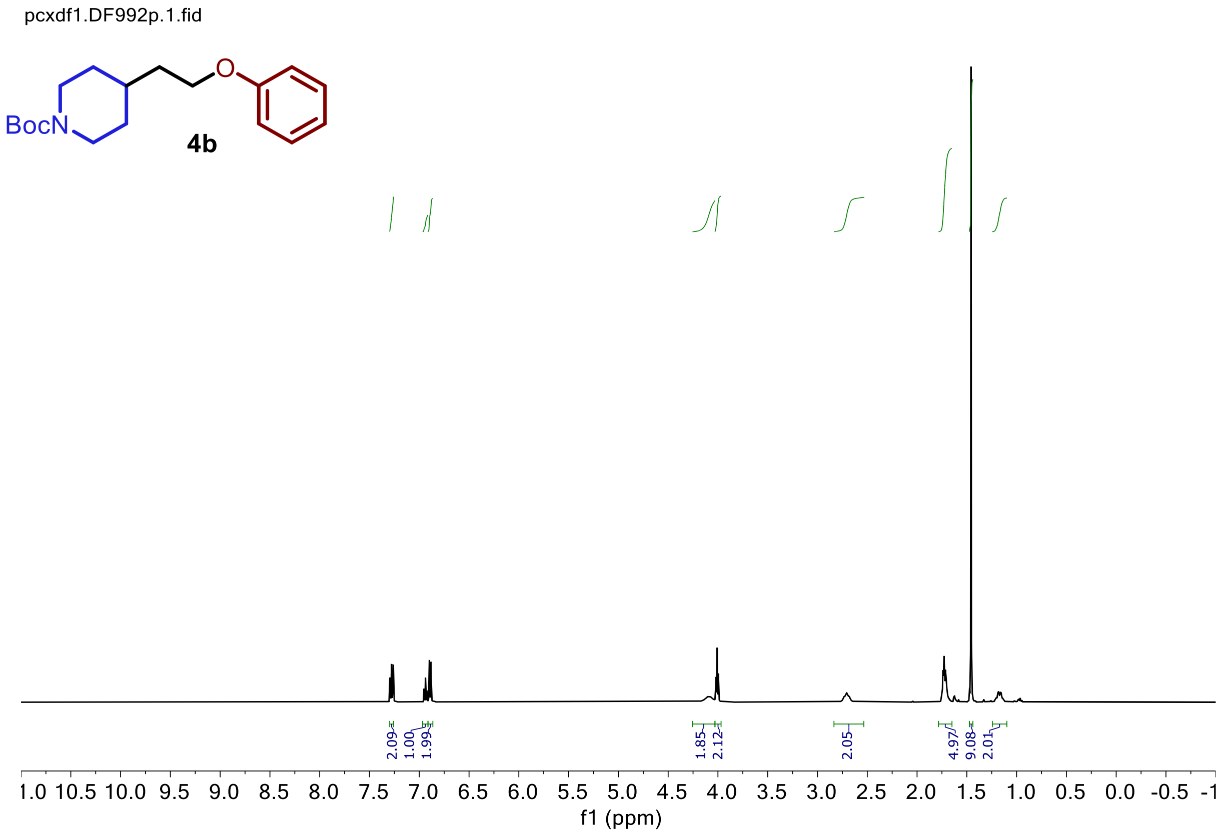

^{13}C-NMR ($CDCl_3$, 126 MHz)

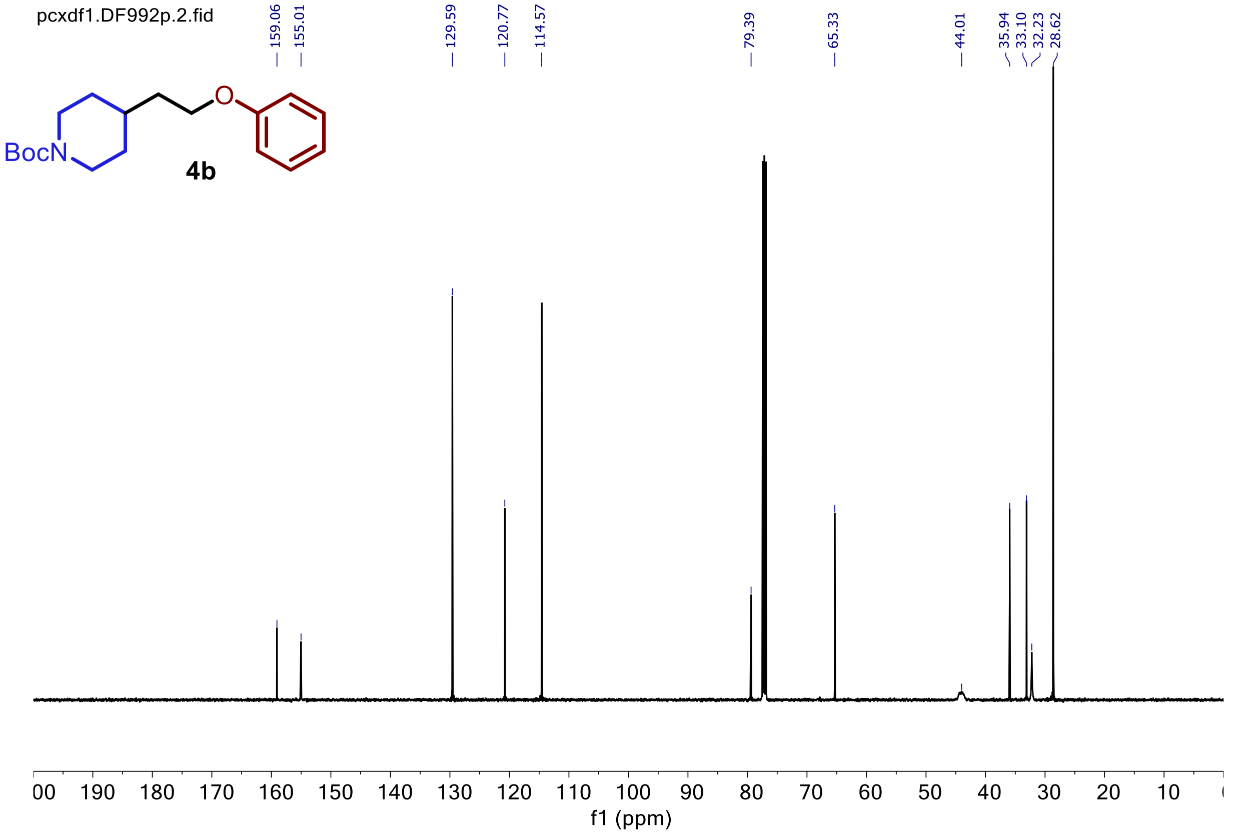

***tert*-butyl 4-(2-(((4*R*,4a*R*,7*S*,7a*R*,12b*S*)-7-hydroxy-3-methyl-2,3,4,4a,7,7a-hexahydro-1*H*-4,12-methanobenzofuro[3,2-*e*]isoquinolin-9-yl)oxy)ethyl)piperidine-1-carboxylate (4k)**

^{1}H-NMR ($CDCl_3$, 500 MHz)

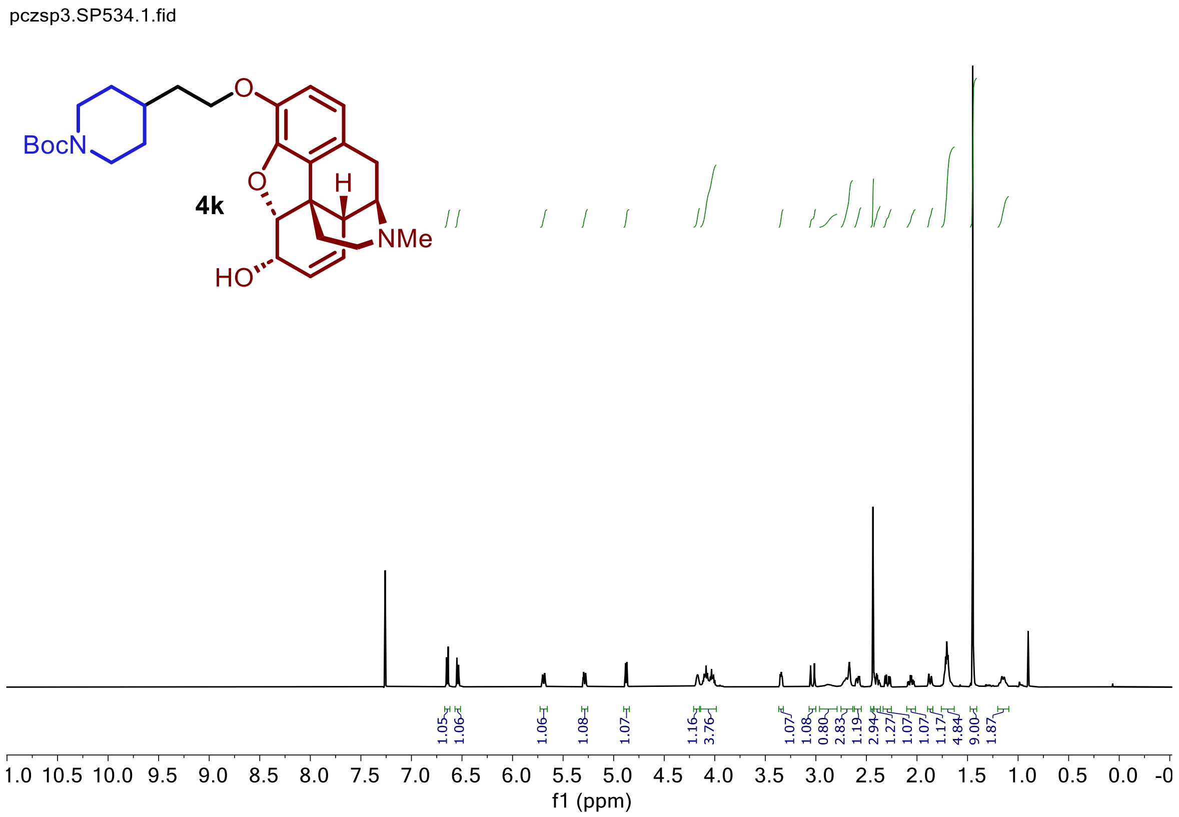

^{13}C-NMR ($CDCl_3$, 126 MHz)

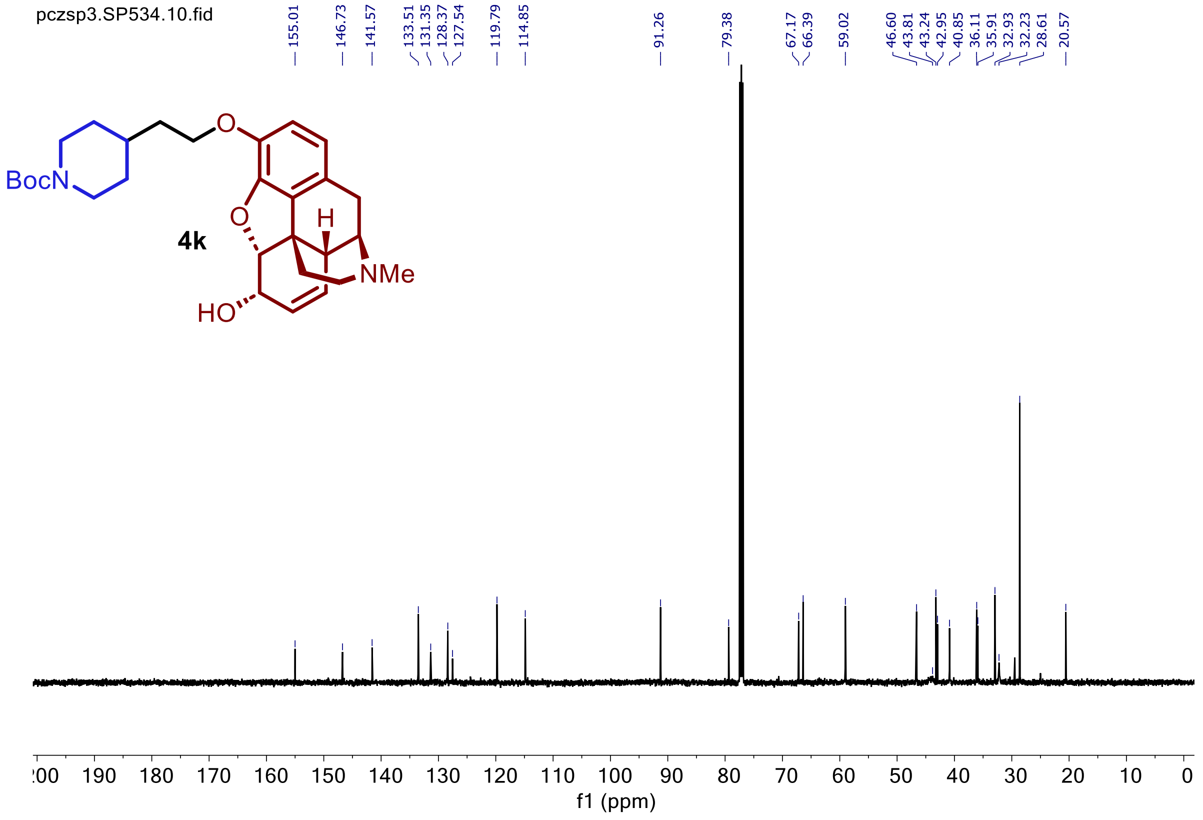

***tert*-butyl 4-(2-((5-((3a*S*,4*S*,6a*R*)-2-oxohexahydro-1*H*-thieno[3,4-*d*]imidazol-4-yl)pentanoyl)oxy)ethyl)piperidine-1-carboxylate (4l)**

^{1}H-NMR ($CDCl_3$, 500 MHz)

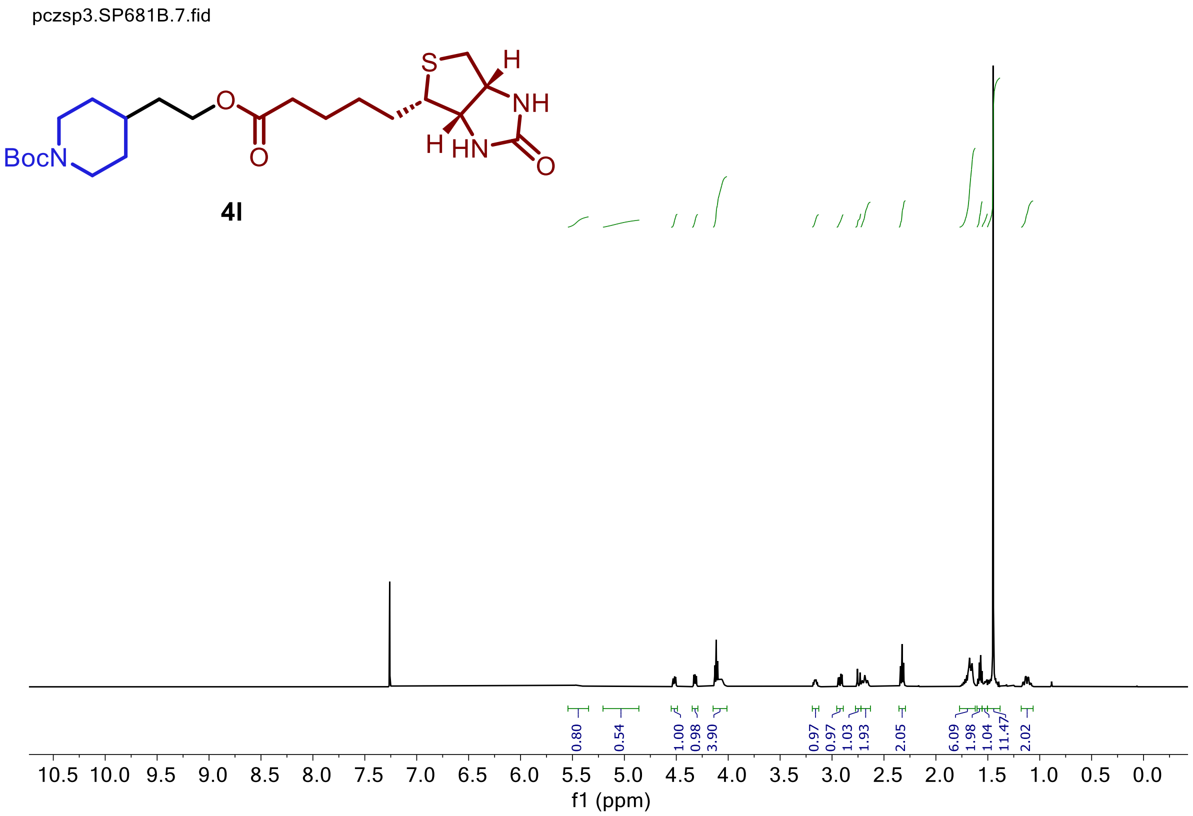

^{1}H-NMR (DMSO-d_6, 500 MHz)

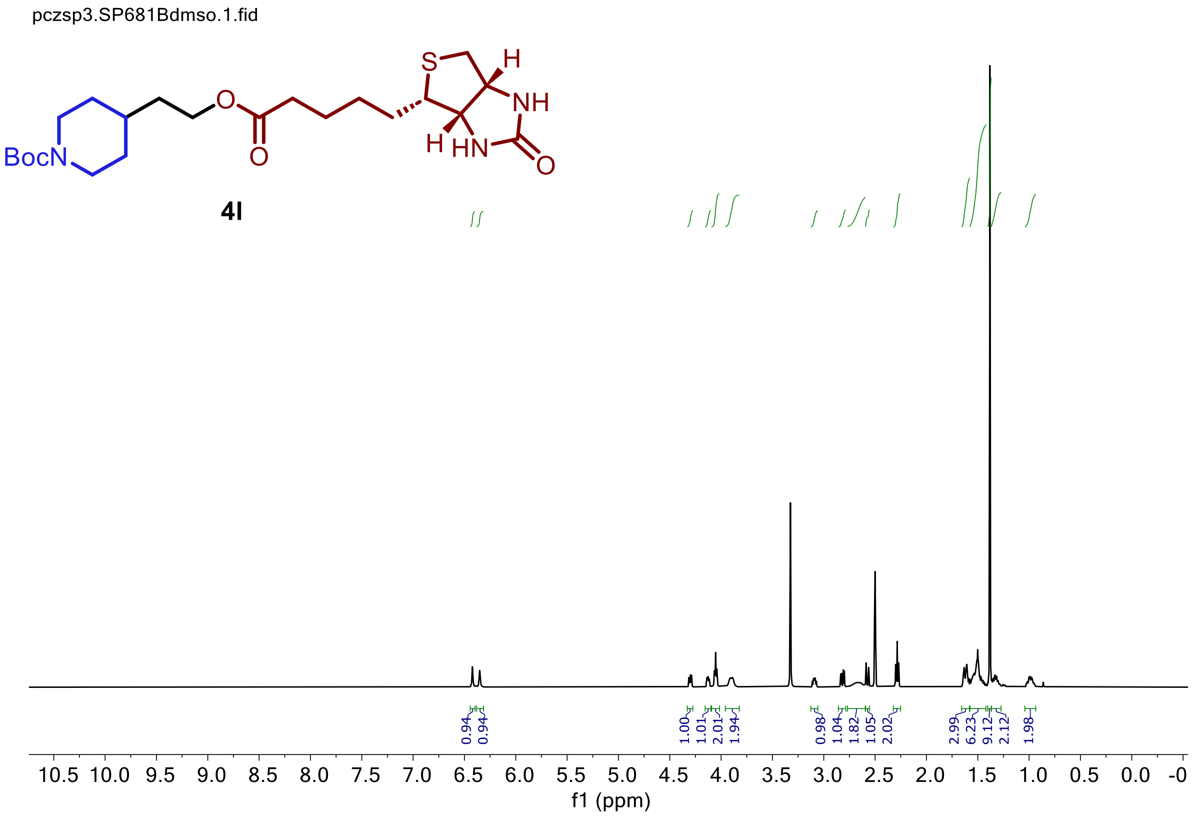

^{13}C-NMR ($CDCl_3$, 126 MHz)

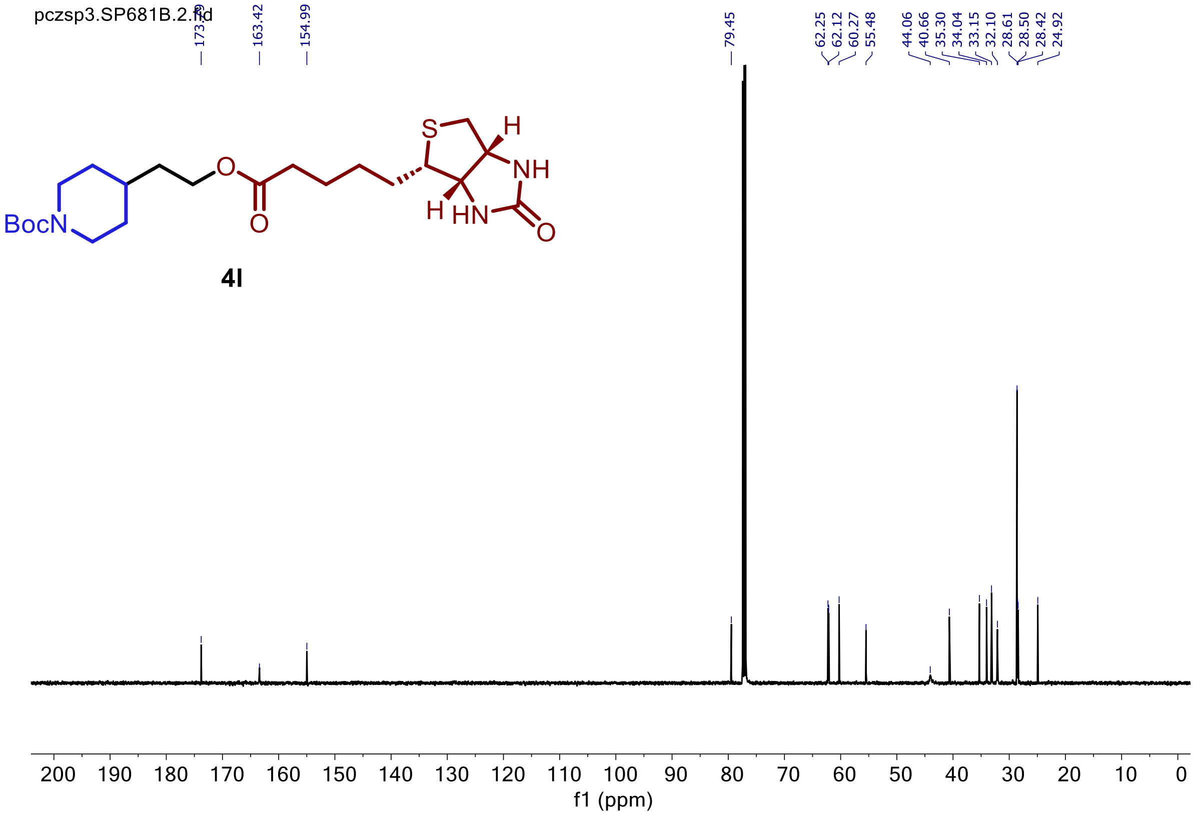

***tert*-butyl 4-(2-aminoethyl)piperidine-1-carboxylate (4m)**

^{1}H-NMR ($CDCl_3$, 500 MHz)

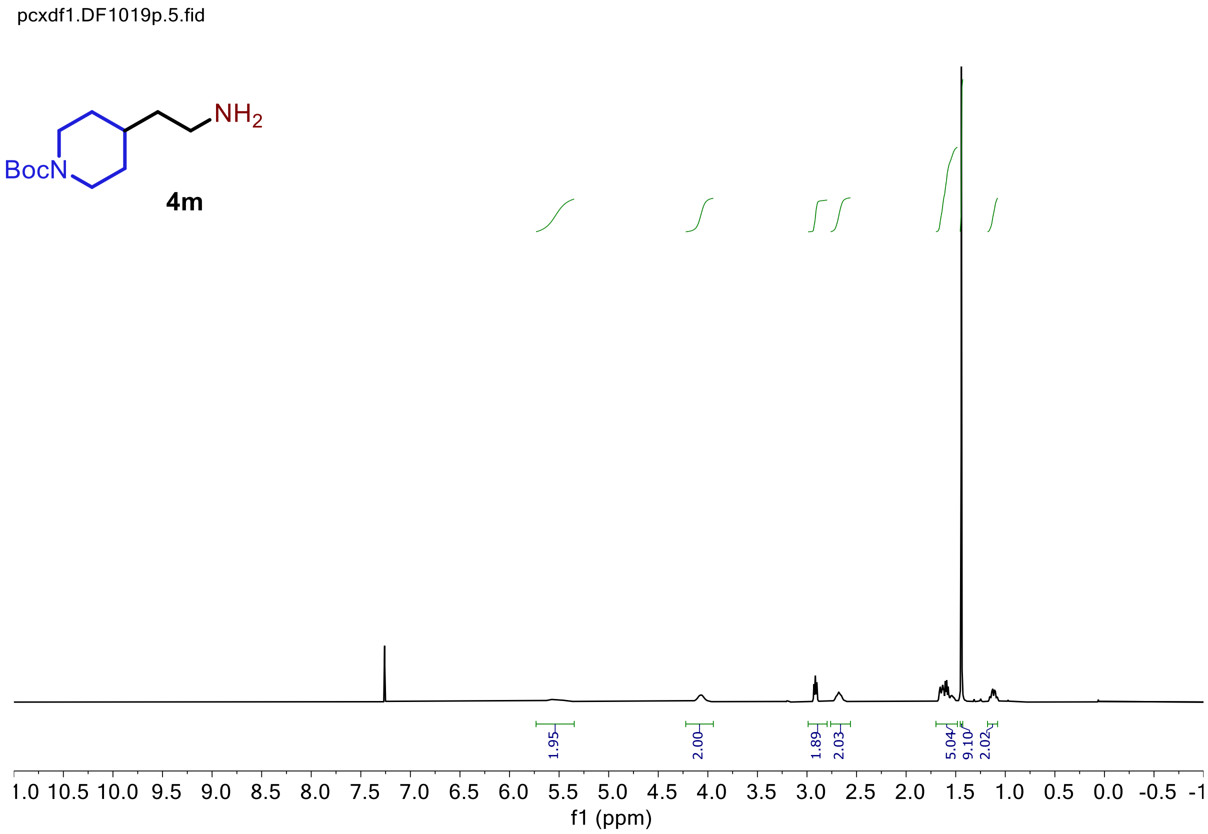

^{13}C-NMR ($CDCl_3$, 126 MHz)

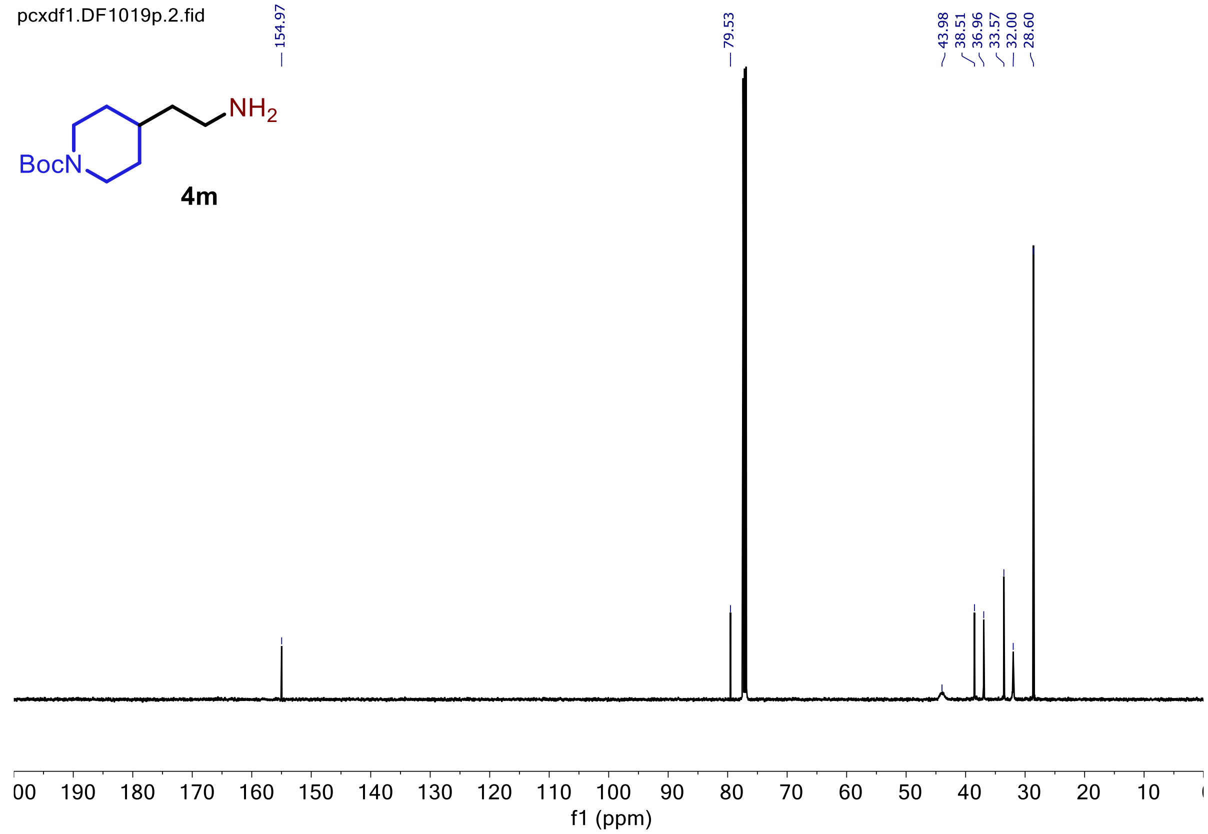

tert-butyl 4-(2-(benzylamino)ethyl)piperidine-1-carboxylate (4c)

^{1}H-NMR (CDCl$_3$, 500 MHz)

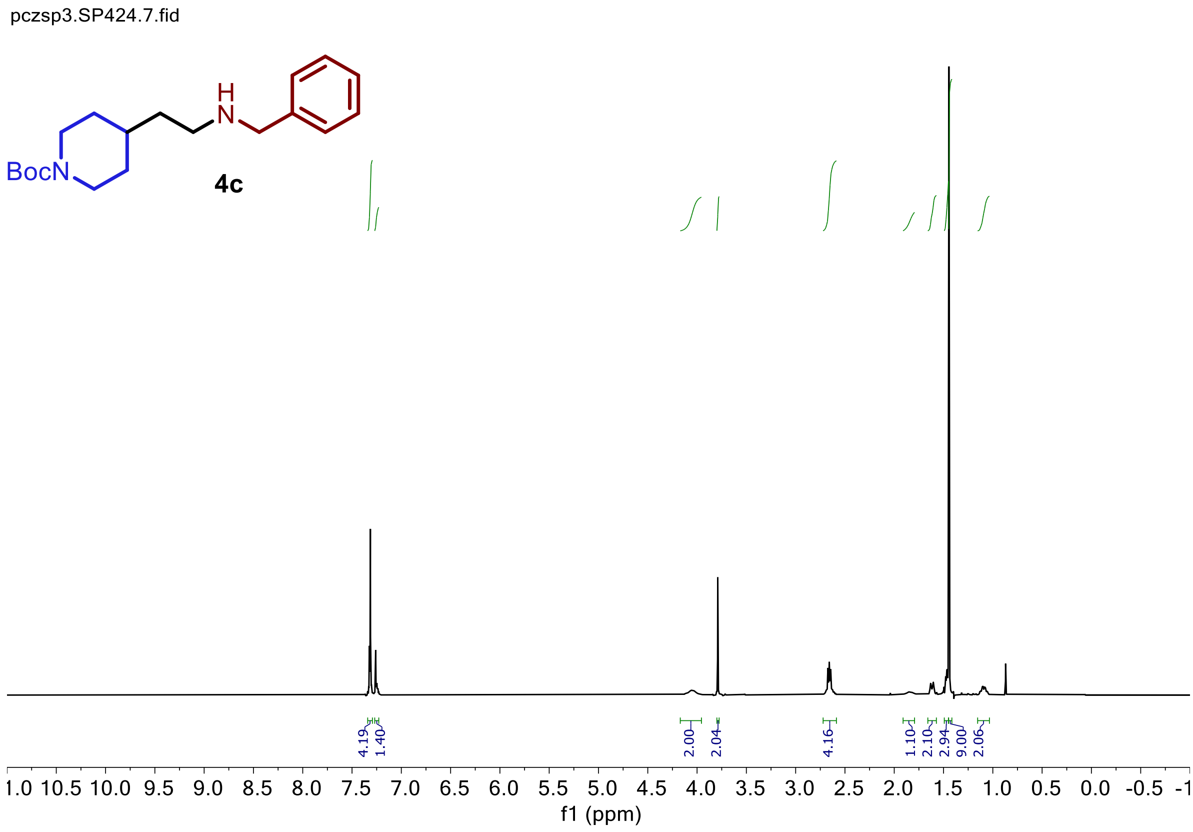

^{13}C-NMR (CDCl$_3$, 126 MHz)

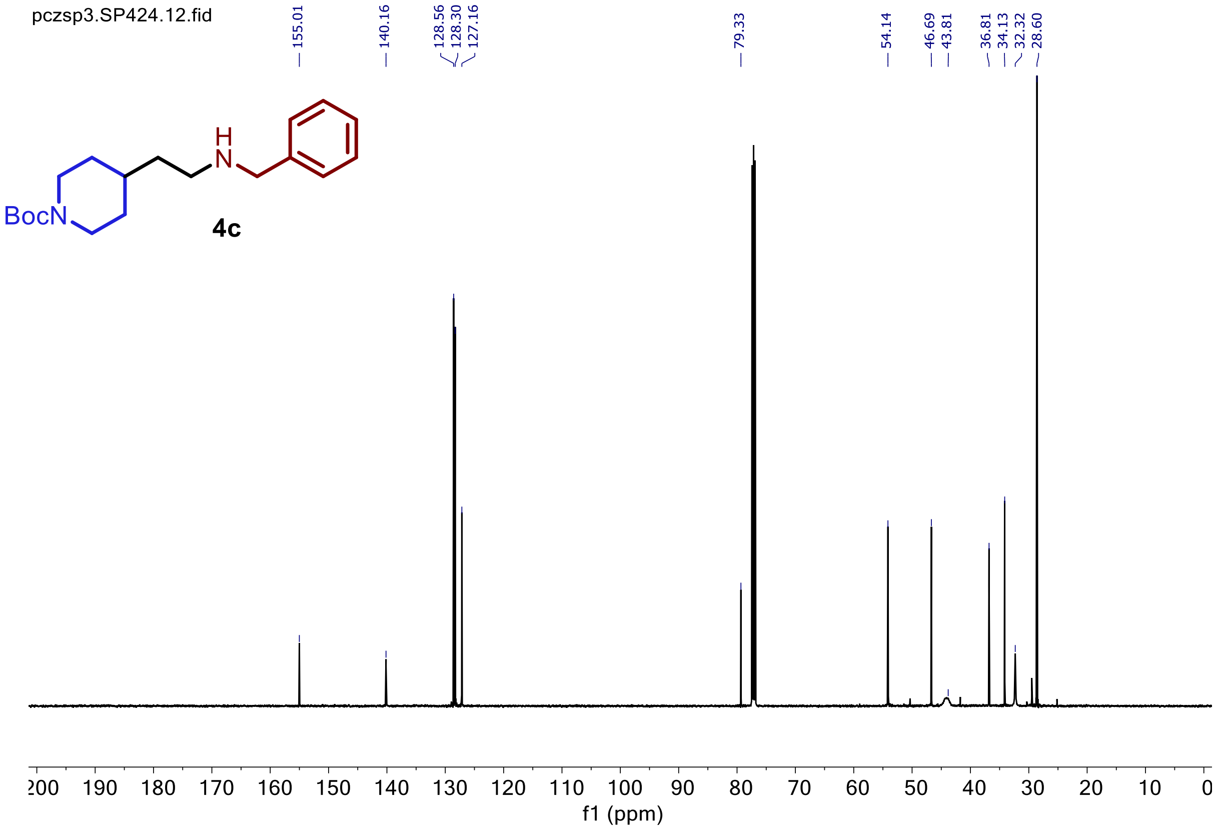

***tert*-butyl 4-(2-(pyrrolidin-1-yl)ethyl)piperidine-1-carboxylate (4n)**

^{1}H-NMR ($CDCl_3$, 500 MHz)

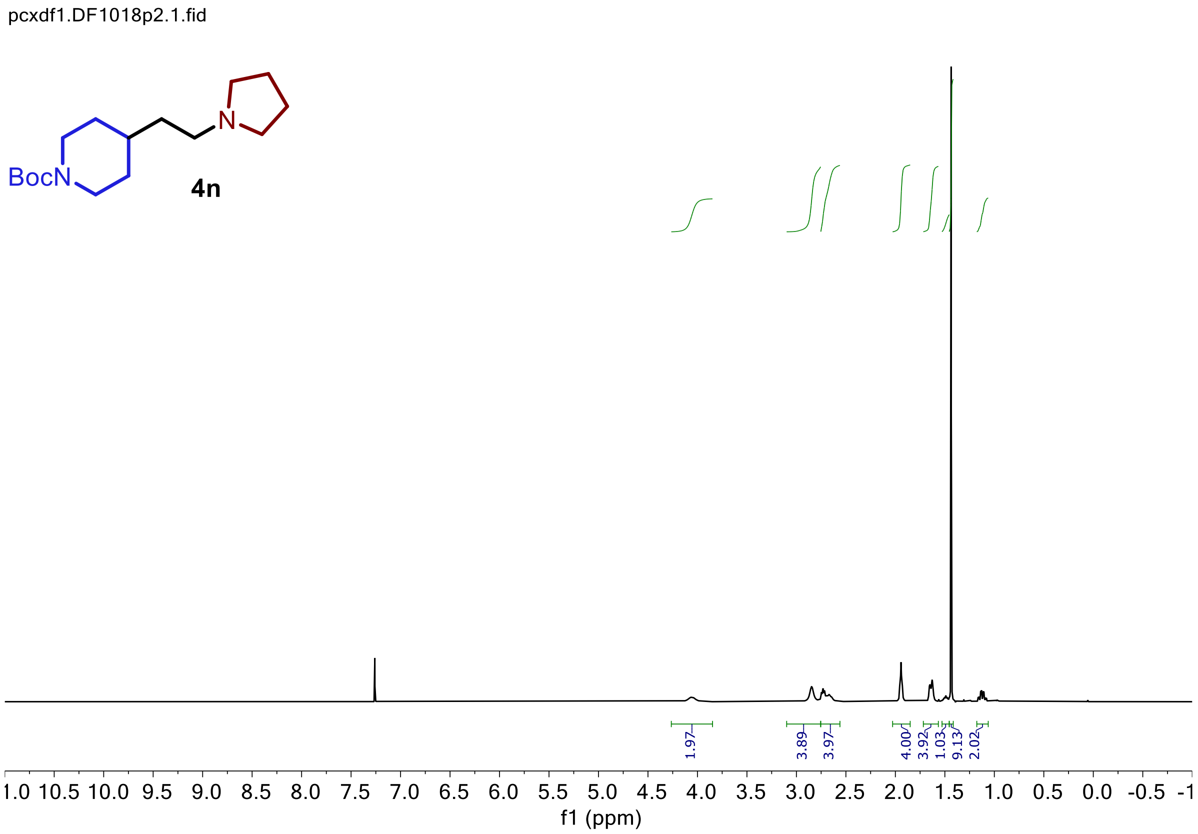

^{13}C-NMR ($CDCl_3$, 126 MHz)

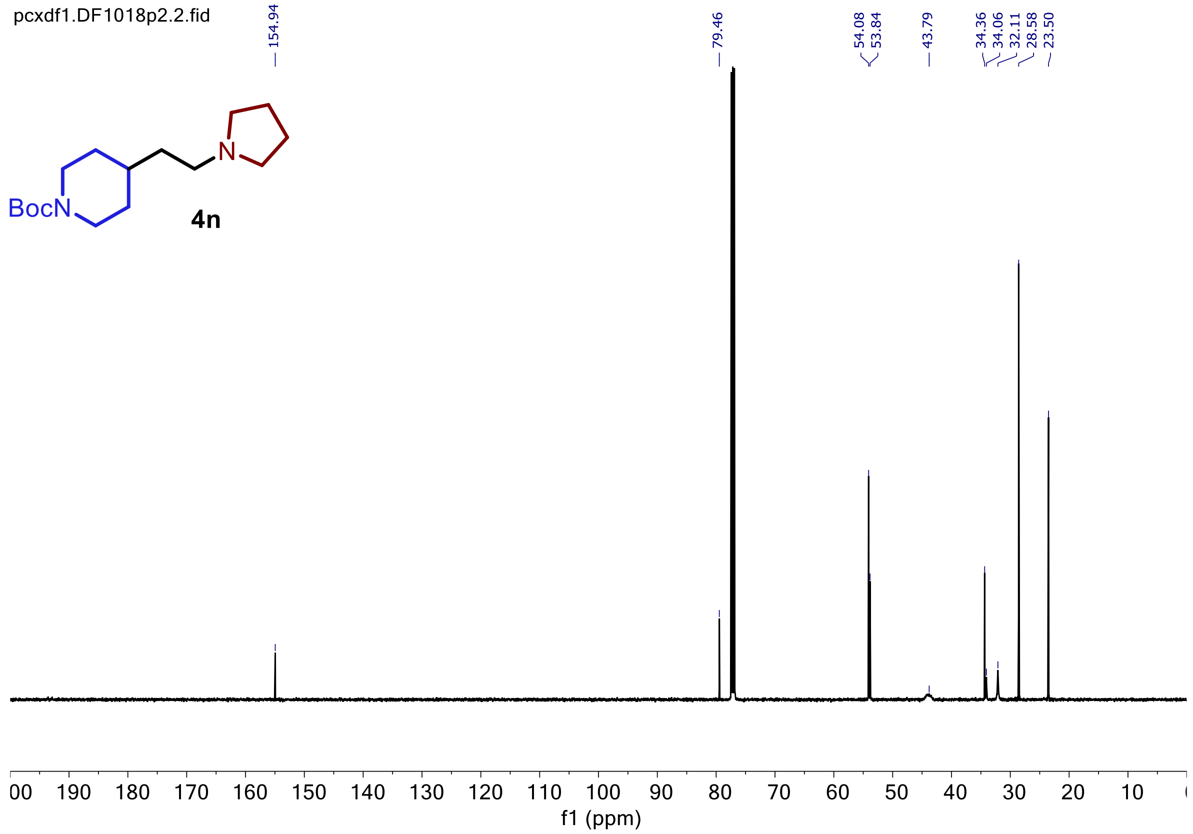

tert-butyl 4-(2-(phenylamino)ethyl)piperidine-1-carboxylate (4o)

^{1}H-NMR (CDCl$_3$, 500 MHz)

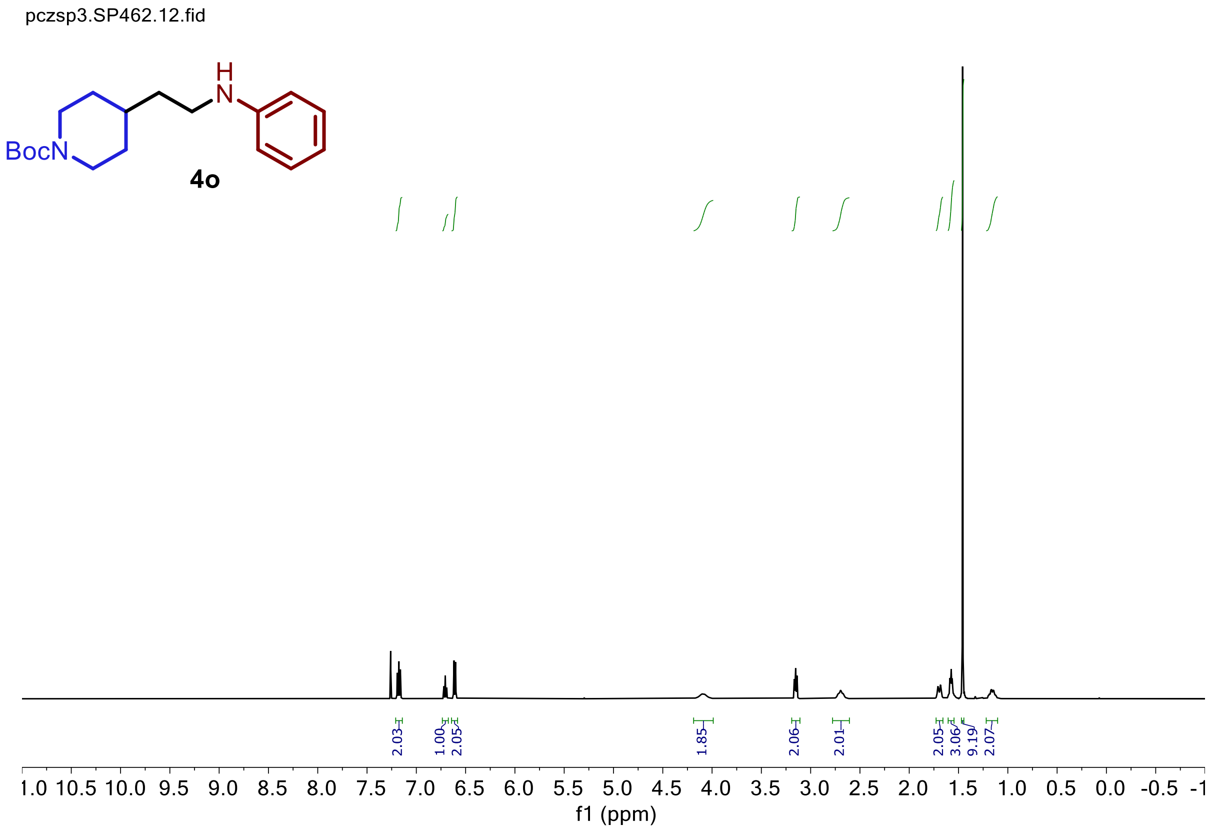

^{13}C-NMR (CDCl$_3$, 126 MHz)

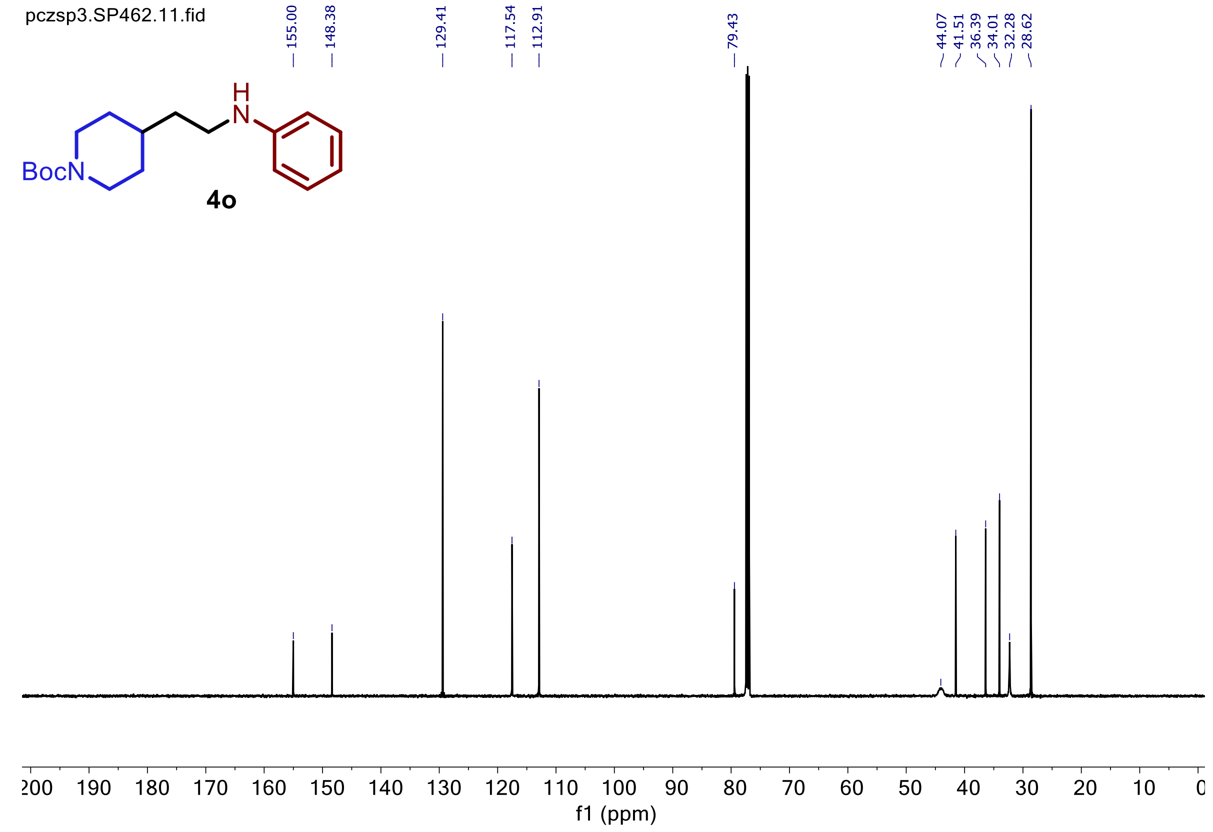

tert-butyl 4-(2-(((1_S_,2_S_)-1-hydroxy-1-phenylpropan-2-yl)(methyl)amino)ethyl)piperidine-1-carboxylate (4p)

^{1}H-NMR (CDCl$_3$, 500 MHz)

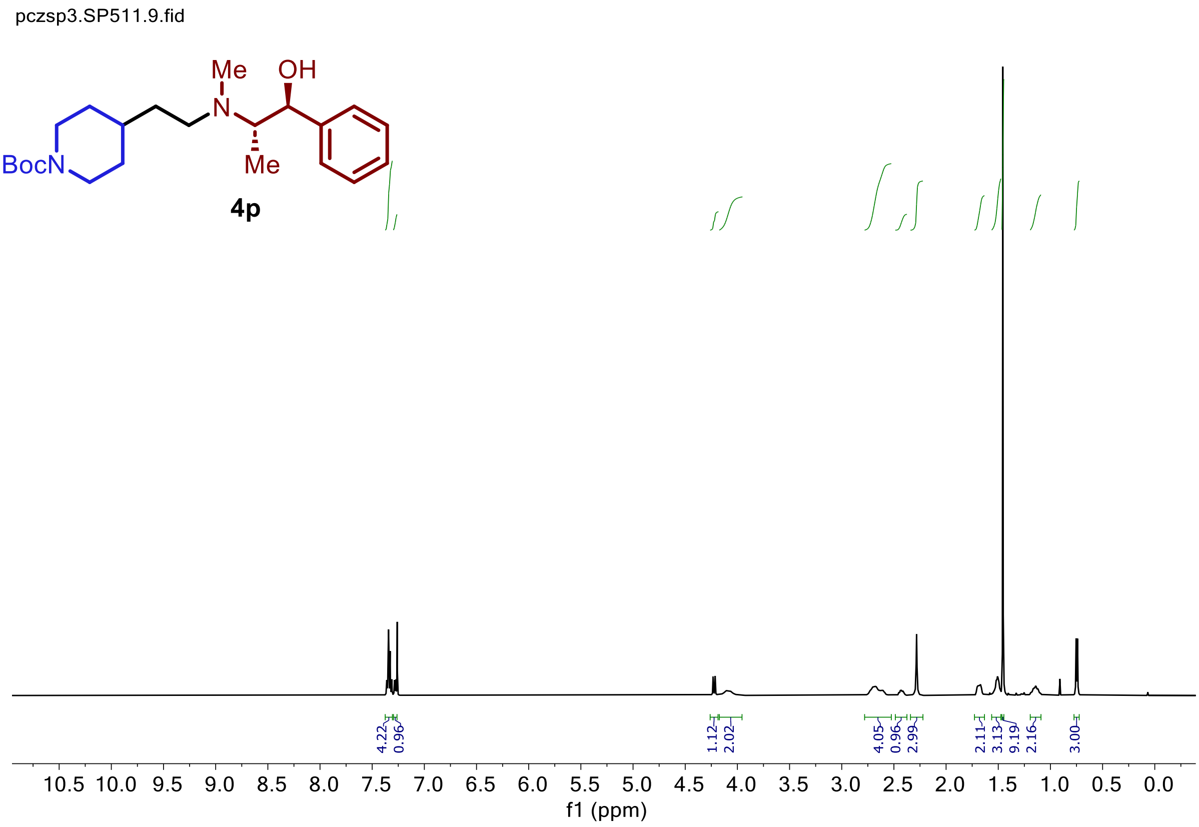

^{13}C-NMR (CDCl$_3$, 126 MHz)

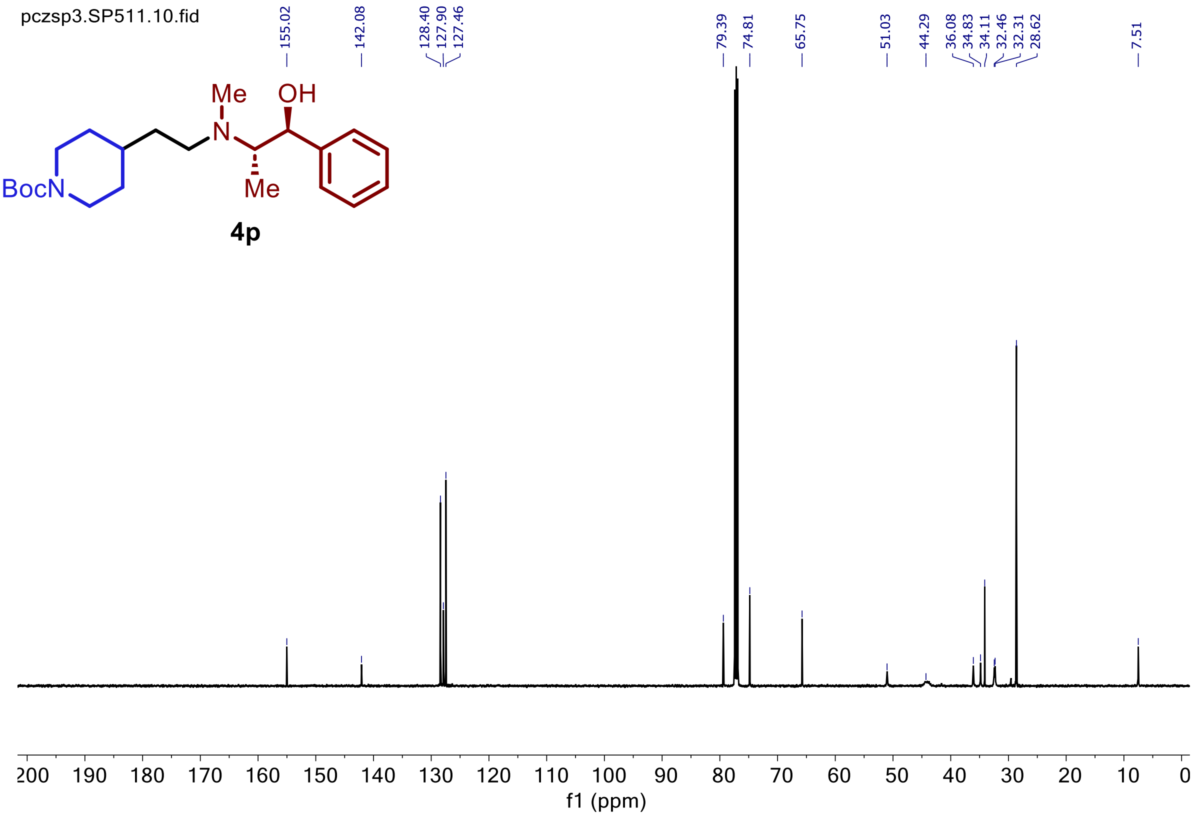

tert-butyl 2-(2-((2-hydroxyethyl)thio)ethyl)pyrrolidine-1-carboxylate (4q)

^{1}H-NMR ($CDCl_3$, 500 MHz)

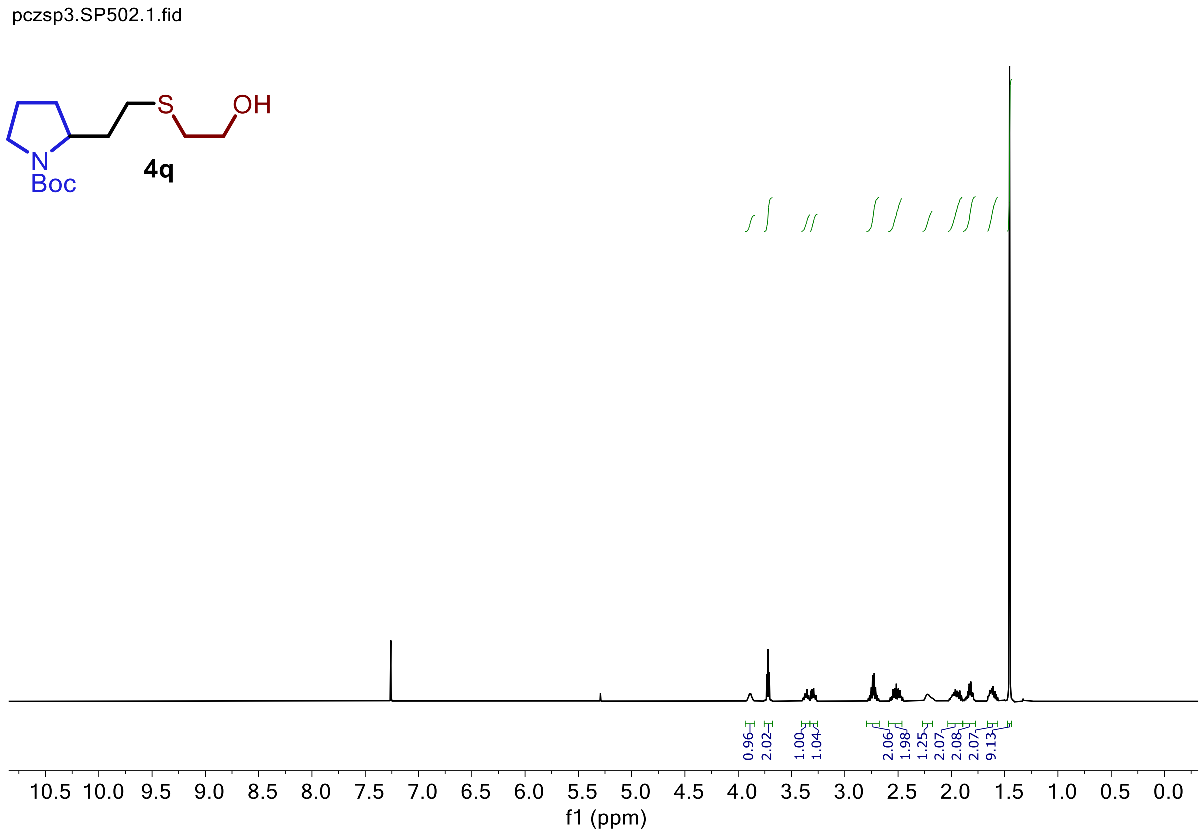

^{13}C-NMR ($CDCl_3$, 126 MHz)

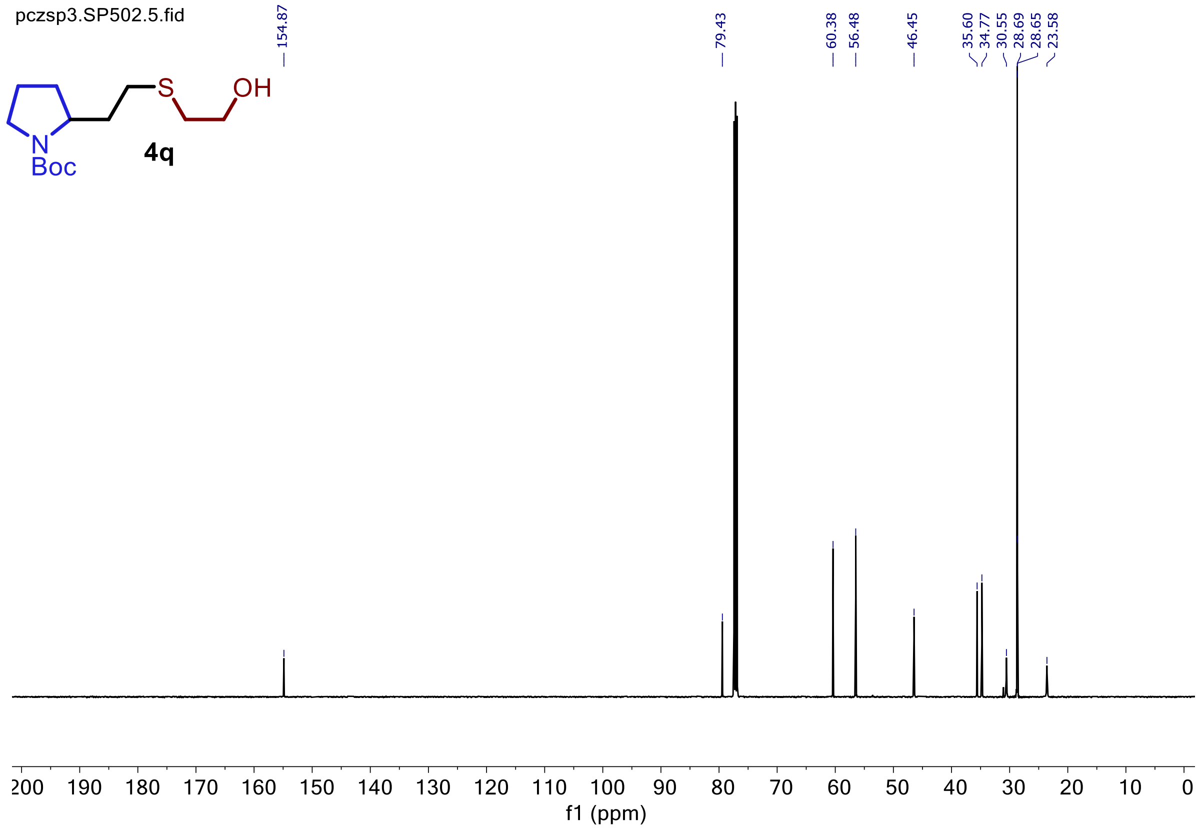

2-((2-(tetrahydrofuran-2-yl)ethyl)thio)ethan-1-ol (4r)

^{1}H-NMR (CDCl$_3$, 500 MHz)

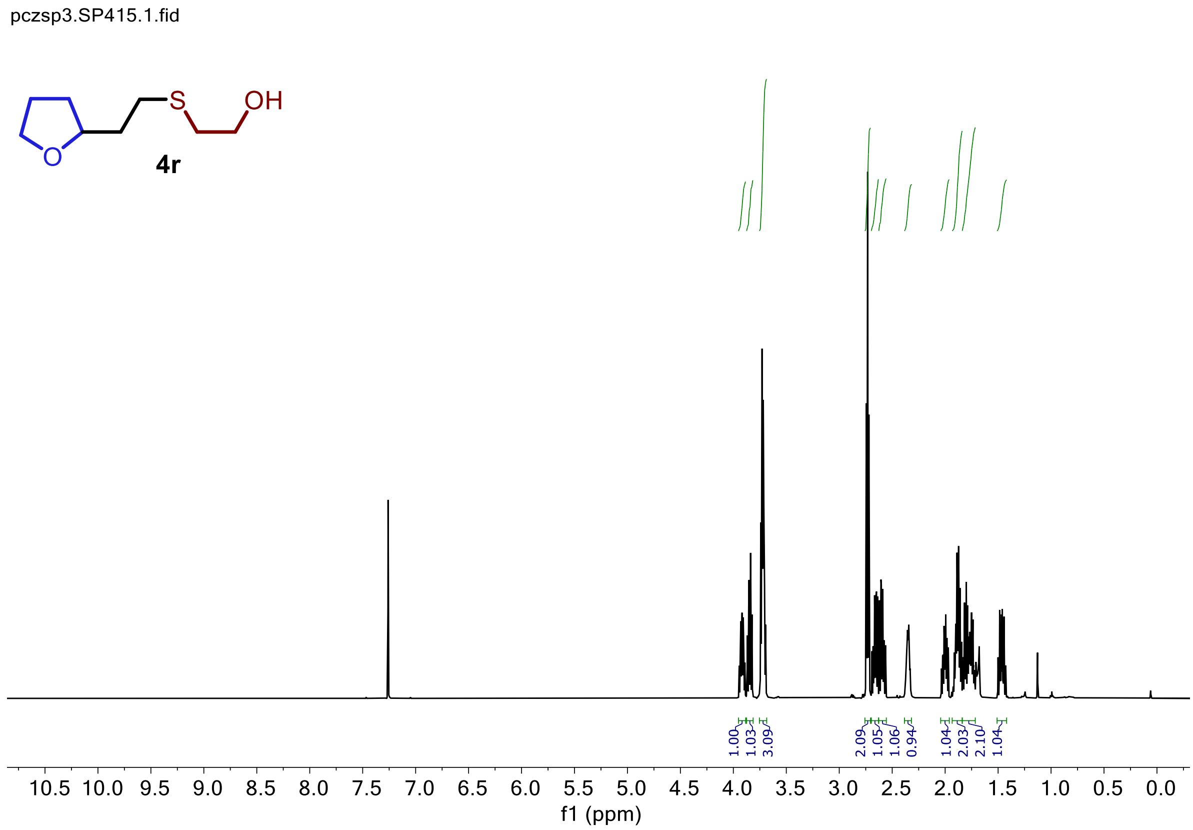

^{13}C-NMR (CDCl$_3$, 126 MHz)

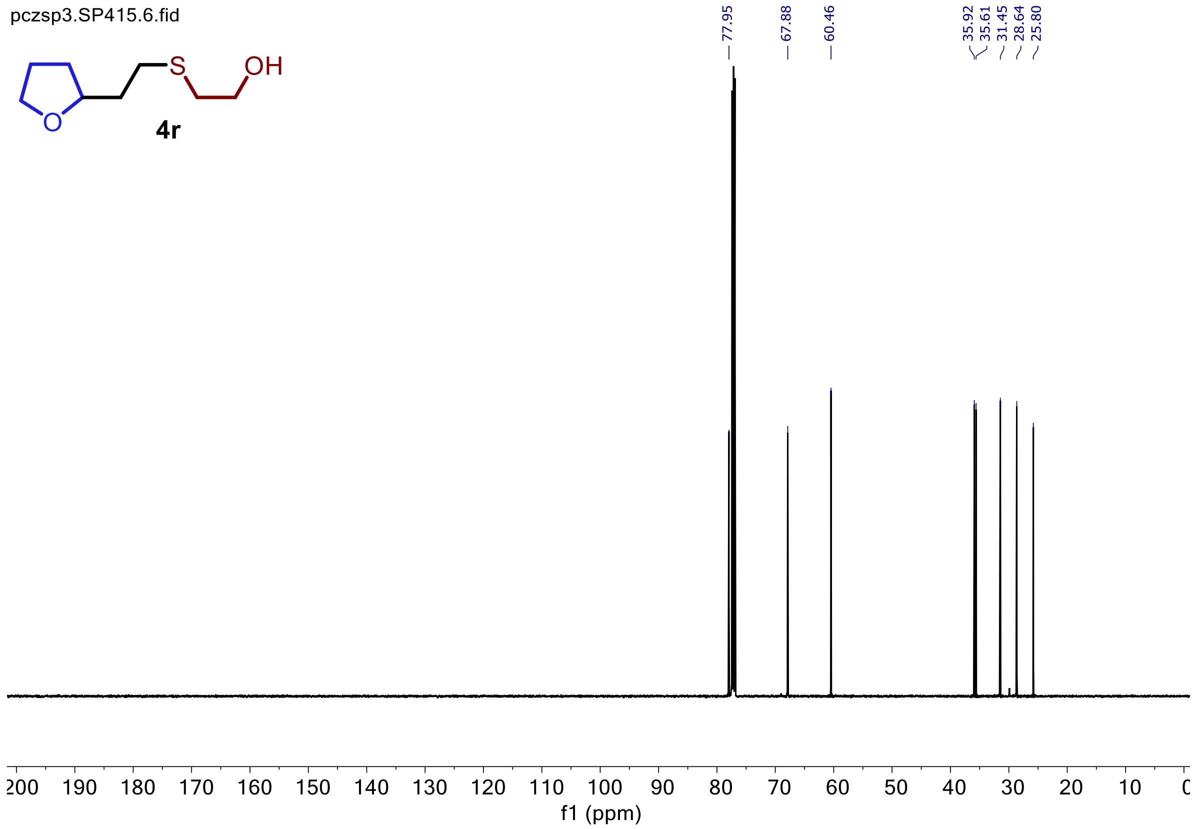

2-((2-(tetrahydro-2*H*-pyran-4-yl)ethyl)thio)ethan-1-ol (4s)

^{1}H-NMR ($CDCl_3$, 500 MHz)

pczsp3.SP436.10.fid

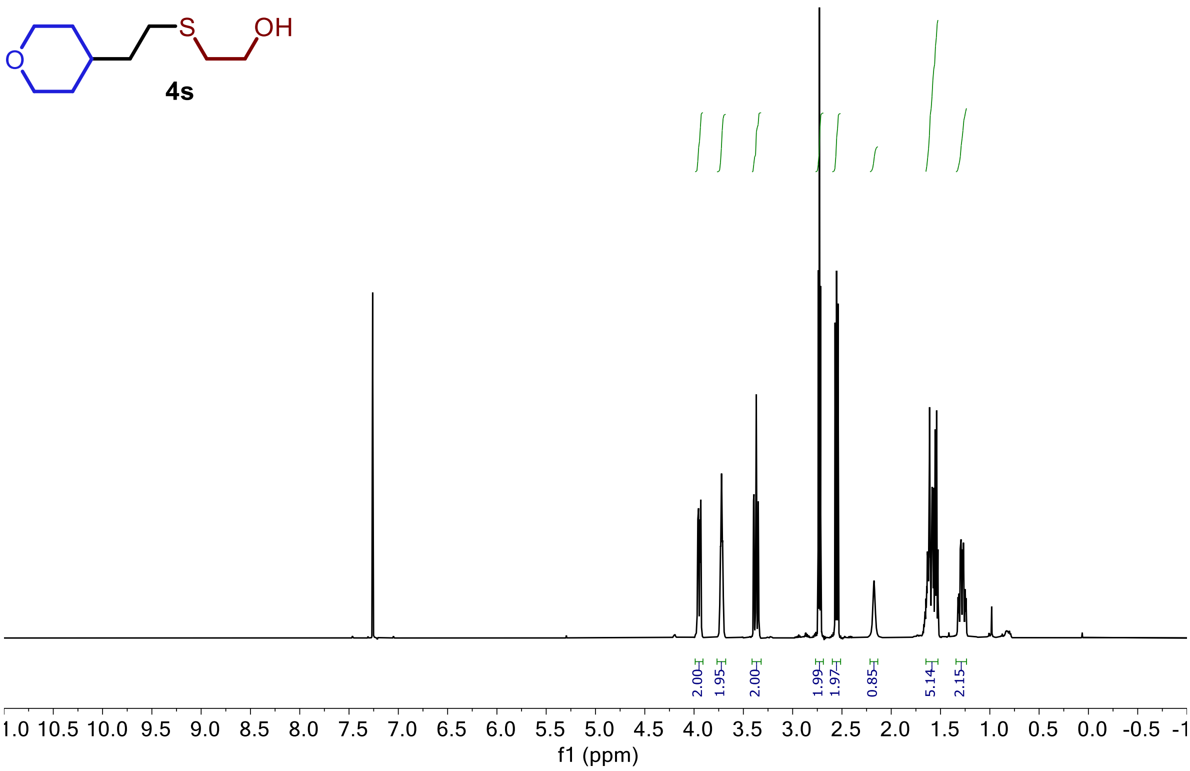

^{13}C-NMR ($CDCl_3$, 126 MHz)

pczsp3.SP436.6.fid

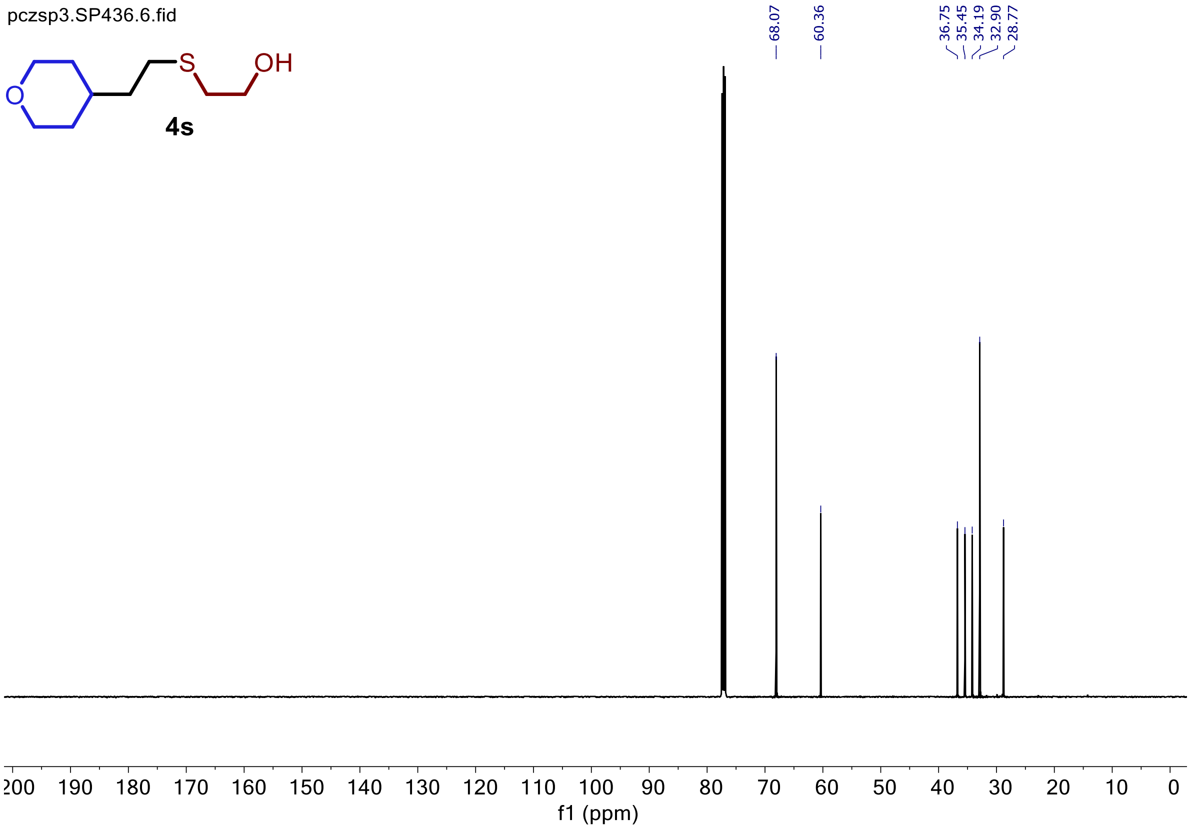

2-((2-cyclododecylethyl)thio)ethan-1-ol (4t)

^{1}H-NMR ($CDCl_3$, 500 MHz)

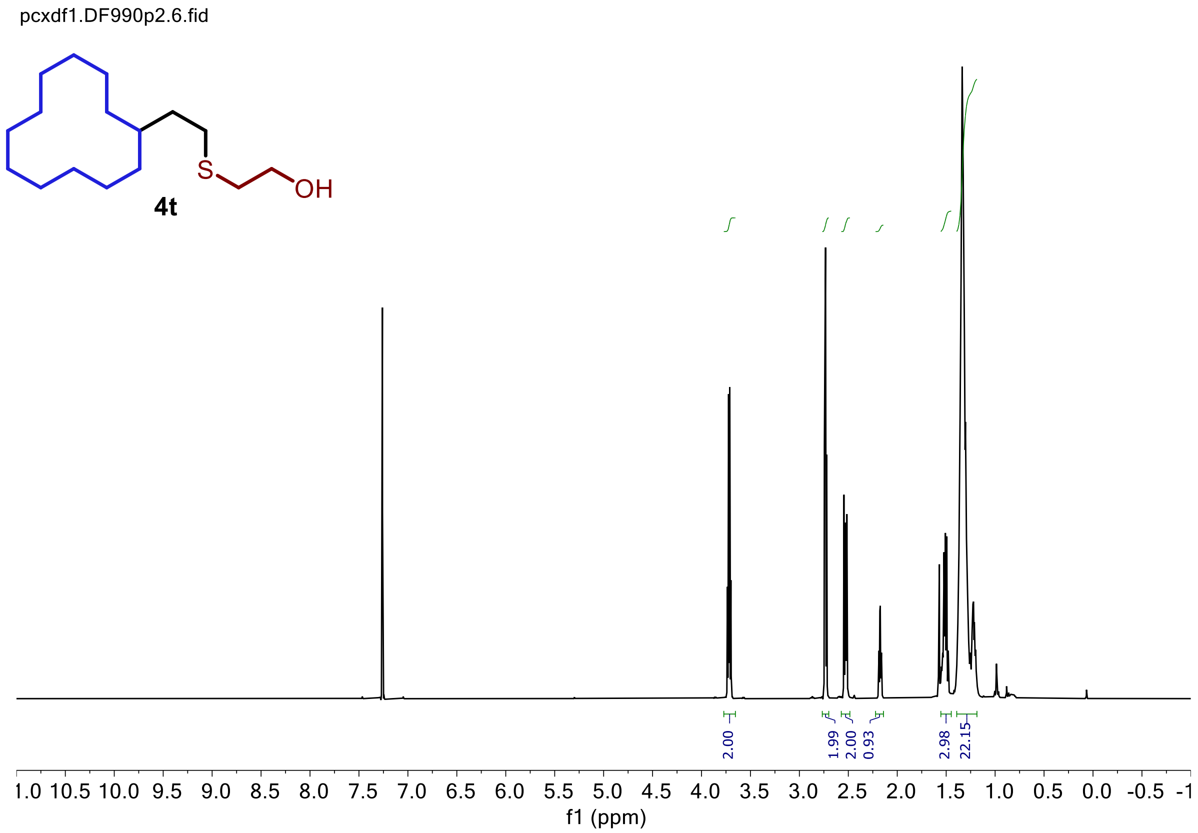

^{13}C-NMR ($CDCl_3$, 126 MHz)

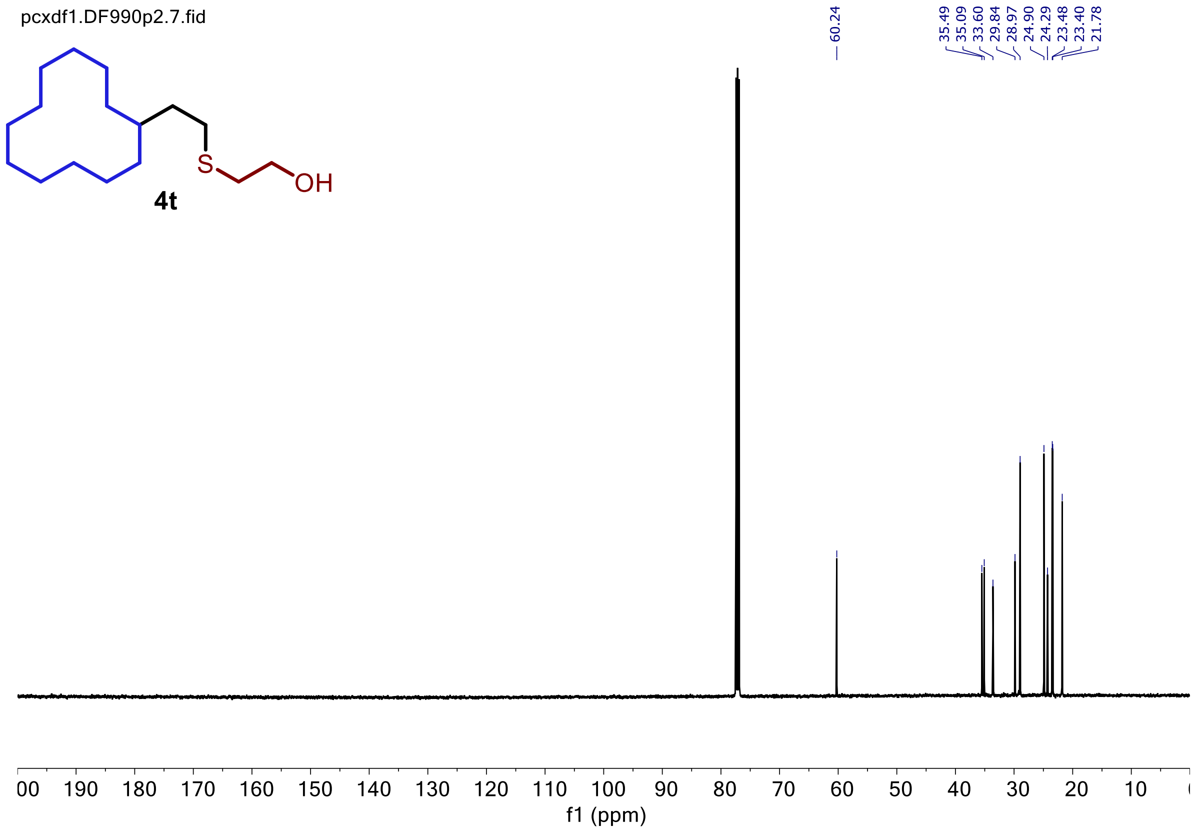

tert-butyl 2-(2-((2-hydroxyethyl)thio)ethyl)azetidine-1-carboxylate (4u)

^{1}H-NMR (CDCl$_3$, 500 MHz)

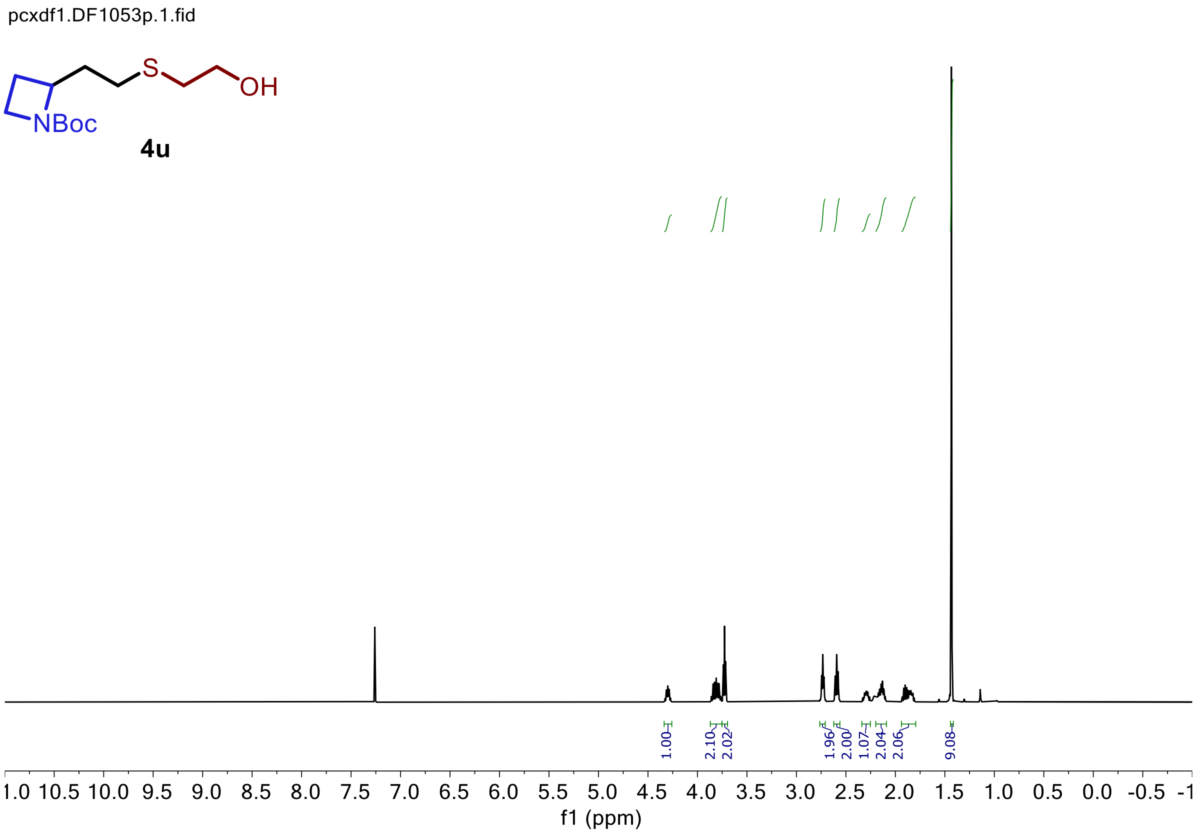

^{13}C-NMR (CDCl$_3$, 126 MHz)

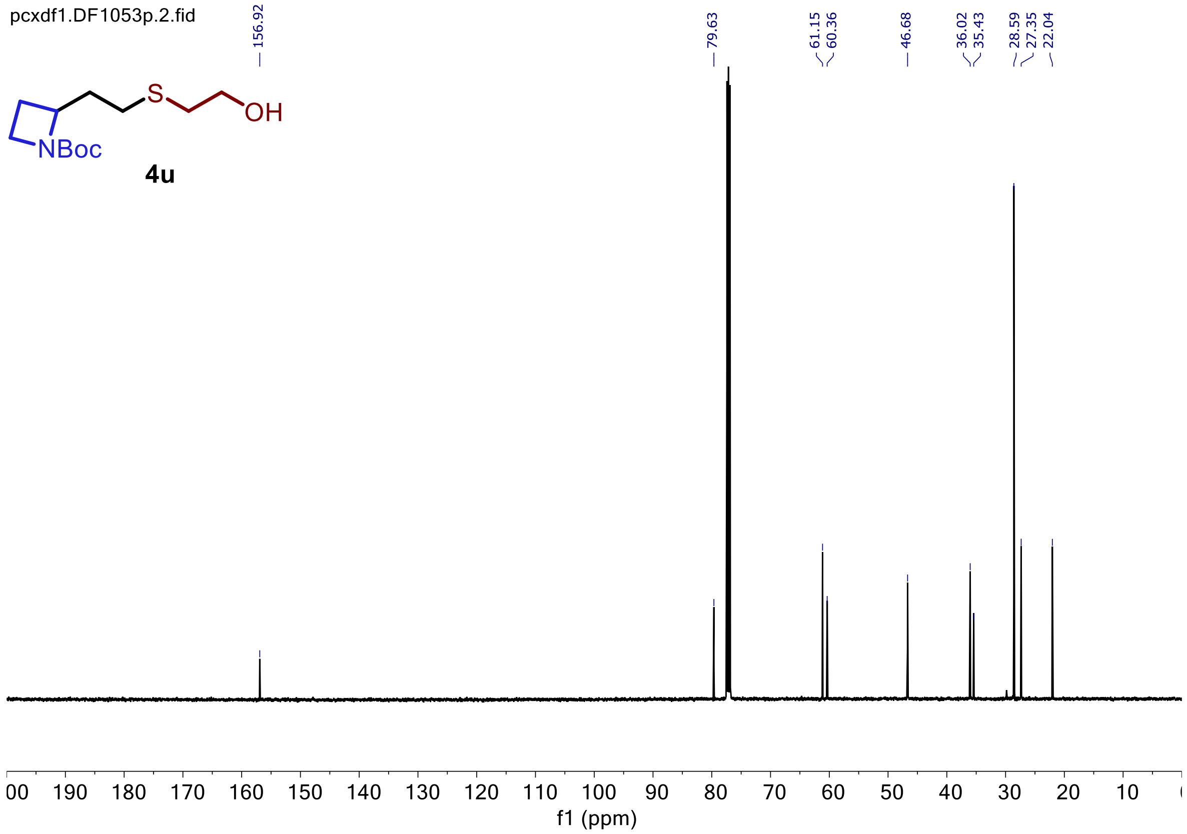

2-((2-((1*s*,3*s*)-adamantan-1-yl)ethyl)thio)ethan-1-ol (4v)

^{1}H-NMR ($CDCl_3$, 500 MHz)

pczsp3.SP4142nd.7.fid

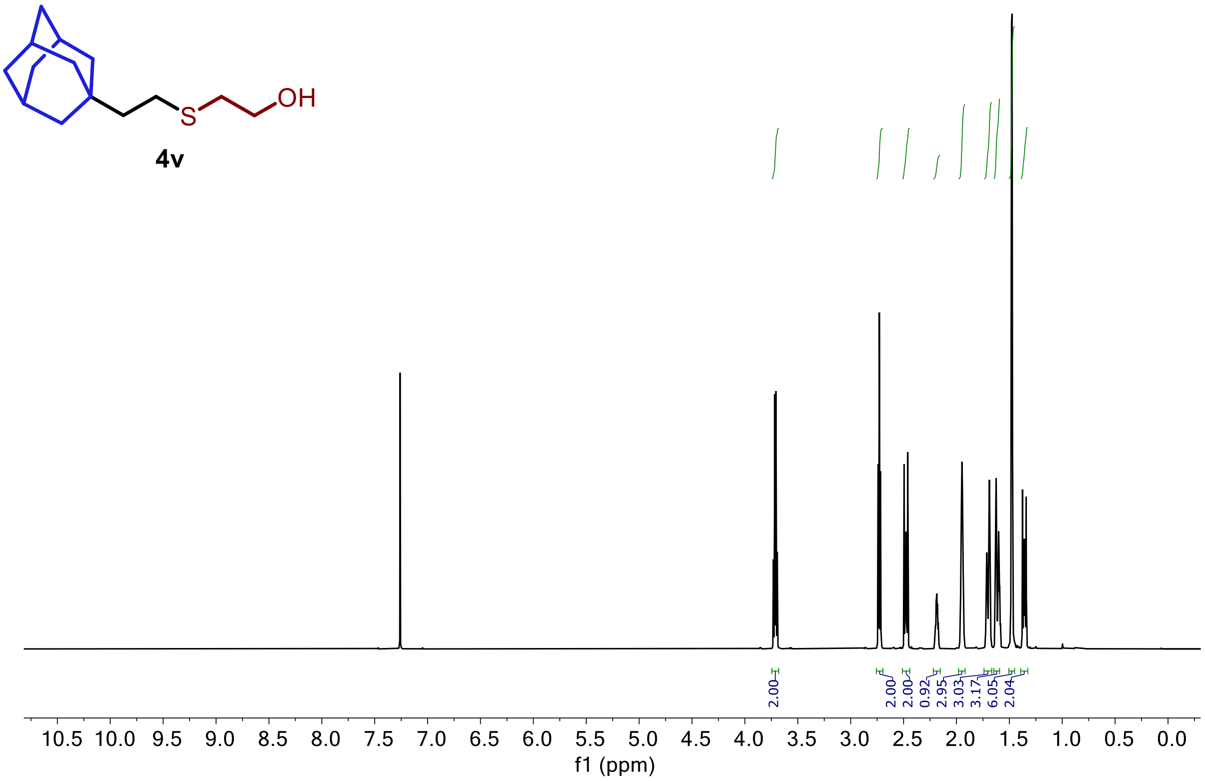

^{13}C-NMR ($CDCl_3$, 126 MHz)

pczsp3.SP4142nd.6.fid

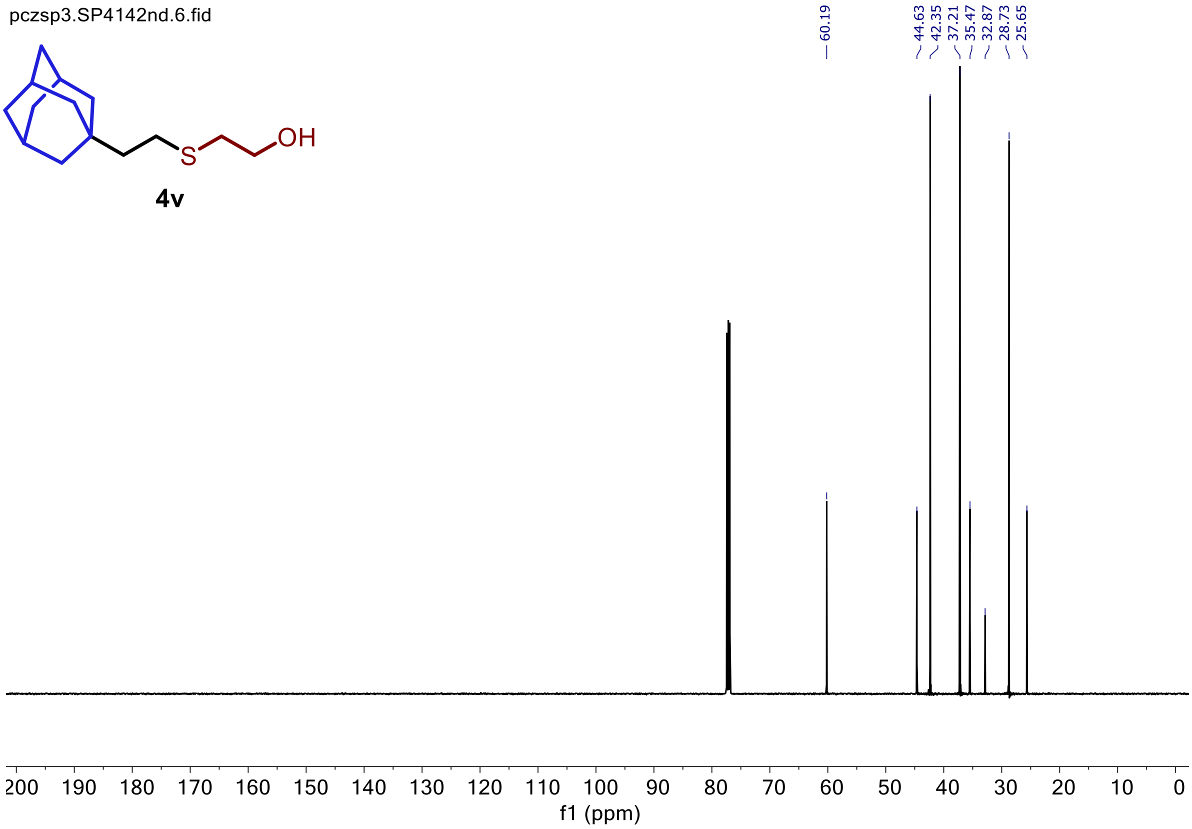

2-(2-((2-hydroxyethyl)thio)ethyl)-2,5,7,8-tetramethylchroman-6-ol (4w)

^{1}H-NMR ($CDCl_3$, 500 MHz)

pcxdf1.DF1000p.1.fid

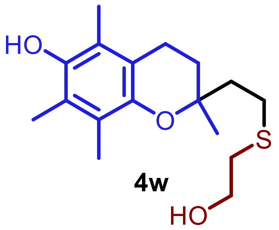

4w

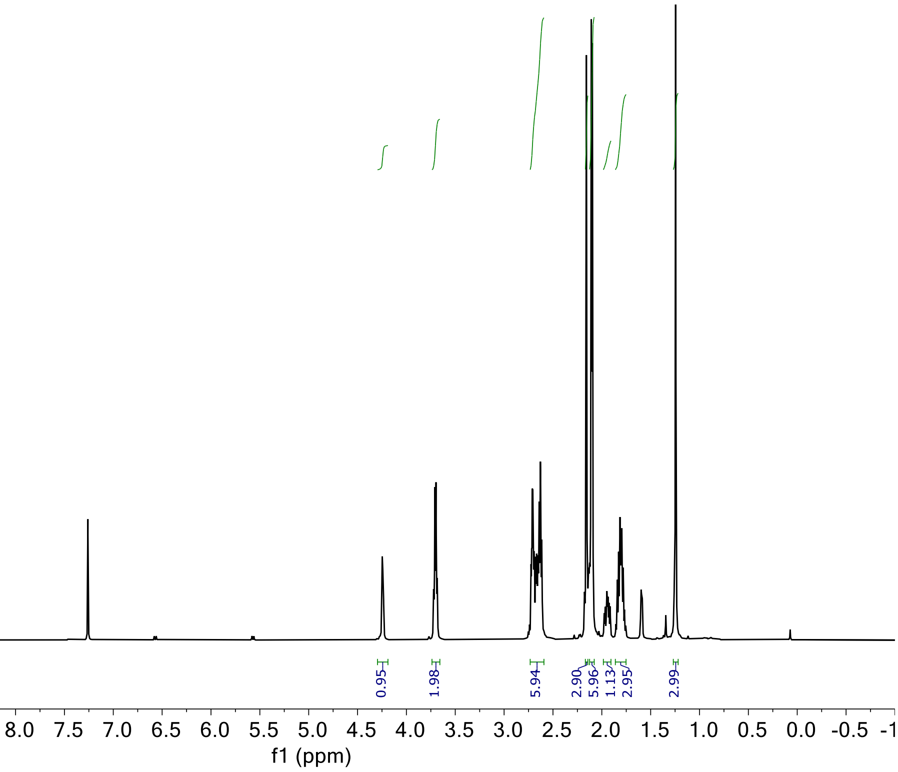

^{13}C-NMR ($CDCl_3$, 126 MHz)

pcxdf1.DF1000p.2.fid

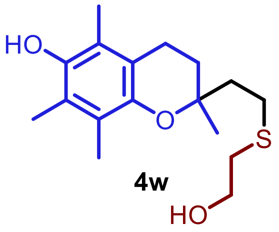

4w

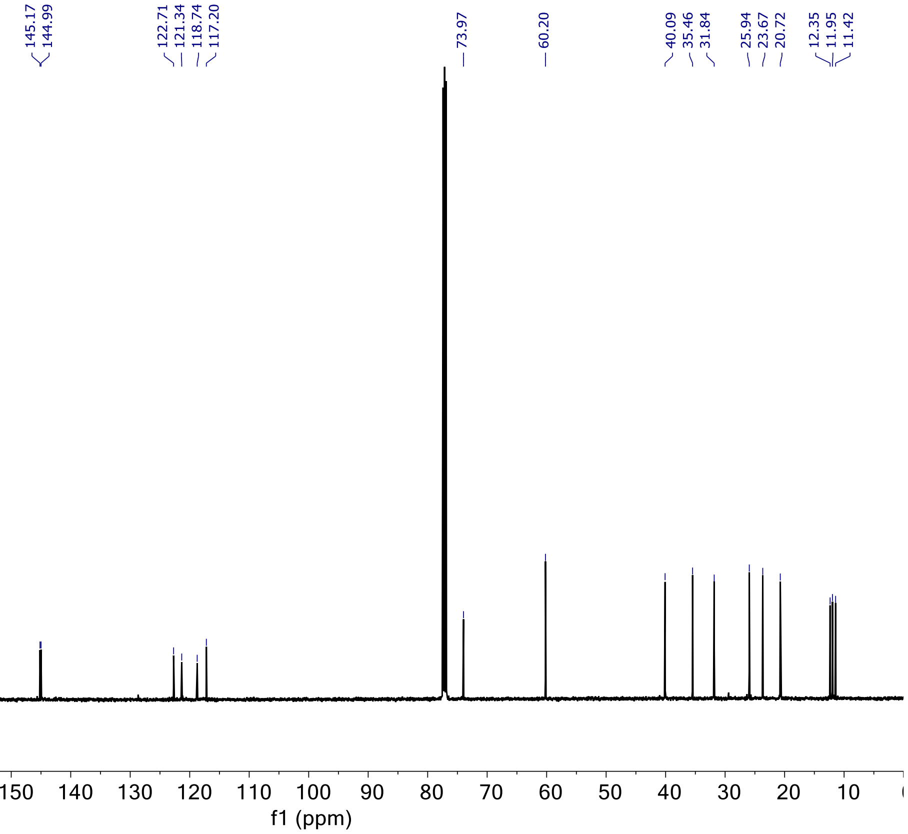

4-chloro-N-(4-((4-((2-hydroxyethyl)thio)-2-methylbutan-2-yl)oxy)phenethyl)benzamide (4x)

^{1}H-NMR (CDCl$_3$, 500 MHz)

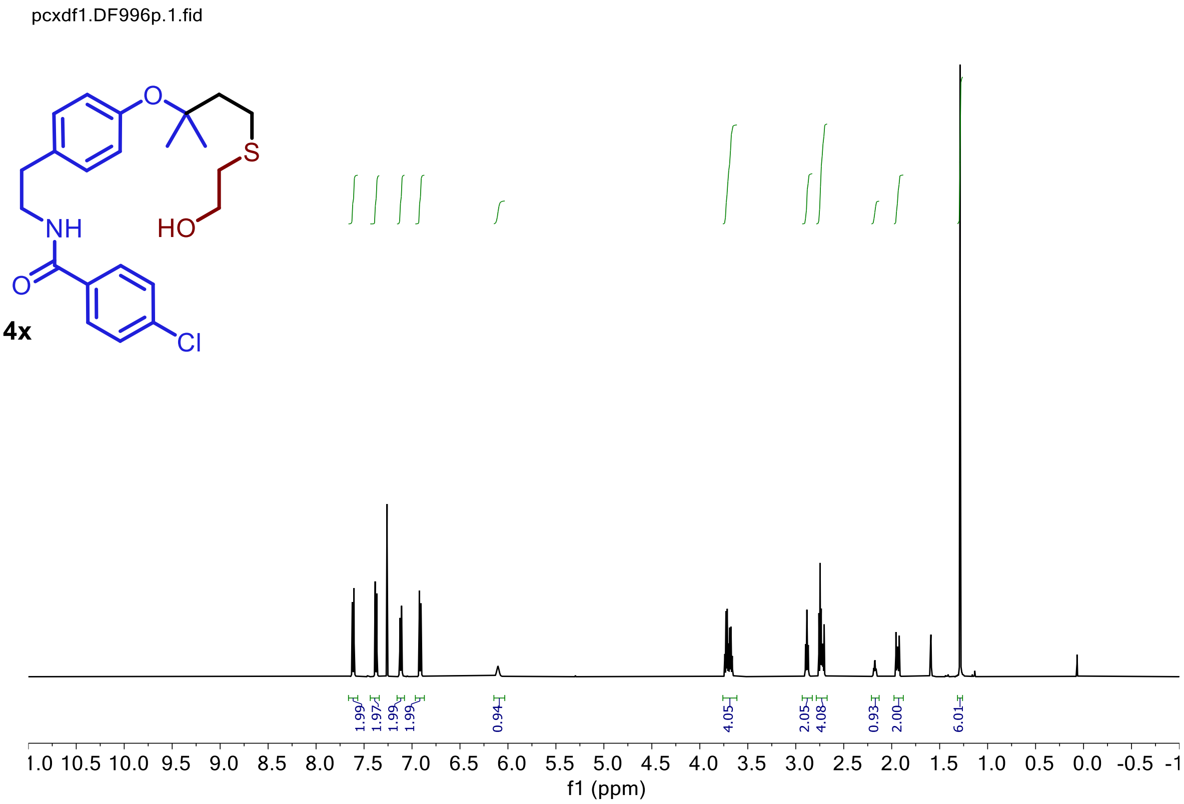

^{13}C-NMR (CDCl$_3$, 126 MHz)

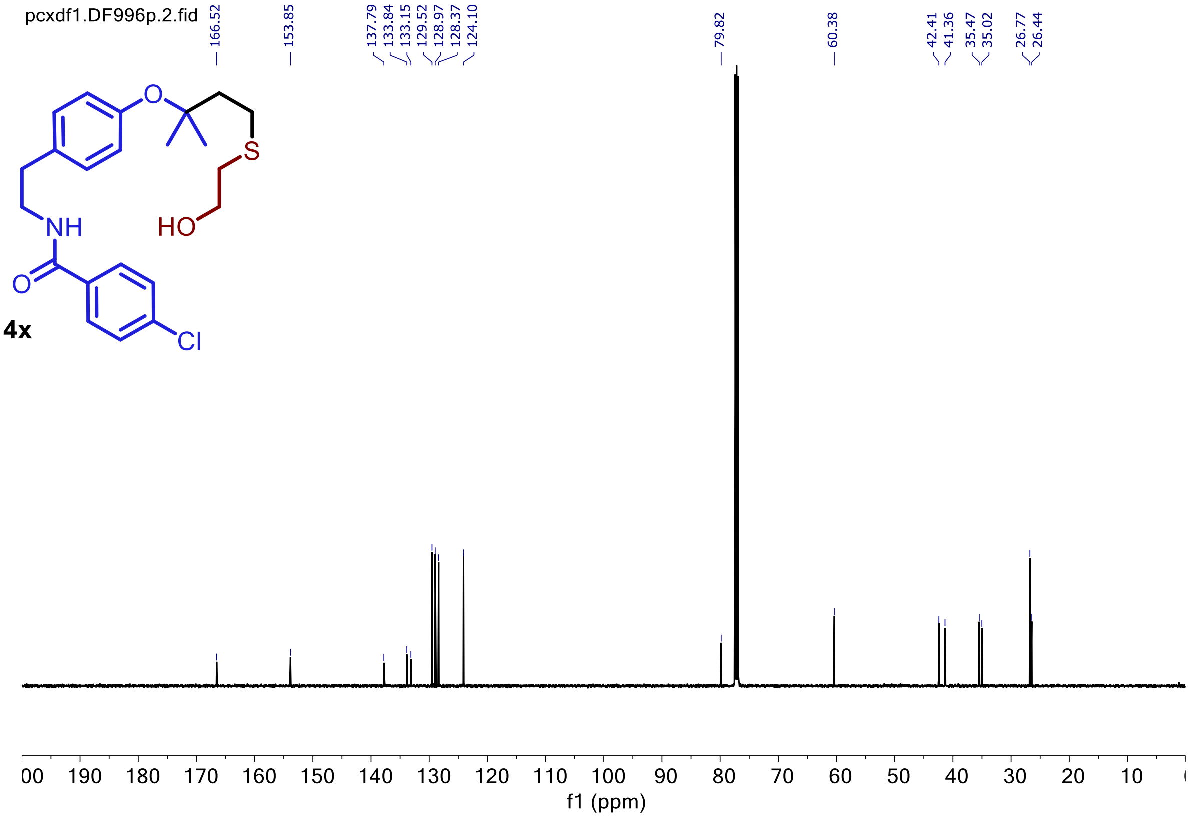

2-((6-(2,5-dimethylphenoxy)-3,3-dimethylhexyl)thio)ethan-1-ol (4y)

^{1}H-NMR ($CDCl_3$, 500 MHz)

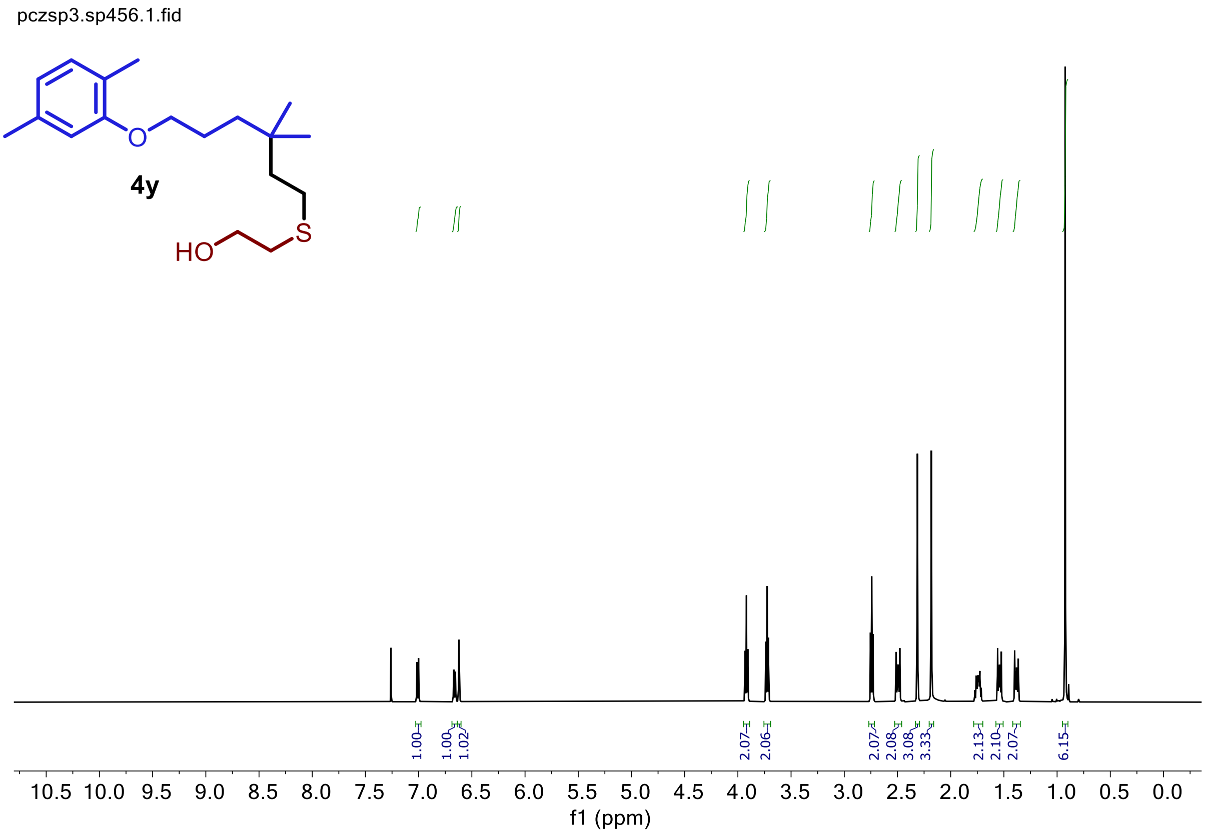

^{13}C-NMR ($CDCl_3$, 126 MHz)

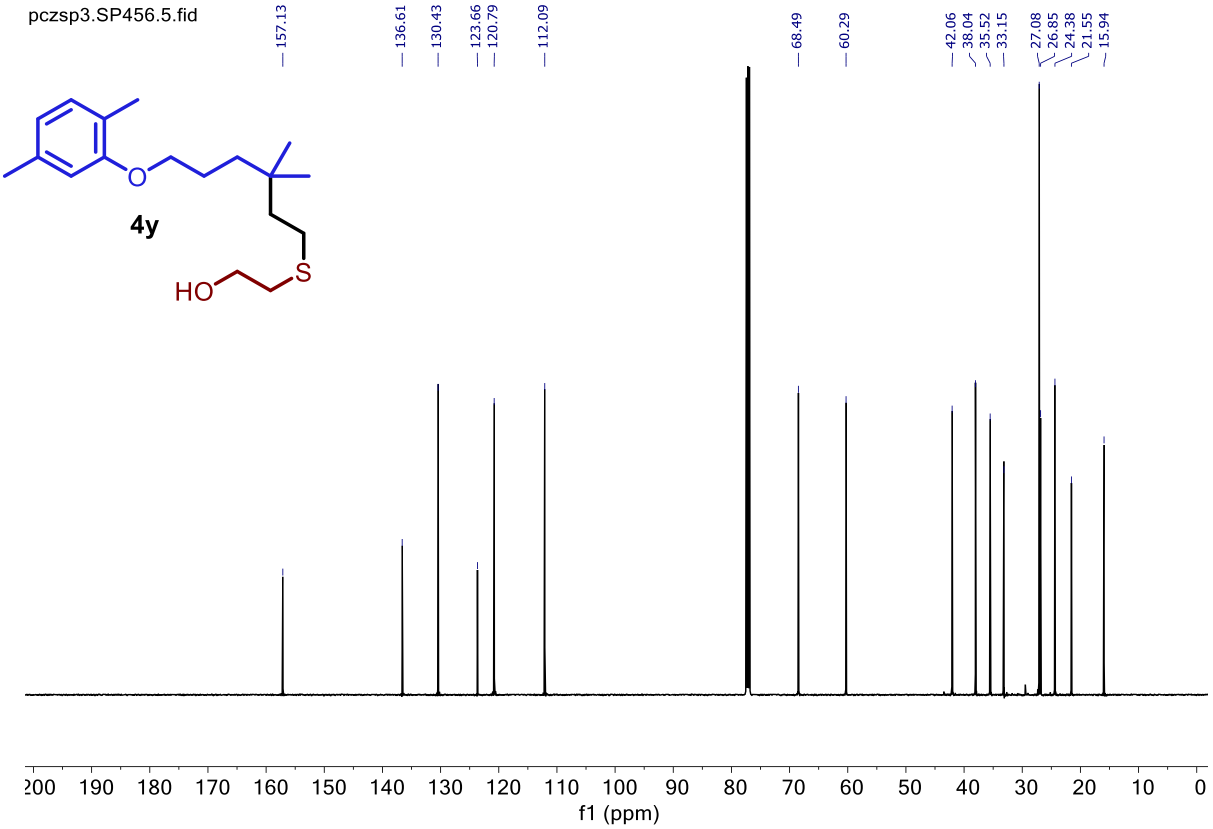

(3*S*,4a*R*,6a*R*,6b*S*,8a*R*,12a*R*,14a*R*,14b*R*)-8a-(2-((2-hydroxyethyl)thio)ethyl)-4,4,6a,6b,11,11,14b-heptamethyl-1,2,3,4,4a,5,6,6a,6b,7,8,8a,9,10,11,12,12a,14,14a,14b-icosahydropicen-3-ol (4z)

^{1}H-NMR ($CDCl_3$, 500 MHz)

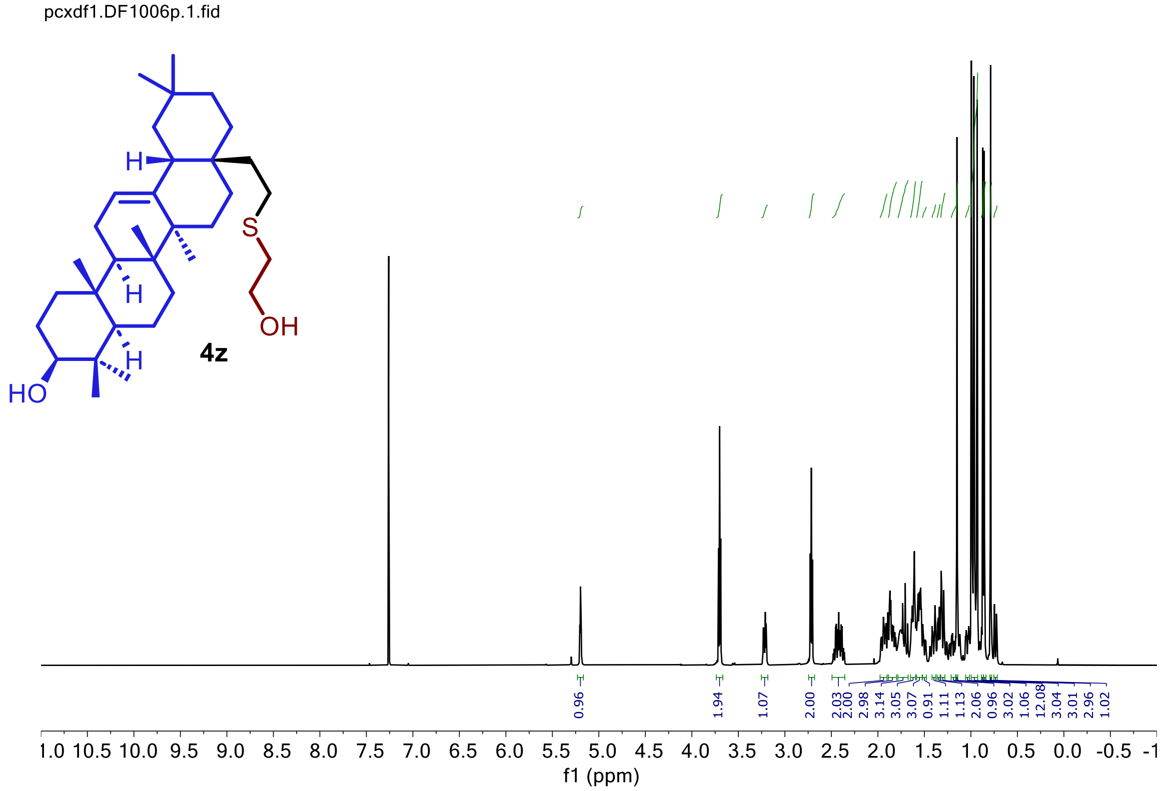

^{13}C-NMR ($CDCl_3$, 126 MHz)

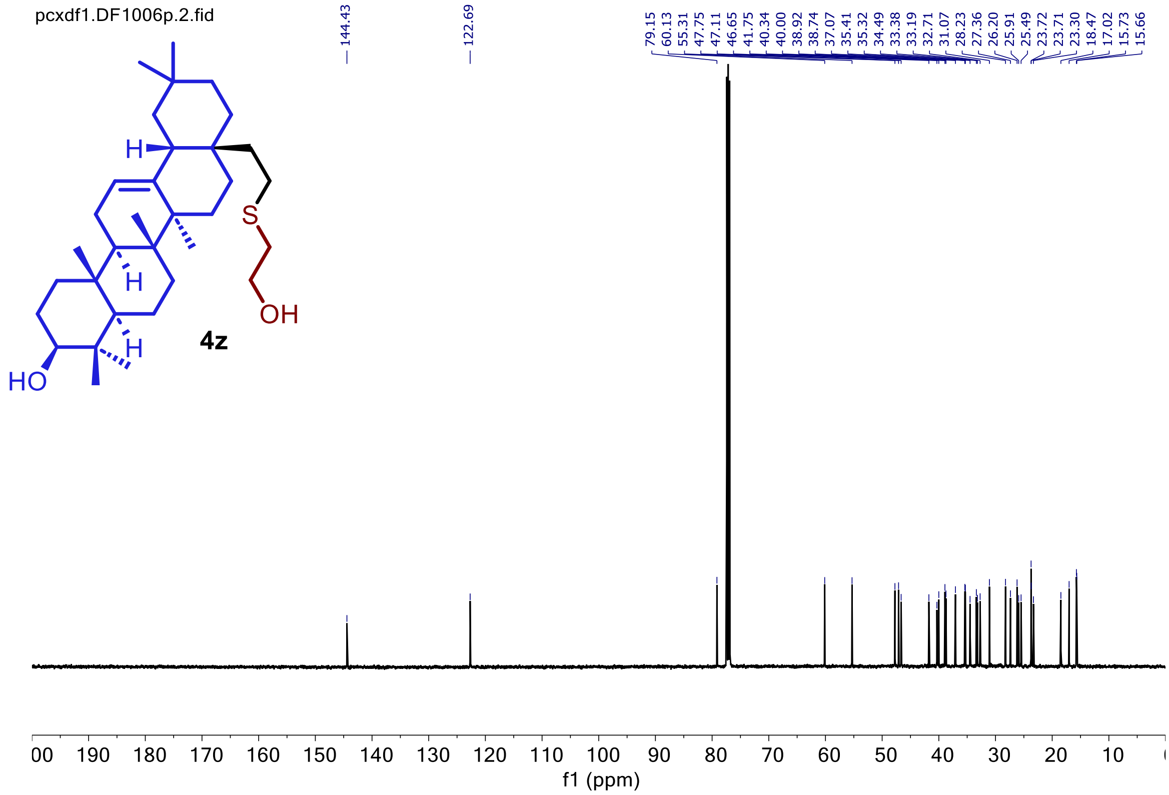

(4a*R*,6a*S*,6b*R*,8a*R*,10*S*,12a*S*,12b*R*,14b*R*)-10-hydroxy-2-(2-((2-hydroxyethyl)thio)ethyl)-2,4a,6a,6b,9,9,12a-heptamethyl-1,3,4,4a,5,6,6a,6b,7,8,8a,9,10,11,12,12a,12b,14b-octadecahydropicen-13(2*H*)-one (4aa)

^{1}H-NMR ($CDCl_3$, 500 MHz)

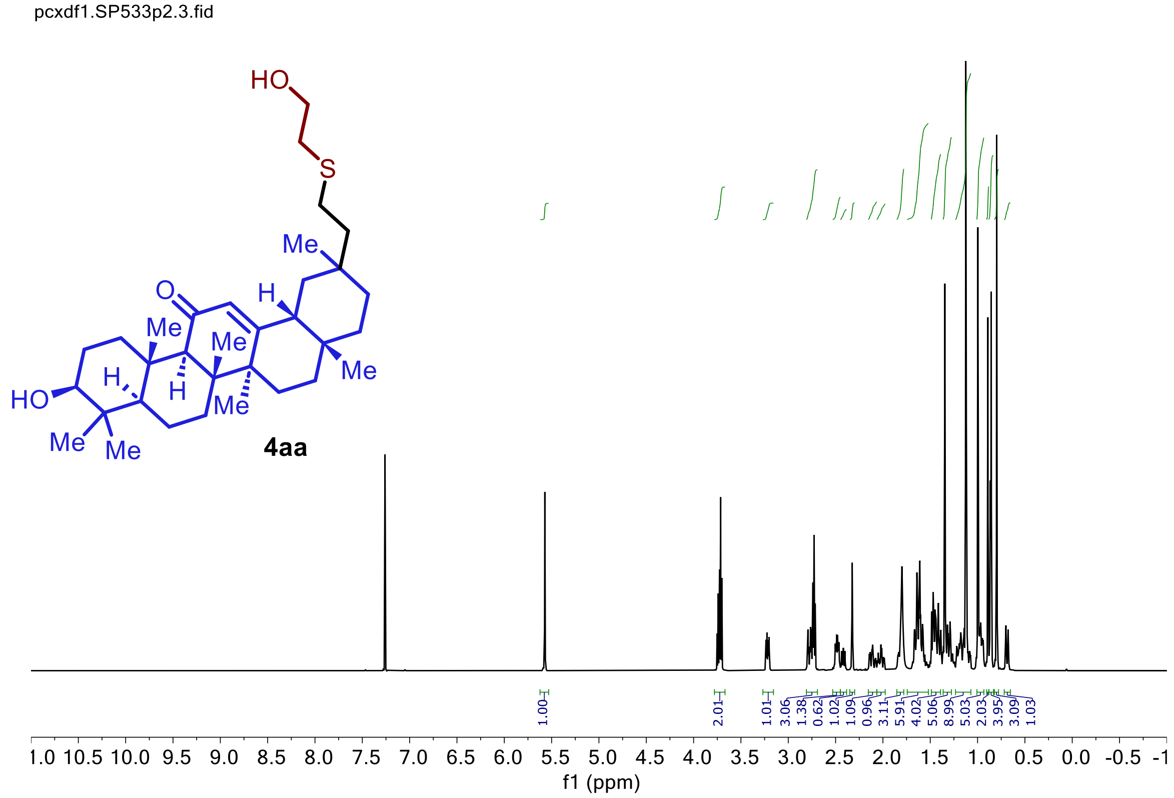

^{13}C-NMR ($CDCl_3$, 126 MHz)

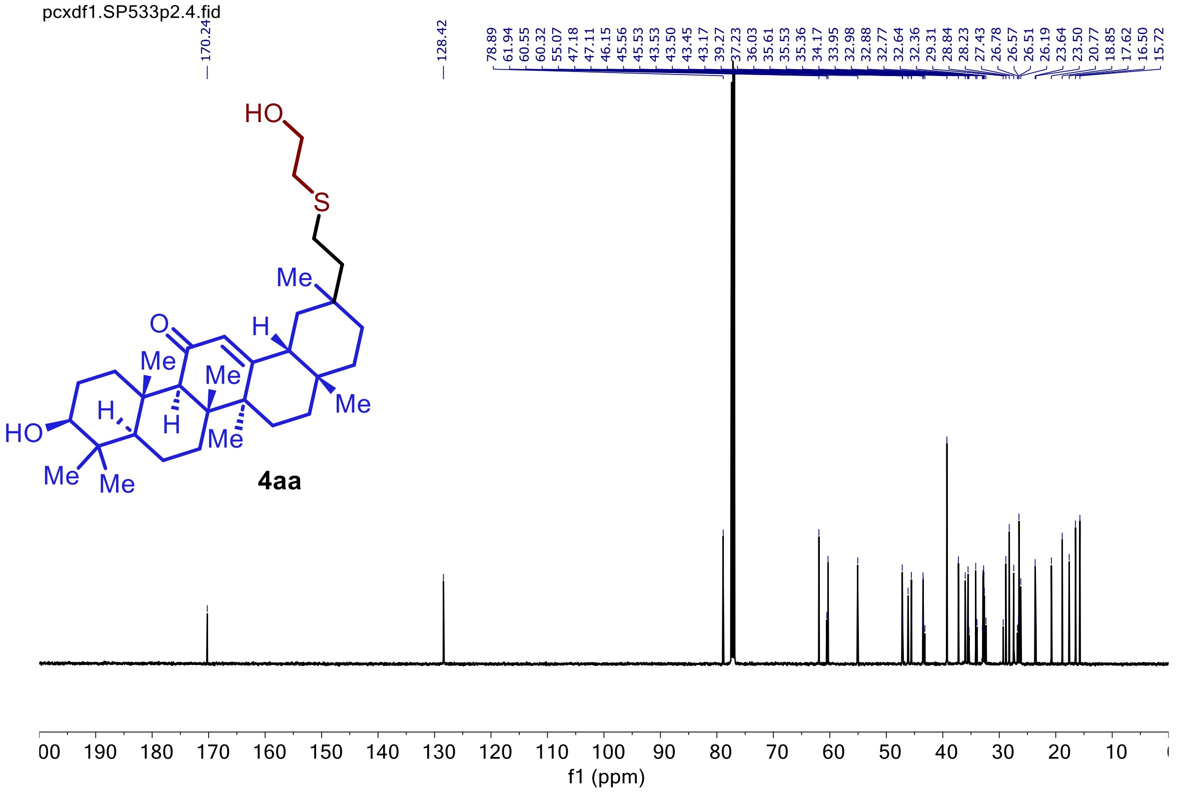

Supplement: Supplementary file 1 — ja2c12699_si_001.pdf [file ja2c12699_si_001.pdf]
